# The challenges of pedigree dog health: approaches to combating inherited disease

**DOI:** 10.1186/s40575-015-0014-9

**Published:** 2015-02-11

**Authors:** Lindsay L Farrell, Jeffrey J Schoenebeck, Pamela Wiener, Dylan N Clements, Kim M Summers

**Affiliations:** The Roslin Institute and the Royal (Dick) School of Veterinary Studies, University of Edinburgh, Easter Bush Campus, Midlothian, EHG25 9RG UK

**Keywords:** Dog, Inherited disorders, Health

## Abstract

The issue of inherited disorders and poor health in pedigree dogs has been widely discussed in recent years. With the advent of genome-wide sequencing technologies and the increasing development of new diagnostic DNA disease tests, the full extent and prevalence of inherited disorders in pedigree dogs is now being realized. In this review we discuss the challenges facing pedigree dog breeds: the common pitfalls and problems associated with combating single gene mediated disorders, phenotypic selection on complex disorders, and ways of managing genetic diversity. Breeding strategies incorporating screening schemes have been shown to be successful in significantly reducing the prevalence of an inherited disorder and improving the overall health in certain breeds. However, with 215 breeds officially recognized by the Kennel Club in the United Kingdom and 396 inherited disorders currently identified, many breeds have reached the point at which successfully breeding away from susceptible individuals at a population-wide scale will require new genomic selection strategies in combination with currently available breeding schemes. Whilst DNA-based tests identifying disease causing mutation(s) remain the most informative and effective approach for single gene disorder disease management, they must be used along with current screening schemes, genomic selection, and pedigree information in breeding programs in the effort to maintain genetic diversity while also significantly reducing the number of inherited disorders in pedigree dogs.

**Electronic supplementary material:**

The online version of this article (doi:10.1186/s40575-015-0014-9) contains supplementary material, which is available to authorized users.

## Lay summary

Humans and dogs have co-existed for thousands of years. Increasingly, over the last few centuries, many pedigree breeds have been generated based on selection for particular physical and/or behavioral characteristics, which have been fixed and maintained by inbreeding within closed familial lines.

The development of such pedigree dog breeds can be both a blessing and a curse: desirable features are rigidly retained, but sometimes, undesirable disease-causing genes can be inadvertently fixed within the breed.

Such diseases can reveal themselves only when two copies of the faulty version of the gene are inherited (recessive). Furthermore, if a Champion Sire is carrying such a disease gene, it can quickly spread across the whole breed. Similarly, if a breed is expanded from a small number of founder dogs, and one or more of these carry disease genes, again the disease frequency is likely to increase in the growing population. Sadly, some extreme forms of breed characteristics with a genetic basis can also contribute to issues of health and welfare.

This review discusses, in an objective and dispassionate way, the background behind inherited genetic diseases in pedigree dogs and how breeding strategies and genetic testing can be helpful in combating and reducing disease frequency, whilst also maintaining genetic diversity within each breed. The strengths and weaknesses of such approaches are also discussed.

## Introduction

Dogs were first domesticated more than 10,000 years ago [1-4] although exactly when and where is still debated. Since then, humans have enjoyed a long parallel history with dogs during our own progression from hunter-gatherers and then farmers, to modern city dwellers. Historically dogs lived in close proximity to humans and were used as working animals to herd livestock, hunt, and guard the home, and it is only recently that dogs have made the shift towards companion animals.

### Classification of dog breeds

**Figure 1:**
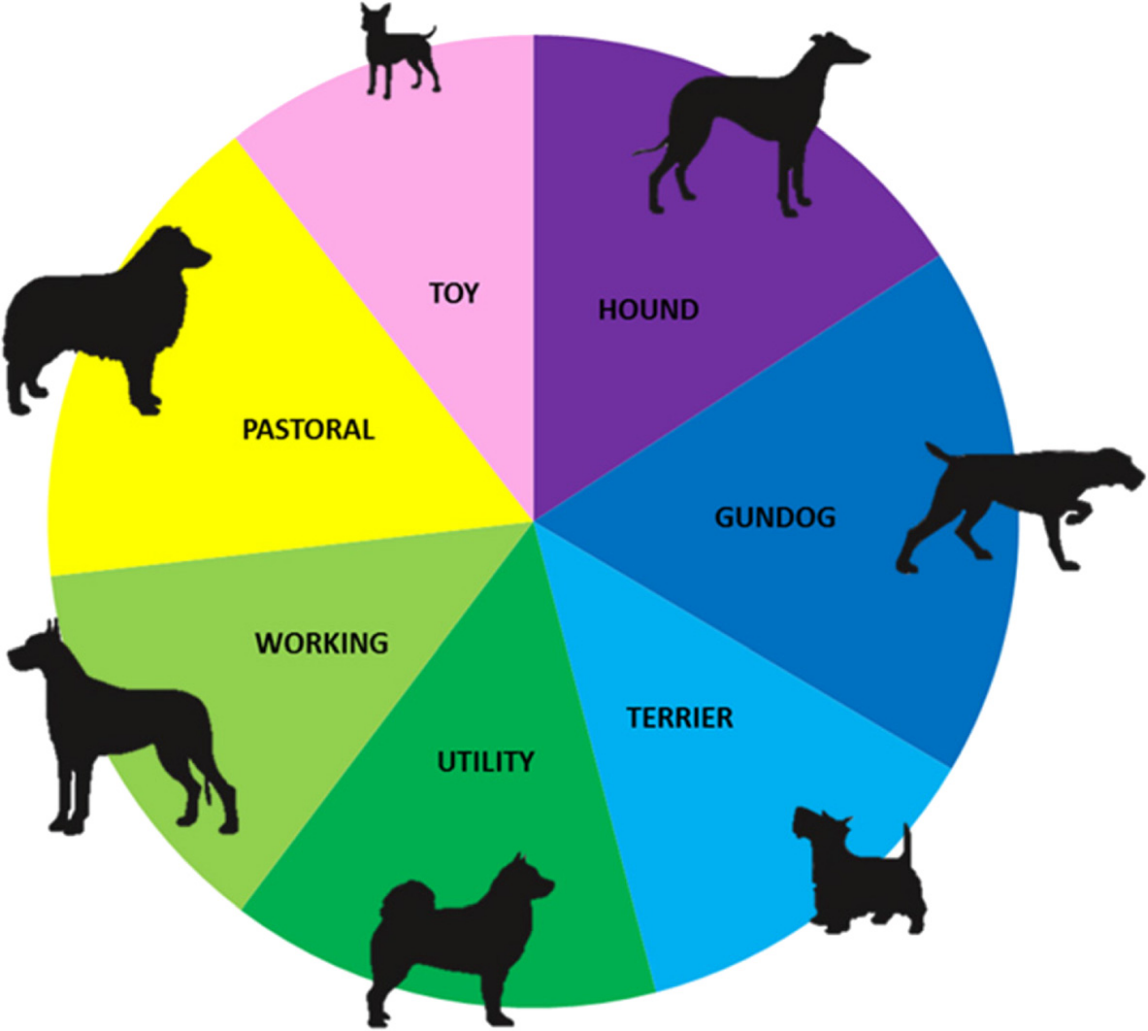
**Grouping of purebred dog breeds.** The 215 breeds recognized by the UK Kennel Club are classified into 7 groups, designated by the original function of the breed. The sizes of the sectors of the pie chart represent the number of breeds within each group category. The Hound Group includes dogs used for hunting. The Gundog Group includes dogs used for hunting game birds, scent tracking and retrieving game. The Terrier Group are dogs used to catch vermin or foxes. The Utility Group are dogs that were traditionally used for working or guarding, but today they are largely companion animals. The Working Group are dogs used for both hunting, drafting and guarding. The Pastoral Group includes dogs used for herding and guarding. The Toy Group are companion animal dogs due to their small size.

From as early as 70 AD basic morphologies and types of dogs were identified, but breeds as we recognize them today were not formalized until the 19^th^ century when dog showing and breeding during the Victorian era became increasingly popular [5,6]. With the rise in interest in purebred dogs, Kennel Clubs were founded in the United Kingdom and USA in the late 1800s to govern dog showing and breeding, register dogs and establish the first stud books [7]. The UK Kennel Club currently recognizes 215 breeds of dog and classifies them into seven groups, designated by the original function of the breed (Figure 1). The Hound Group includes dogs that were traditionally used for hunting: scent and sight hounds. The Gundog Group includes dogs used for hunting hidden game birds (Spaniels), scent tracking (Pointers and Setters) and retrieving game (Retrievers). The Terrier Group were dogs used to catch vermin or foxes. The Utility Group includes dogs traditionally used for working or guarding, but today they are mostly companion animals, whereas the Working Group were dogs used for both hunting, drafting and guarding the home. The Pastoral Group includes breeds involved in herding and guarding livestock, and the Toy Group includes dogs traditionally kept as companion animals due to their small size [8]. Last year in the UK, a total of 216,856 purebred dogs were registered with the Kennel Club and as of 2010, there were an estimated 9.4 million companion dogs in the UK [9], although accurate estimates of the total number of purebred dogs, registered dogs, and crossbred/mixed breed dogs have not been made.

**Figure 2:**
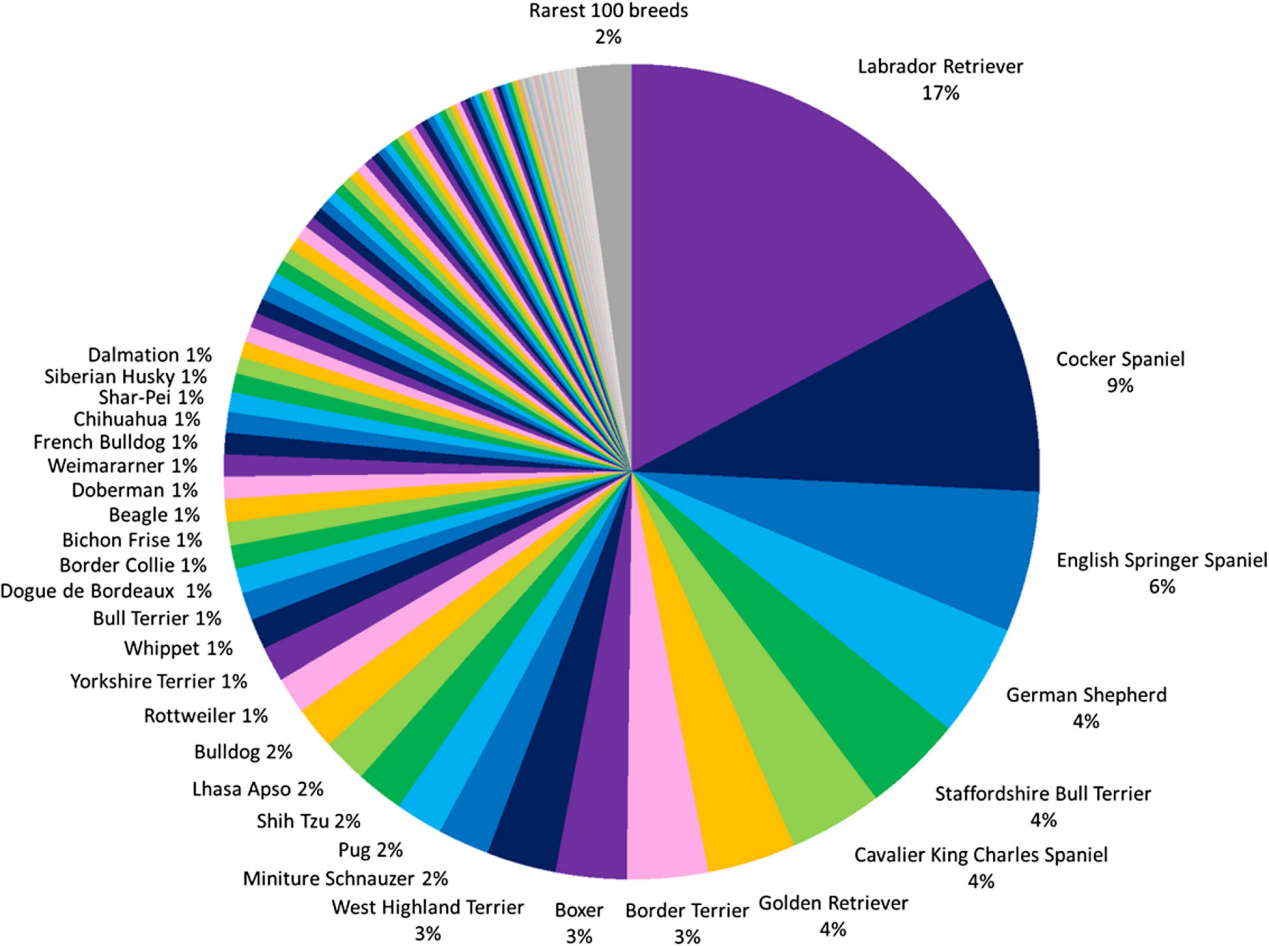
**Proportion of purebred UK Kennel Club registered dogs (2003-2013).** The top 20 most popular breeds account for 72% of total registrations, with the 100 rarest breeds accounting for 2%. Sixteen native UK breeds included in the rarest 100 are currently designated as vulnerable breed status in the UK.

The 20 most popular dog breeds account for 72% of total registrations with the UK Kennel Club, while the rarest 100 breeds account for only 2% of registrations, including 16 native UK vulnerable breeds (Figure 2). The most popular breeds are easily recognisable and the top ten dogs with the highest number of registrations with the Kennel Club in 2013 were: Labrador Retriever (35,026 registered), Cocker Spaniel (22,943), English Springer Spaniel (11,316), Pug (8,071), German Shepherd (7,954), Golden Retriever (7,117), French Bulldog (6,990), Border Terrier (6,390), Bulldog (5,769) and Staffordshire Bull Terrier (5,767).

### Canine population structure

The propagation of breed specifications and registration restrictions, such as the rule that a dog can only be registered within a breed if both its sire and dam are registered, has resulted in reproductive isolation of dogs of each breed, creating the “breed barrier” [10]. The result of dog breeds having been shaped by human preferences and kept in separate and distinct populations is that each breed is a closed breeding population with high levels of phenotypic homogeneity and receiving no further genetic admixture beyond the founding population. This has meant large genetic differences between breeds, extensive linkage disequilibrium within breeds [11,12] and widespread changes in levels of heterozygosity and haplotype structure [13]. Studies using microsatellite data have shown that nearly every breed has a different allele frequency and distribution, making them genetically identifiable and distinct from other breeds [10]. The USA purebred dog population can be divided into four main genetic clusters, or sub-populations, based on microsatellite genotypes: Asian and African ancestry dogs, Mastiff-like breeds, and Herding and Hunting dog groups [10]. Factors such as breed popularity and breeding for the propagation of specific phenotypic traits have contributed to the high degree of genetic homogeneity within individual breeds, but also to the degree of genetic heterogeneity found between different breeds [14].

### Levels of genetic diversity

The loss of genetic diversity in purebred dogs can be attributed to two major population bottleneck events: the first occurring during domestication; and the second arising from breed formation where the repeated use of popular sires, line breeding, breeding for specific phenotypic traits, and promotion of the breed barrier rule, contributed to overall loss in genetic variation [15-19]. Widespread use of a popular male to sire many litters leads to overrepresentation of that dog’s genome in the breed. As a consequence, the genetic diversity within a population is reduced, leading to a smaller effective population size. Moreover, overrepresentation of a popular sire’s genome risks the widespread dissemination of monogenetic inherited disorders by inflating the allele frequency of recessive deleterious variants carried by the sire and increasing the probability of identity by descent of undesirable alleles in his descendants [18,20,21]. Major events in history such as war and economic depression have also been the cause of significant population bottlenecks in some breeds, restricting breeding to only a few individuals. For example, in the United Kingdom during the first and second World Wars a number of breeds were reduced to 20 or fewer individuals with others disappearing completely [22].

Many breeds have passed through significant genetic bottlenecks due to a high level of inbreeding to maintain breed standards [18] which increases the level of homozygosity for detrimental alleles and is known to be a significant causative factor in the number of inherited disorders in specific breeds [22-24]. The Bouvier des Flandres is a breed which exhibits a relatively high level of homozygosity due to inbreeding and numerous inherited disorders are believed to have risen in prevalence as a result [25].

However, not all breeds exhibit a loss of genetic variability. In a recent UK study, several breeds were shown to maintain a high degree of genetic diversity, in particular the Jack Russell Terrier, which showed extensive admixture and very low levels of inbreeding [19]. The Jack Russell is not recognized as a breed by the Kennel Club in the UK, where a similar breed, the Parson Russell Terrier is recognized. This provides UK Jack Russell breeders with a broader pool of potential mates for their animals, because it avoids the registration restrictions discussed above, maximising genetic diversity within the breed [19].

The loss of genetic variation and the presence of inbreeding do not always mean an increased incidence of inherited disease and poor health. In a recent Swedish study, extensive loss of genetic variation and moderate levels of recent inbreeding did not appear to be a main cause of poor health in a number of pedigree dog breeds [26]. In another study, there was little correlation between current levels of inbreeding or reduced heterozygosity and prevalence of genetic disease [19]. One explanation for this phenomenon is that the major part of the deleterious genetic load may have accumulated before breed registration and the founding of today’s pedigrees, so that current inbreeding has not further depressed genetic variation sufficiently to result in additional genetic problems. Furthermore, deleterious recessives may have been bred out (purged) over generations of inbreeding, as has been reported for human consanguineous families [27,28]. Alternatively, this may be due to the vigilance of owners and breeders making an effort to health screen dogs regularly and avoid breeding from high risk individuals. Nonetheless, the loss of genetic variation has been associated with the unhealthy morphology and physiology of many breeds [29]. This review will look at ways in which the loss of genetic variability associated with narrow breeding goals can be mitigated by breeding strategies to improve breed health while maintaining specific breed characteristics.

## Review

### Inherited disorders

The shift towards dogs as companion rather than working animals has resulted in changes in breed characteristics with breeding being focussed towards an aesthetic rather than working or cognitive ability, and inherited disorders in pedigree dogs have been classified as being either related or unrelated to breed standards [23,24]. Conditions not relating directly to breed standards account for over 75% of all inherited disorders in pedigree dogs [24] and have been attributed to breed formation and small effective population size, the repeated use of popular sires and inbreeding. The development of the breeds has been associated with the increasing prevalence of a large number of genetic diseases [13]. However, there are more than 80 disorders that are either directly or indirectly associated with the requirements of the published breed standards which can have a detrimental impact on the dog’s health and welfare [23].

Artificial selection of dogs for specific phenotypes circumvents Darwinian natural selection. None are more dependent on human intervention than breeds of extreme skull shapes and size. Comparing historic photos of breed champions to their modern day kin, it is readily apparent that there has been selection towards exaggerated phenotypes among some breeds. The “stop” (the angle formed by the rostrum and forehead) is more acute in today’s St. Bernard and the “Roman nose” of the Bull Terrier angulates more ventrally than it did decades ago [29,30]. Perhaps the most extreme example of breed morphology is that of the English Bulldog, a breed that emerged from the bull-baiting dogs of eighteenth century Great Britain. Today’s Bulldogs, typified by their large and flat-faced head (“brachycephaly”), bowed limbs, broad chest, low-slung body, and corkscrew tail has little in common with the athletic build of their sporting ancestors. Survival of this breed is truly dependent on human intervention: because foetus head size of this breed (as well as other brachycephalic and toy breeds) is too large to pass unaided through the female’s pelvis, up to 94% of all births require Caesarean sections to deliver litters [31,32].

Artificial insemination and Caesarean deliveries are widespread practices in the world of animal husbandry. However, the difference between livestock animals, where such reproductive practices are common procedures [33], and the propagation of dog breeds, is that they are used to perpetuate aesthetic features that come at a cost. For example, brachycephalic airway syndrome (BAS) is a respiratory condition common to flat-faced breeds like the Pug, Bulldog, Boston Terrier and others of similar skull conformation [34]. The origins of this condition are complex and varied, but the correlation between head shape and BAS occurrence points to multifocal respiratory resistance – pinching of the nares, occlusion of the turbinates and extension of the soft palate into the nasopharynx. In extreme examples, the saccules and/or tonsils evert into the larynx, or the larynx becomes hypoplastic and collapses, further exacerbating the inability of the BAS sufferer to breathe. Left untreated, BAS dogs are exercise-intolerant, prone to overheating, and have increased mortality [35]. Other morbidities, particularly ocular types, are also often seen in dogs with brachycephalic skulls. Because of their shallow eye sockets, these breeds are prone to eye trauma, ulceration, and proptosis (forward displacement) (http://www.ufaw.org.uk/). When dogs with these extreme phenotypes are delivered by Caesarean section, there is no motivation for selection against this aesthetic and hence the associated morbidity and mortality will increase.

Brachycephalic breed dogs are also at increased risk for cleft lip and/or palate (CL/P), but not so much as the Spanish Pachón Navarro and the Turkish Catalburun. For both, CL/P is a breed-defining feature: folklore maintains that their “split-nose” lends these dogs superior scenting abilities needed for hunting [36,37]. Given these breeds’ scarcity and small following, it is unclear to what extent CL/P affects these animals’ quality of life.

In addition to head shape, there have also been trends which push the limits of body size. In order to achieve the petite size of toy breeds, breeding efforts have selected and consolidated genetic variation that limits growth potential through mechanisms thought to impair growth hormone and insulin-like growth factor (*IGF1*) signaling pathways [38,39].

Although not officially recognized as distinct breeds, so-called “teacup” varieties of toy dogs like the Chihuahua are in vogue; these are dogs that weigh less than a few kilograms. They suffer from numerous health conditions related to size reduction, including bone fragility and bone growth. In fact, soft spots on the heads of toy and teacup varieties of dogs are common, as cranial bone development often terminates before the fontanelles within the skull are closed. The soft spots have become an acceptable part of the breed standard in the American Kennel Club (AKC), though they are no longer mentioned in the UK Kennel Club’s breed standard (http://www.akc.org; http://www.thekennelclub.org.uk/). Giant dog breeds are also susceptible to growth-related problems thought to be linked to rapid long bone growth, in particular the crippling orthopedic condition osteochondrosis [40]. These large dog breeds are also prone to other morbidities with suspected links to bone growth, such as osteosarcoma and gastric dilation volvulus. Finally, it is widely recognized that the average lifespan of giant breed dogs is shorter than that of smaller breed dogs [29]. One long standing hypothesis is that the rapid growth rate of giant breed dogs releases more free radicals during development, causing oxidative damage and premature aging [29,41-43]. Even after adjusting for cause of death by free-radical diseases, the average lifespan of giant breeds like the Irish Wolfhound and St. Bernard was still lower than smaller breed dogs, suggesting that the factors responsible for reducing giant breed lifespan are likely to be a complex mix of morbidities linked to growth including, but not inclusively restricted to, free-radical release [29].

Clearly a relationship between dog morphologies and diseases exist [23]. However determining the “morbidity” contributions of variants that influence canine morphology is not straightforward. When there is morphological variation within a dog breed, correlating animal health to phenotype is possible, as demonstrated by a recent post-mortem study of size and morbidity using 145 autopsies of Portuguese Water Dogs [44]. In this study the authors described suggestive associations between post-mortem histological observations and *IGF1* haplotype status, the latter which is known to be highly associated with animal size [38,45]. As more causal genetic variants that underlie dog morphologies are discovered it will be important to determine whether these variants might act pleiotropically, predisposing their canine bearers to a range of diseases.

### Management of inherited disease

As early as 1963, the British Small Animal Veterinary Association identified 13 conditions of concern in pedigree dogs which resulted in several follow-on reports issued from the Council for Science and Society (1988) and more recently, the Companion Animal Welfare Council (2008) [46,47]. Currently 396 disorders have been identified in pedigree dogs that are caused or suspected to be caused by a genetic mechanism [23,24]. With the airing of *Pedigree Dogs Exposed*, a 2008 BBC documentary about the world of pedigree dog breeding and showing, the prevalence of inherited disorders and health of pedigree dogs has been widely discussed and three major reports have been produced in the UK [40,48,49]. These reports addressed the issues of inbreeding, inherited disorders, and the overall welfare implications of pedigree dog breeding, making recommendations to improve current standards. These issues are now being addressed by the Advisory Council on the Welfare Issues of Dog Breeding, the People’s Dispensary for Sick Animals (PDSA) and the Royal Society for Prevention of Cruelty to Animals (RSPCA). It is important to note, that even though a breed of dog is known to be susceptible to a certain group of inherited disorders, this does not mean that every dog of that breed will manifest all, or any, of them. Some dogs may inherit genetic variants for disorders associated with the breed, while others may inherit none. The likelihood of inherited disorders in an individual dog depends upon several factors, not limited to the accepted management practices within the breed and the practices of individual breeders themselves.

#### Breeding strategies and screening schemes

The UK Kennel Club currently registers over 6,600 assured breeders across all recognized breeds in the United Kingdom. The Assured Breeders Scheme works to promote good breeding practice to produce healthy puppies (http://www.thekennelclub.org.uk/breeding/assured-breeder-scheme/). To be an assured breeder in the Scheme, breeders must adhere to mandatory screening requirements in the choice of sires and dams. The scheme also includes additional strongly recommended (but not required) screening and advice for most breeds. Adhering to a mandatory screening protocol means a breeder must comply with any relevant breed-specific DNA disease tests and follow any British Veterinary Association/Kennel Club/International Sheep Dog Society (BVA/KC/ISDS) eye, elbow, hip dysplasia schemes and veterinary diagnostic tests to ensure that a potential sire or dam is not a carrier for a known hereditary disease.

Breeding strategies incorporating screening schemes have been highly successful in significantly reducing the prevalence of an inherited disorder and in some cases improving the overall health of the breed [50,51]. For example, patellar luxation is a common orthopedic disorder affecting dogs and while it was previously considered to primarily affect small breeds [52,53], recent studies observed increasing prevalence in medium and large breeds [54]. The Dutch Kooikerhondje, or Kooiker, is a medium Dutch gundog breed in which approximately 24% of all dogs are affected with patellar luxation [55]. The Kooiker is an old breed that dates back to the early 1600s, but then disappeared and was later re-established in 1942, registered with Dutch Kennel Club in 1971, and with the Fédération Cynologique Internationale (FCI) in 2009 [55]. The breed has been through a significant genetic bottleneck, with the current population reportedly founded by nine dams and six sires [56]. It is thought that the small number of individuals used to re-establish the breed has played a significant role in the widespread distribution of inherited disorders [56]. In 1994, a patellar luxation screening scheme for Kooiker dogs based on orthopedic examination was established in the Netherlands. From 1994 to 2009, the use of the orthopedic screening results in breeding strategies decreased the prevalence of patellar luxation in Kooiker dogs from 28% to 19% [56]. Although the prevalence of patellar luxation in Kooiker dogs has significantly decreased, it still remains at an increased incidence compared with the level in other susceptible breeds such as the Cocker and Tibetan Spaniels. Combining current screening schemes with pedigree and genotyping information could prove helpful in selective breeding programs to further reduce the prevalence of disorders with complex inheritance such as patellar luxation [55]. A recent genome-wide association analysis [57] identified nine single nucleotide polymorphisms (SNPs) in eight loci, that were associated with patellar luxation in the Flat-Coated Retriever (P < 10^-4^). Even though these data are preliminary, the results present an opportunity to utilize new genotyping information by screening potential sires and dams for the disease-associated alleles for these nine SNPs and combine this information with current orthopedic screening schemes in breeding strategies to help further reduce the prevalence of patellar luxation in this breed.

There are some screening schemes that have had no impact on significantly reducing or eliminating disease. The Cavalier King Charles Spaniel (CKCS) breed is susceptible to 25 inherited disorders, the most common of which is early-onset myxomatous mitral valve disease (MMVD) [23]. In a 2004 survey, 42.8% of all UK CKCS died due to cardiac causes and there is increasing evidence that CKCS mitral valve disease is genetic in origin, with a heritability of between 0.33 and 0.67 [58]. Although the UK Kennel Club only has one mandatory screening scheme for CKCS breeders (BVA/KC eye scheme; Additional file 1: Table S1), CKCS breed clubs in the UK voluntarily adhere to strict mitral valve disease (MVD) breeding protocols in the hope of eliminating the disease from the breed (http://www.cavalierhealth.org/mitral_valve_disease). In 2001, breeding guidelines aimed at reducing the prevalence of MMVD in CKCS were introduced in Sweden [59]. These guidelines advised that an individual should not be used for breeding until it is shown to be unaffected at four years of age, or unless both parents were unaffected at four years of age in which case the individual can be bred at two years of age (http://www.cavaliersallskapet.net/avel-och-halsa/avelsrekommendationer/). Subsequently, 131 six year old dogs that had been bred according to these guidelines were tested for heart murmurs, 56 born in 2001 (around the time the recommendations were introduced) and 75 born two years later, in 2003. In the 2001 cohort, the prevalence of heart murmurs at six years of age (in 2007) was 52% (50% for females and 54% for males) and in the 2003 cohort, the prevalence (in 2009) was 55% (44% for females and 67% for males). No significant difference was found in the prevalence of heart murmurs between 2007 and 2009 (P = 0.8) [59]. Thus in the first two years, the guidelines had no impact on reducing the prevalence or eliminating MMVD disease in CKCS. Whether longer term implementation of the guidelines will make an impact has not yet been assessed. The MVD protocol voluntarily in use by UK CKCS breeders however, advises not breeding from an individual if the parent is affected at less than five years of age, and it remains to be seen whether restricting breeding based on the disease status of the parental generation will have a positive effect in reducing the prevalence of MMVD in the UK CKCS population.

#### Estimated breeding values and genomic selection

Estimated breeding values (EBVs) are currently in use by animal breeders and recommended by the UK Kennel Club as a tool in the screening of potential sires and dams for genetic diseases which are thought to have complex inheritance or where the inheritance pattern is unknown (http://www.thekennelclub.org.uk/services/public/mateselect/ebv/Default.aspx). The EBV measures the potential of an animal to pass a specific trait to its offspring and is calculated using the animal’s phenotype (where available) and those of relatives, in conjunction with pedigree relationships. This is particularly useful for selection on complex (generally quantitative) traits, i.e. those influenced by multiple genes and environmental factors. EBVs have been utilized for livestock breeding for decades and have resulted in dramatic changes in various production traits [60]. More recently, they have also been calculated for health and welfare traits [61]. A key feature of EBVs is that they allow breeders to make breeding decisions even without phenotype information from the animal itself; for example, the primary selection on dairy traits in cattle has been carried out on sires, for which a direct phenotype cannot be measured so that the EBV relates to the productivity of the daughters. The use of EBVs has recently been introduced into dog breeding in the context of hip and elbow dysplasia, traits with complex genetic inheritance [62,63]. Currently EBVs for hip and elbow scores, which measure the propensity for hip and elbow dysplasia, are available for a variety of dog breeds in several countries, including Finland, Sweden, UK and USA (http://www.kennelliitto.fi/en/news/frequency-of-canine-hip-and-elbow-dysplasia-decreasing-in-finland; [64]; http://www.thekennelclub.org.uk/services/public/mateselect/ebv/Default.aspx; https://secure.vet.cornell.edu/bvhip/).

With genotyping and sequencing technologies becoming increasingly cost-effective, implementing genomic selection strategies –– in which genomic EBVs (gEBVs) are calculated using relationships based on genome-wide markers in place of pedigree-based information – may be the way forward in managing disorders in certain predisposed breeds on a population-wide scale. Selection based on conventional phenotype-based EBVs or gEBVs rather than on individual phenotypes is expected to substantially increase the rate of response for complex traits like hip and elbow dysplasia [62,65,66]. This three-pronged strategy, incorporating new and current screening schemes, pedigree information, and EBVs or gEBVs, could reduce the number and prevalence of inherited disorders, while at the same time genetic diversity can be managed. This is particularly important in rare breeds with a small or decreasing population size and for breeds predisposed to a high number of inherited disorders.

#### Limitations of DNA disease tests

The majority of dog breeds recognized by the Kennel Club in the UK have at least one mandatory screening scheme, but there are several breeds which have either no mandatory screening schemes or only very basic screening schemes, despite having a high number of known inherited disorders and breed specific health tests available (Additional file 1: Table S1). Examples are: the Boxer with 63 disorders, no mandatory screening scheme, four health tests available; the Golden Retriever with 58 disorders, two mandatory screening schemes, eight additional health tests available; the Labrador Retriever with 55 disorders, two mandatory screening schemes, twelve additional health tests available (Additional file 1: Table S1). It is important to note that it may be counterproductive to make all available health tests mandatory for assured breeders. This would risk alienation of breeders and withdrawal from the voluntary regulation of the assured breeder scheme.

Some breeds have health tests available even when the disease prevalence in the breed is low or non-existent. If the prevalence in the breed is high, health tests should be mandatory but, regardless of prevalence, breeding decisions should never be made on genetic testing alone. Doing a genetic test and subsequently eliminating an individual from the breeding population may not be the best strategy, as by targeting a particular allele at one genetic locus for removal from the gene pool of a particular breed, breeders may in fact increase allele frequency of genetic variants on alternative haplotypes at the same, or a different locus, that are recessively deleterious. In addition, by eliminating some animals from breeding, a reduction in the effective population size will occur, thus risking higher levels of inbreeding, potential founder effects and genetic bottlenecks. In essence, by correcting one problem there is a chance of inadvertently creating a new one. In addition, several DNA tests currently available are based on preliminary or assumed relationships between susceptibility and developing disease. Thus, important breeding decisions are being made based on this limited information. Each test also incurs a significant cost that is borne by the dog’s owner. Often tests are patented, which inhibits the opportunity to reduce their costs that free commercialisation would bring, therefore creating a limiting factor which may be detrimental to the breed long-term. Two examples of the limitations of DNA tests are discussed below.

Goniodysgenesis is a developmental abnormality of the anterior chamber of the eye and is strongly associated with an increased risk of developing primary closed angle glaucoma (PCAG) in both humans and dogs. In dogs, goniodysgenesis is detected as narrowing of the entrance to the ciliary cleft, with dysplastic pectinate ligaments that may show as sheets of undifferentiated mesenchymal tissue [67]. The outcome is an obstruction to the normal flow of aqueous humor through the trabecular meshwork, which can result in a sudden increase in intraocular pressure, quickly causing irreversible damage to the optic nerve leading to blindness [68,69]. Primary glaucoma affects a number of dog breeds although only one causative locus has been identified. Mutations in the *ADAMTS10* gene, located on chromosome 20, causes primary open angle glaucoma (POAG) in the Beagle [70] and Norwegian Elkhound [71]. Recently, a region located on canine chromosome eight was found to be associated with late onset PCAG in the Dandie Dinmont Terrier [72]. However, the causative mutation and its effect on gene expression and function have not been identified.

PCAG is an emerging cause of irreversible blindness in the Border Collie and is associated with a rise in the incidence of goniodysgenesis, which has concerned breeders. There appears to be a strong primary genetic predisposition to goniodysgenesis, since some lineages of the breed have a high number of affected animals and goniodysgenesis in these lineages is associated with increased likelihood of developing glaucoma (http://bc-glaucomadatabase.synthasite.com/). However, not all dogs affected with goniodysgenesis go on to develop glaucoma. In humans, only 10% of people diagnosed with anatomically closed angles will develop PCAG [73] and this is also observed in UK Border Collies where some individuals with goniodysgenesis never go on to develop the disease. As of 1 January 2014, the Border Collie breed was added to Schedule B of the BVA/KC/ISDS Eye Scheme, as a breed currently under investigation for goniodysgenesis/primary glaucoma (http://www.bva.co.uk/Canine-Health-Schemes/Eye-scheme/). The current recommendation from the BVA/KC is to not breed from dogs affected with goniodysgenesis if the breed is listed in the Schedule [74]. However at present, the underlying genetic architecture of goniodysgenesis and its relationship to the development of glaucoma in the Border Collie is simply not known. Until this relationship is clarified, basing breeding strategies solely on the presence of goniodysgenesis, or on a specific DNA test for goniodysgenesis, may not reduce or eliminate glaucoma from the Border Collie population, especially if glaucoma is genetically heterogeneous and/or epistatic involving several different loci in this breed.

This example highlights the importance of fully understanding the genetic basis of a condition before introducing a genetic test. For single-gene DNA tests to be fully effective in reducing or eliminating inherited disorders, the tests must not be based on any preliminary or assumed relationship, and we must understand the full biology between susceptibility and resulting development of disease. In this respect, DNA tests that directly assay disease causing mutation(s) will be most effective for disease management.

However, genetic heterogeneity within and between breeds is also a factor to consider, as the utility of DNA testing may be breed-specific, or worse yet, sub-population specific as exemplified by testing for progressive retinal atrophy (PRA). PRA encompasses several different types of inherited retinal diseases characterized by retinal degeneration and progressive loss of vision. Two main general types of PRA are recognized in the UK: those involving dysplasia/degeneration of the rods and those affecting the cones [74]. PRA is one of the most commonly inherited disorders in pedigree dogs and although the various forms have been documented in over 100 breeds and exhibit similar clinical signs, the etiology, age of onset, and severity of the disease differ significantly between breeds [75]. Most forms of PRA are known or highly suspected to be inherited by an autosomal recessive mode of inheritance, but some breeds have been found to carry the disorder as an X-linked or autosomal dominant trait (Additional file 2: Table S2). Eighteen genes and 24 causative mutations have been associated with PRA and there is increasing evidence that multiple different forms of the disease segregate in more than one affected breed, and even within an affected individual [76]. The BVA/KC Eye Scheme, BVA/KC/ISDS Eye Scheme and/or various breed specific PRA DNA tests are currently in use by assured breeders registered by the UK Kennel Club as mandatory screening schemes for a large proportion of breeds (Additional file 1: Table S1). The problem with this approach is that PRA is heterogeneous both within and between breeds [75]. This means that two or more distinct mutations may be present in a single breed, making PRA DNA testing between potential sires and dams misleading, as individuals may possess a mutation not targeted by the specific DNA test recommended. This means that veterinarians, scientists, breeders and owners should avoid making any assumptions about the possible genetic cause of PRA in any dog, and all available DNA tests for the various forms of PRA should be used to assess all clinical cases, regardless of the recommended breed specific test [75]. In these cases, using all possible diagnostic and genetic tests available (and ultimately making use of genome-wide genotyping) along with pedigree information may be the only way to make informed breeding decisions that will benefit the breed and help to steer away from breeding potential carriers of multiple PRA-alleles.

#### Introgressing the normal allele

When a breed population carries a high frequency for a known detrimental allele at a specific genetic locus, it may be possible to crossbreed with an animal free of the detrimental allele and then backcross to the original lineage, monitoring the presence of the desired allele using genetic testing. Purebred Dalmatian dogs are all homozygous for a mutation in *SLC2A9*, a gene that encodes a membrane protein involved in uric acid transport [77]. This mutation appears to be closely linked to a locus controlling coloured spot size in the Dalmatian coat and probably rose to high frequency during selection for a distinctive spotting pattern in achieving breed standard. Because of this mutation, Dalmatians are susceptible to hyperuricosuria and formation of urinary calculi that can obstruct the urinary tract (a life threating complication) and which may require surgical removal.

**Figure 3:**
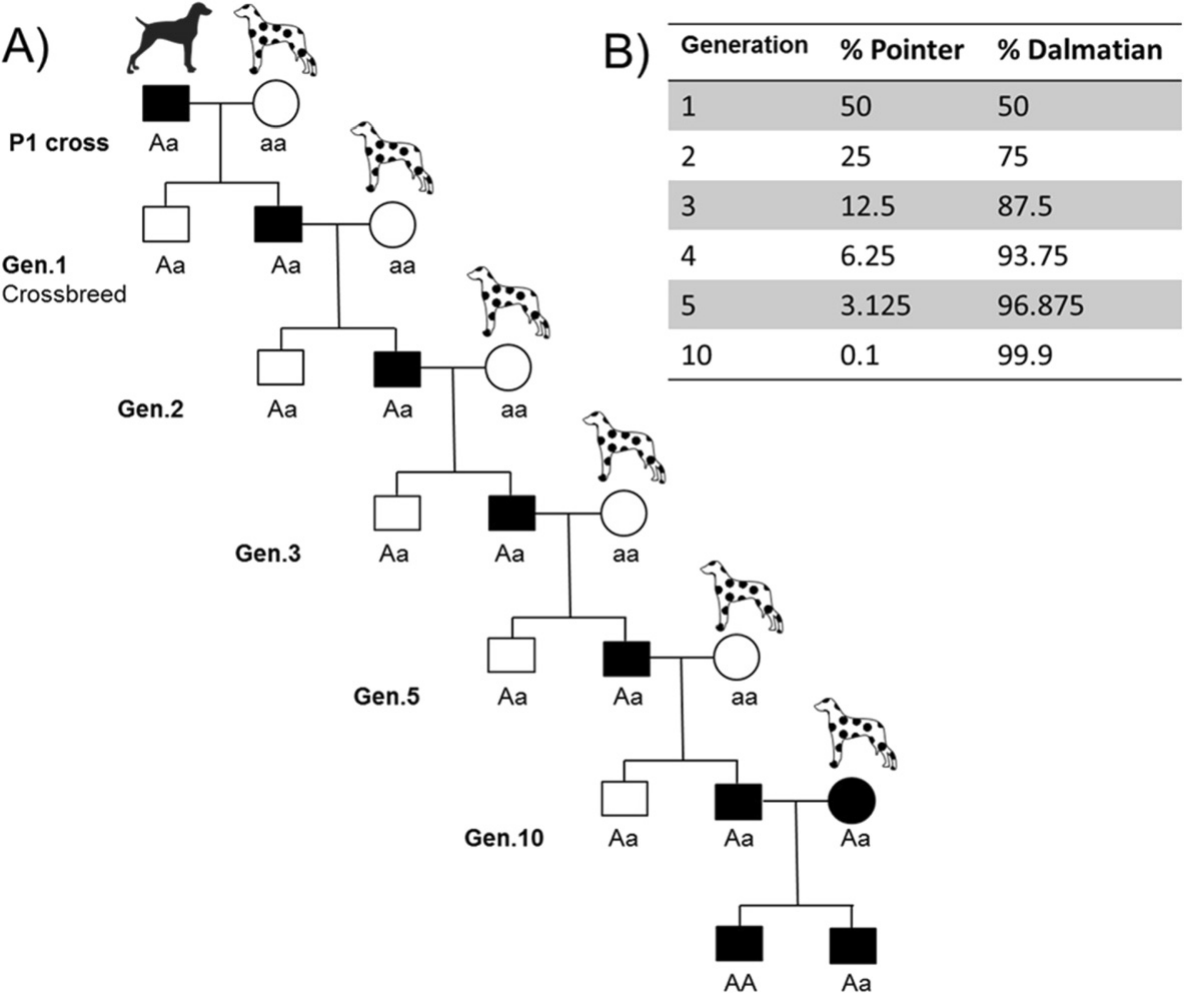
**Replacing a detrimental allele. (A)** A purebred dog from a breed carrying the dominant functional allele is crossed to a purebred dog from a breed homozygous for an inactivating mutation (P1 cross). Progeny of this cross carrying the normal allele are selected and crossed to purebred animals from the breed of interest. In each generation, those carrying the normal allele are chosen for breeding to purebred animals. Eventually it would be possible to mate two animals that are both heterozygous for the desired allele and produce homozygous progeny, with very little change in genetic composition **(B)** After five generations of backcrossing to the desired breed, the proportion of the genotype contributed by this breed is 96.9%, while by ten generations it is 99.9%.

To correct this problem, a Dalmatian was outcrossed with a Pointer homozygous for a functional allele at *SLC2A9* and then successive backcrosses to the Dalmatian line were performed [78]. As shown in Figure S1 of Bannasch et al. [78], this resulted in dogs that were heterozygous for the mutation (and therefore excreted only low levels of uric acid) but of predominantly Dalmatian genetic background. The backcross dogs descended from the original Dalmatian x Pointer cross have been registered with the American Kennel Club in the United States and meet the breed standard. As seen in Figure 3, after five generations of backcrossing to the original breed, the genetic contribution is 96.9% from this breed while by ten generations it is 99.9%. Although successful, the Dalmatian x Pointer cross used a single F1 individual to backcross to the Dalmatian, and thus all descendants segregate copies of the wild-type allele which are identical by descent. This breeding strategy had the potential to have negative effects, as new deleterious alleles in linkage disequilibrium with the functional *SLC2A9* allele could have been introduced and retained causing a problem in homozygotes for the wild type allele, although this does not seem to have been the case. Crossbreeding with several individuals (or several different breeds) followed by backcrossing would still produce an animal that is free of the disease while still meeting the breed standards and being almost genetically indistinguishable to pure bred Dalmatians. In 2010, the first Dalmatian x Pointer lineage dog was registered by the UK Kennel Club (Ch. Fiacre’s First and Foremost). Even though she was 13 generations away from the Dalmatian x Pointer cross, some Dalmatian breed clubs objected to the acceptance of the registration [79]. During Crufts 2011, the first year Ch. Fiacre’s First and Foremost was invited to compete, a few breeders openly criticized the legitimacy of her pedigree status and sparked several newspaper and media articles surrounding the issue [80]. This illustrates that even if a governing authority makes changes to breed standards that it feels will benefit the breed, the support of the public, breeders and/or breed clubs is paramount in accepting these changes and helping make them successful.

### The future of the pedigree dog

Insurance companies providing health insurance for purebred dogs record how often dogs of each breed use their insurance and for what purpose, and this determines the premiums paid, so for many owners, insuring a particular breed of pedigree dog may mean paying very high premiums in comparison with a crossbreed. In the UK, the cost of insuring a small pedigree dog for standard or basic coverage can be of the order of £300 (approx. $500 US) per year, and will significantly increase for large breeds and breeds known to be predisposed to poor health. In the UK only 42% of all dogs, whether purebred or not, are insured [9] and whilst there are charities like the PDSA which are dedicated to helping with pet health costs, these too are increasingly rejecting purebred dogs. PDSA policy currently dictates that only one pedigree dog may be registered per household (https://www.pdsa.org.uk/pdsa-vet-care/eligibility). A recent study found that 48% of companion animal veterinarians were advising clients against purchasing a pedigree dog breed due to inherited disorders [81]. It has been suggested that for the maximum wellbeing of future generations, we should abandon the most predisposed breeds (those with the poorest health record and highest number of inherited disorders) while loosening the genetic barriers between the remaining breeds to promote genetic variability [82]. This strategy to preserve the majority of breeds, but not all of them, is something that would ensure the long-term survival of dog breeds in general, but it would mean allowing certain predisposed breeds to be lost and also abandoning the strict characteristics of specific breeds by interbreeding them.

A more realistic solution for the management of the most predisposed breeds would be to cross with several individuals from a closely-related breed to reintroduce genetic variation and combine this strategy with breeding schemes to breed away from the most susceptible individuals to ensure more genetically diverse future generations. As with the Dalmatian x Pointer crossbred dogs discussed above, this would allow for genetic admixture but still work to maintain the standard characteristics of the breed. For breeds without a small or decreasing population size, it would be beneficial for Kennel Clubs worldwide to impose limitations on the number of offspring per stud, thus reducing the popular sire effect and promoting increased genetic variability on a population-wide scale. Such restrictions on sires are already in place for the German Shepherd Dog [83]. For rare breeds and those with small or decreasing population sizes, efforts should be made to mate sires and dams that are as unrelated as possible by considering kinship coefficients calculated from pedigree information and also incorporating genotyping data as a standard genetic test for every dog registered, in order to monitor inbreeding on an individual level. In the UK, projects like Dogslife™ (http://www.dogslife.ac.uk), which compiles health and life history information via an online database and tracks individual dogs in real time, could be used as a model for the management of rare breeds and those with small or decreasing population size.

#### Designer dogs

A new movement of creating what has been termed “designer dogs” has also been slowly gaining a foothold and these crossbreeds are becoming increasingly popular among the public through combinations such as the Labradoodle (Labrador Retriever + Standard Poodle), Puggle (Pug + Beagle), Cockerpoo (Cocker Spaniel + Miniature Poodle), and the Utonagen, which is being bred in order to achieve a breed of dog that looks like a wolf (http://www.theutonagansociety.com/). While initially each dog was a first generation crossbreed, there have been attempts to fix the characteristics of the crossbreed in a pure breeding line and have this registered as a distinct breed. There is a common misconception that because the designer breeds originated from founders of distinct breeds, they will automatically be healthier and less prone to inherited disorders. Undoubtedly outcrossing will increase heterozygosity and reduce the frequency of disease-causing alleles in a breed. When choosing animals for this initial cross, it would be important to maximise genetic variability and avoid lineages known to have a high prevalence of genetic diseases. For example, the Labrador Retriever and Standard Poodle are both highly susceptible to a number of shared inherited disorders such as hip dysplasia, and various eye and joint diseases. Careful consideration of lineages and thorough diagnostic screening and genetic testing of both sire and dam must be performed to ensure healthy puppies in future generations. The hybrid vigour generated by this initial outcross would deteriorate beyond the first (F1) cross, and it would be critical that subsequent selection of animals for the “designer dog” phenotype was carefully monitored to maximise genetic diversity and avoid known inherited conditions. Selectively breeding based on aesthetics and specific guidelines can have a detrimental impact on the dog’s wellbeing and overall health, particularly if it results in inbreeding or genetic bottlenecks [23]. Thus, creating new breeds for appearances may not be in the best interest of canine welfare, given our historical experience. Such schemes should take advantage of modern genetic technologies to ensure that known genetic variants are excluded and genetic diversity is maintained in their new lineages.

**Figure 4:**
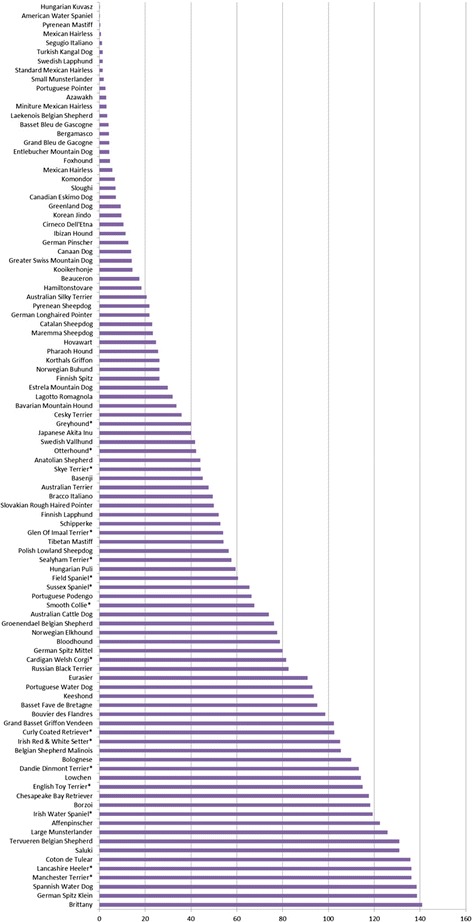
**Rarest 100 dog breeds in the UK.** Average number of UK Kennel Club registered purebred dogs amongst the 100 rarest dog breeds in the UK over the past 10 years (2004-2013). More than 30 rare breeds are registering less than 20 dogs per year. Sixteen native UK breeds are recognized as being vulnerable in the UK by the Kennel Club and are registering on average between 40 and 136 dogs per year (denoted by *asterisk).

There are already several dog breeds at a dangerously low population level in the UK. The rarest 100 breeds account for only 2% of all breed registrations with the UK Kennel Club. They include 16 native UK breeds listed with a vulnerable breed status (Figure 4). Many of these breeds are unfashionable, need additional care of the coat, or have exercise requirements not compatible with modern life and thus are at record low numbers, registering on average between 40 and 136 individuals per year (Figure 4). Some of the top UK native vulnerable breeds include: Otterhound, Skye Terrier, Glen Of Imaal Terrier, Sealyham Terrier, Field Spaniel, Sussex Spaniel, Smooth Collie, Irish Red & White Setter, Dandie Dinmont Terrier and Norwich Terrier. One can argue that instead of trying to make a new designer dog breed based on an arbitrarily chosen aesthetic, we should focus on ensuring the future of our native established breeds, in addition to putting efforts into significantly reducing and eliminating the inherited disorders already present in purebred dogs.

## Conclusion

In this review we provide an overview of the challenges facing breeders of pedigree dog breeds in combating inherited disease. Incentive programs, free diagnostic screening, and genetic testing days are already being implemented by breed clubs to help identify and diagnose inherited disorders in susceptible dog breeds, but public awareness of the importance of testing needs to be much improved. Testing and screening programs are vital to understanding both the prevalence and susceptibility to developing disease and creating breeding strategies with the aim of significantly reducing inherited disorders. DNA tests for disease causing mutation(s) will be most informative and effective for disease management but must be combined with current screening schemes, pedigree information, and if possible genomic selection, to maximize the impact in significantly reducing the number of inherited disorders and improving overall health in pedigree dogs. Recognition of the benefits of crossbreeding, acceptance for registration of dogs with a distant ancestor of another breed, and offspring limits imposed on stud dogs in Kennel Clubs worldwide would improve breed health without compromising many breed standards. Public awareness, education, and most importantly the support of breeders and/or breed clubs are significant factors in making these changes successful and common practice.

## Additional files

Additional file 1: Table S1. Inherited disorders in pedigree dogs. Additional file 2: Table S2. Inherited disorders in pedigree dogs.
