# Supplementary material for: The challenges of pedigree dog health: approaches to combating inherited disease
Source: Canine Genet Epidemiol. 2015 Feb 11;2:3. doi: 10.1186/s40575-015-0014-9 (PMC4579364; doi:10.1186/s40575-015-0014-9)
Supplement: Additional file 1: Table S1. — Inherited disorders in pedigree dogs. [file 40575_2015_14_MOESM1_ESM.docx]

**Table S1.** Inherited disorders in pedigree dogs.

| **UK Kennel Club**  **Registered Breed** | **Group** | **Number of total disorders** | **Reference Source** | **Popularity Rank** | **Average Lifespan**  **(UK Kennel Club)** | **No. of UK Kennel Club Registered Breeders** | **Breed Specific Health Tests Avaliable^+^**  **(DNA tests and veterinary diagnostics)** | **UK Kennel Club Breeders Mandatory Screening Scheme (sires and dams)** | **Strongly Recommended Screening Scheme and/or advice (sires and dams)  (UK Kennel Club and Breed Club)** | **Vulnerable**  **Breed Status** |
| --- | --- | --- | --- | --- | --- | --- | --- | --- | --- | --- |
| German Shepherd | Pastoral | 77 | 1 | 4 | >10 | 185 | ES, EDS, HDS, AF, DM, DFM, HUU, MPSVII, MDR1, RCND, haemophilia testing for males | HDS | NTPL1, EDS, ES, haemophilia testing for males, no stud dog <18 months to be used | N |
| Boxer | Working | 63 | 1 | 9 | >10 | 128 | HDS, ARVC, DM, heart testing (aortic stenosis) | No mandatory screening schemes | HDS, heart testing (aortic stenosis) | N |
| Golden Retriever | Gundog | 58 | 1 | 7 | >10 | 368 | ES, EDS, HDS, cord1-PRA, ICT-A, MD, pcrd-PRA, rcd2-PRA, DM, SAN | HDS, ES | EDS, NTPL1, NTPL2, PRA, PRA 1& 2 (additional test), ICT-A (additonal DNA test) | N |
| Miniture Poodle | Utility | 58 | 1 | 48 | >12 | 31 | ES, pcrd-PRA, vWD, DM, MPS | ES | pcrd-PRA (DNA test) | N |
| English Springer Spaniel | Gundog | 57 | 1 | 3 | >10 | 99 | ES (including Gonioscopy), HDS, Fuco, IRIDA, cord1-PRA | ES (including Gonioscopy), Fuco (DNA test), cord-1-PRA (DNA test) | HDS, PFK (DNA test), NTPL1, NTPL2 | N |
| Labrador Retriever | Gundog | 55 | 1 | 1 | >10 | 661 | ES, EDS, HDS, DFM, OSD, CU, DM, EIC, HBIX, HNP, CNM, NPY, pcrd-PRA, PKD | HDS, ES | EDS, pcrd-PRA (DNA test), CNM (DNA test), EIC (DNA test) | N |
| Doberman | Working | 53 | 1 | 24 | >10 | 48 | ES, HDS, DCM, NPY, vWD | HDS, vWD (DNA test) | ES | N |
| Cocker Spaniel | Gundog | 51 | 1 | 2 | >10 | 205 | HDS, ES, ES (Gonioscopy only), FN, PFK, pcrd-PRA, DM, IRIDA | ES, pcrd-PRA (DNA test), FN (DNA test) | HDS, ES (Gonioscopy only) | N |
| Toy Poodle | Utility | 51 | 1 | 33 | >12 | 31 | ES, pcrd-PRA, vWD, DM, MPS | ES | pcrd-PRA (DNA test) | N |
| Dachshund (Smooth/Long/Wire) | Hound | 50 | 1 | 50 | >12 | 250 | ES, LD, cord1-PRA, cord2-PRA, NHPH4, MPS, NCL, MPSIIIA, PKD | cord1-PRA (DNA test) | LD (DNA test: Miniture Wire Haired) | N |
| Great Dane | Working | 50 | 1 | 32 | <10 | 35 | HDS, heart testing (cardiomyopathy) | No mandatory screening schemes | NTPL1, HDS, heart testing (cardiomyopathy) | N |
| Beagle | Hound | 45 | 1 | 23 | >10 | 103 | FVII, MLS, NCCD, POAG, PKD, CM | MLS (DNA test) | NCCD (DNA test) | N |
| Miniture Schnauzer | Utility | 45 | 1 | 11 | >10 | 205 | ES, MPS, MYO, PRA | ES | No recommended screening scheme and/or advice | N |
| Irish Setter | Gundog | 44 | 1 | 43 | >10 | 48 | ES, HDS, CLAD, rcd1-PRA, rcd4-PRA, DM, NCL | HDS, CLAD (DNA test), rcd1- PRA (DNA test) | ES, NTPL1, rcd4-PRA (additional DNA test) | N |
| Bulldog | Utility | 42 | 1 | 15 | <10 | 169 | CMR, HUU, DM | No mandatory screening schemes | Health certificate for breeding stock, HUU (additional DNA test) | N |
| Basset Hound | Hound | 41 | 1 | 42 | >10 | 19 | ES (Gonioscopy only), EDS, TPA, SCID | ES (Gonioscopy only) | EDS | N |
| Standard Poodle | Utility | 41 | 1 | 46 | >10 | 41 | ES, HDS, DM, NE, SAT, pcrd-PRA, vWD | HDS, ES | Sebaceous adenitis, vWD (additional DNA test) | N |
| Shar-Pei | Utility | 38 | 1 | 28 | >10 | 33 | No health tests relevant to this breed | No screening schemes relevant to this breed | No recommended screening scheme and/or advice | N |
| Rough Collie | Pastoral | 37 | 1 | 44 | >10 | 19 | ES, HDS, CCN, CEA/CH, MDR1, rcd2-PRA, NCL | HDS, ES | CEA/CH (DNA test), MDR1 (DNA test), rcd2-PRA (additional DNA test) | N |
| Dalmatian | Utility | 36 | 1 | 30 | >10 | 58 | HDS, BAER, HUU | No mandatory screening schemes | HDS, BAER testing, NTPL1, NTPL2, NTPL4 | N |
| West Highland White Terrier | Terrier | 35 | 1 | 10 | >10 | 101 | GCL, PKD | No mandatory screening schemes | No recommended screening scheme and/or advice | N |
| Shetland Sheepdog | Pastoral | 34 | 1 | 34 | >10 | 10 | ES, HDS, CEA/CH, DM, MDR1, vWD | ES | CEA/CH (DNA test), HDS, MDR1 (additional DNA test), vWD (additional DNA test) | N |
| Chihuahua (Long/Smooth) | Toy | 33 | 1 | 27 | >12 | 139 | PKD | No mandatory screening schemes | No recommended screening scheme and/or advice | N |
| Pug | Toy | 33 | 1 | 12 | >10 | 172 | PDE, PKD, PLL, hemivertebrae | No mandatory screening schemes | Hemivertebrae checking | N |
| German Shorthaired Pointer | Gundog | 32 | 1 | 36 | >10 | 43 | HDS | HDS | NTPL1, NTPL4 | N |
| Rottweiler | Working | 32 | 1 | 16 | >10 | 81 | ES, EDS, HDS | HDS | NTPL1, NTPL2, NTPL5, ES, EDS | N |
| Weimararner | Gundog | 31 | 1 | 25 | >10 | 68 | HDS, HUU | HDS | NTPL1, NTPL2 | N |
| Boston Terrier | Utility | 31 | 4, 33, 64, 54, 126-132, 187, 196, 403, 423, 431, 432, 442, 447, 449, 458, 462-464, 466-471, 491 | 56 | >10 | 43 | ES, HC-HSF4 | ES, HC-HSF4 (DNA test) | No recommended screening scheme and/or advice | N |
| Chow Chow | Utility | 31 | 27, 30, 54, 64 122, 152-164, 187, 250, 423, 431, 442, 452, 462, 478-483, 491 | 71 | >10 | 22 | HDS, DM | No mandatory screening schemes | HDS | N |
| American Cocker Spaniel | Gundog | 27 | 30, 33, 55, 64, 99, 128, 158, 187, 200, 250, 264, 265, 297, 403, 408-417, 423, 432, 446, 450, 458, 488, 524-526 | 70 | >10 | 19 | ES (including Gonioscopy), IRIDA, PFK, pcrd-PRA | ES (including Gonioscopy) | prcd-PRA (DNA test) | N |
| Yorkshire Terrier | Toy | 26 | 1 | 17 | >12 | 55 | PLL, pcrd-PRA | No mandatory screening schemes | No recommended screening scheme and/or advice | N |
| Akita | Utility | 26 | 1 | 41 | >10 | 10 | ES, HDS, DM | HDS | ES | N |
| Shih Tzu | Utility | 26 | 1 | 13 | >10 | 106 | No health tests relevant to this breed | No screening schemes relevant to this breed | No recommended screening scheme and/or advice | N |
| Border Collie | Pastoral | 25 | 1 | 21 | >10 | 50 | ES, ES (Gonioscopy only), HDS, CCN, CEA/CH, MDR1, NCL, TNS, CM, DM | HDS, ES | CEA/CH (DNA test), CL (DNA test), TNS (DNA test), ES (Gonioscopy only) | N |
| Cairn Terrier | Terrier | 25 | 1 | 31 | >10 | 35 | GCL, PKD, bile acid testing (puppies) | No mandatory screening schemes | ES, bile acid testing of puppies | N |
| Cavalier King Charles Spaniel | Toy | 25 | 1 | 6 | >12 | 97 | ES, CM/SM, CC/DE, EF, MD, PFK, DM, mitral valve dysplasia | ES | CM/SM, EF (DNA test), CC/DE (DNA test), heart testing (mitral valve dysplasia) | N |
| Bernese Mountain Dog | Working | 25 | 30, 47, 64, 105-118, 111, 163, 304, 357, 375, 423, 442, 444-457, 462, 473 | 57 | <10 | 41 | EDS, HDS, vWD, DM | HDS, EDS | NTPL1 | N |
| Old English Sheepdog | Pastoral | 24 | 54, 64, 250, 255, 289, 319-329, 423, 431, 432, 442, 443, 449, 459, 481, 512, 513 | 63 | >10 | 18 | ES, HDS, MDR1, PCD | HDS, ES | PCD (DNA test) | N |
| Scottish Terrier | Terrier | 24 | 1 | 49 | >10 | 31 | vWD | No mandatory screening schemes | No recommended screening scheme and/or advice | N |
| Lhasa Apso | Utility | 24 | 1 | 14 | >12 | 74 | ES, HBIX | ES | No recommended screening scheme and/or advice | N |
| Greyhound* | Hound | 23 | 30, 33, 54, 229-242, 403, 423, 431, 442, 454, 462 | 161 | >10 | 5 | GN | No mandatory screening schemes | GN (DNA test) avaliable | Y |
| Pekingese | Toy | 23 | 64, 88, 187, 193, 198, 265, 289, 293, 335-338, 431, 432, 458, 449, 504, 514-517 | 66 | >12 | 16 | No health tests relevant to this breed | No screening schemes relevant to this breed | No recommended screening scheme and/or advice | N |
| Alaskan Malamute | Working | 23 | 1 | 45 | >10 | 47 | ES, HDS, DM, CD, AMP | HDS, ES | NTPL1, NTPL2, bitches <3rd season not to be mated | N |
| St Bernard | Working | 22 | 30, 54, 64, 145, 163, 187, 215, 255, 265, 304, 351, 371-375, 423, 444, 481, 505, 522 | 60 | <10 | 15 | HDS | HDS | NTPL1 | N |
| Newfoundland | Working | 21 | 64, 92, 255, 265, 287, 288, 300-309, 423, 433, 462, 497, 508, 509 | 47 | <10 | 38 | EDS, HDS, CU, TPA, heart testing | HDS, CU (DNA test) | NTPL1, EDS, heart testing | N |
| Samoyed | Pastoral | 21 | 54, 64, 96, 162, 187, 206, 250, 252, 265, 381-387, 452, 462, 423, 431, 432, 478, 480, 481, 536 | 80 | >10 | 20 | ES, HDS, HN, XL-PRA, OSD | HDS | ES | N |
| Australian Shepherd | Pastoral | 20 | 30, 54, 55, 60-70, 71-75, 211, 199, 250, 423, 433, 439, 454, 475 | 105 | >10 | 16 | ES, EDS, HDS, CCN, CM, CEA/CH, CD, DM, HC-HSF4, HUU, MDR1, CMR, NCL, pcrd-PRA, Pelger-Huet Anomally | HDS, ES, HC-HSF4 (DNA test) | EDS, CEA/CH (DNA test), MDR1 (DNA test), prcd-PRA (DNA test), CM (DNA test), blood test for Pelger-Huet anomally | N |
| Bullmastiff | Working | 20 | 1 | 39 | >10 | 38 | HDS, CMR, PRA, PRA-D | No mandatory screening schemes | HDS | N |
| Irish Wolfhound | Hound | 17 | 30, 45, 47, 64, 215, 237, 250, 255-257, 288, 327, 423, 431 | 72 | <10 | 12 | ES, SD, heart testing (ECG, ultrasound), liver shunt testing (puppies) | No mandatory screening schemes | ES, NTPL1, bitches >6yrs not to produce a litter, NTPL2, heart testing scheme, liver shunt testing all puppies | N |
| Pyrenean Mountain Dog | Pastoral | 17 | 64, 145, 219-226, 362, 423, 431, 496 | 100 | <10 | 9 | HDS, CMR, GT | HDS | No recommended screening scheme and/or advice | N |
| Bull Terrier | Terrier | 17 | 1 | 19 | >10 | 63 | BAER, HBIX, BTPKD, heart and kidney testing | No mandatory screening schemes | BAER testing, heart and kidney testing | N |
| Wire Fox Terrier | Terrier | 17 | 30, 64, 184, 185, 187-190, 250, 423, 431, 432, 438, 496-498 | 59 | >10 | 28 | DM, PLL | No mandatory screening schemes | No recommended screening scheme and/or advice | N |
| Bichon Frise | Toy | 17 | 1 | 22 | >12 | 46 | ES, DM | ES | No recommended screening scheme and/or advice | N |
| Maltese | Toy | 17 | 30, 44, 54, 64, 171, 187, 199,220, 265, 289-293, 327, 357, 403, 423, 432, 465, 491 | 52 | >12 | 31 | GSDI | No mandatory screening schemes | No recommended screening scheme and/or advice | N |
| Mastiff | Working | 17 | 47, 64, 74, 158, 172, 295, 298, 299, 423, 481, 484-489 | 84 | <10 | 8 | EDS, HDS, HUU, CMR, PRA, PRA-D | HDS | NTPL1, EDS, Bitches >6yrs not to produce a litter | N |
| Hungarian Vizsla | Gundog | 16 | 1 | 40 | >10 | 85 | HDS | HDS | NTPL1, NTPL2 | N |
| Australian Cattle Dog | Pastoral | 16 | 40-46, 47-49, 50-59, 163, 187, 327, 432, 433, 445, 446, 448 | 140 | >10 | 2 | ES, EDS, HDS, BAER, DM, MDR1, PLL, pcrd-PRA, rcd4-PRA, CU | pcrd-PRA (DNA test) | HDS, EDS, ES, BAER testing | N |
| Border Terrier | Terrier | 16 | 1 | 8 | >12 | 186 | No health tests relevant to this breed | No screening schemes relevant to this breed | No recommended screening scheme and/or advice | N |
| Pomeranian | Toy | 15 | 30, 64, 145, 163, 176, 289, 351-357, 423, 447, 449, 480 | 51 | >12 | 27 | HUU, VDD | No mandatory screening schemes | No recommended screening scheme and/or advice | N |
| Siberian Husky | Working | 15 | 1 | 29 | >10 | 22 | ES, ES (including Gonioscopy), HDS, GM1, XL-PRA | HDS, ES (including Gonioscopy) | Eye testing | N |
| Brittany | Gundog | 14 | 30, 64, 140, 141-145, 163, 187, 265, 403, 423, 431, 439, 440 | 108 | >10 | 6 | HDS | HDS | No recommended screening scheme and/or advice | N |
| Chesapeake Bay Retriever | Gundog | 14 | 30, 55, 64, 250, 343, 364-367, 423, 431, 432, 507 | 120 | >10 | 10 | ES, HDS, DM, EIC, pcrd-PRA, ED | HDS, ES | prcd-PRA (DNA test), DM (DNA test) | N |
| Rhodesian Ridgeback | Hound | 14 | 1 | 38 | >10 | 56 | HDS, DM, HBIX, dermoid sinus check (puppies) | HDS | Dermoid sinus check (puppies), NTPL1, NTPL2, NTPL3 | N |
| Airedale Terrier | Terrier | 14 | 26-30, 33, 64, 122, 176, 249, 352, 423, 431, 442, 444, 505 | 53 | >10 | 43 | HDS, HBIX, FVII | HDS | NTPL1, bitches >6yrs not to produce a litter | N |
| Keeshond | Utility | 14 | 64, 261-264, 266, 380, 423, 462, 480, 500-503 | 131 | >10 | 14 | PHPT | PHPT (DNA test) | No recommended screening scheme and/or advice | N |
| Giant Schnauzer | Working | 14 | 64, 145, 206-212, 217, 247, 250, 352, 403, 423, 472, 499 | 87 | >10 | 24 | ES, ES (litter screening), HDS, CM, FVII, HUU, pcrd-PRA | ES | HDS, ES (litter screening) | N |
| Pointer | Gundog | 13 | 54, 96, 145, 176, 347-350, 423, 429, 462, 518-520 | 54 | >10 | 55 | vWD, CD | No mandatory screening schemes | No recommended screening scheme and/or advice | N |
| Pembroke Welsh Corgi | Pastoral | 13 | 30, 309, 339-343, 367, 423, 432, 476, 477 | 67 | >10 | 25 | DM, EIC, SCID, vWD | No mandatory screening schemes | No recommended screening scheme and/or advice | N |
| Miniature Pinscher | Toy | 13 | 54, 64, 163, 203, 345, 346, 403, 423, 430, 432, 433, 449, 493 | 78 | >12 | 18 | MPS | No mandatory screening schemes | No recommended screening scheme and/or advice | N |
| French Bulldog | Utility | 13 | 54, 192-200, 423 | 26 | >10 | 138 | ES, HC-HSF4, HUU | No mandatory screening schemes | ES (annual test), HC-HSF4 (DNA test) | N |
| Tibetan Terrier | Utility | 13 | 1 | 37 | >10 | 74 | ES, HDS, NCL, PLL, rcd4-PRA, PRA | ES, HDS, PLL (DNA test), NCL (DNA test) | rcd4-PRA (DNA test), rcd3-PRA | N |
| English Setter | Gundog | 12 | 35, 54, 64, 122, 134, 163, 173-177, 423, 431, 438, 490 | 74 | >10 | 38 | HDS, NCL, rcd4-PRA | HDS | No recommended screening scheme and/or advice | N |
| Norwegian Elkhound | Hound | 12 | 64, 187, 250, 265, 313-317, 403, 423, 431, 511 | 138 | >10 | 11 | ES, HDS, pcrd-PRA, kidney testing | HDS, prcd-PRA (DNA test) | Kidney test, ES | N |
| Smooth Fox Terrier* | Terrier | 12 | 95, 184-189, 191, 250, 403, 423, 431, 432, 437, 497 | 103 | >10 | 9 | No health tests relevant to this breed | No screening schemes relevant to this breed | No recommended screening scheme and/or advice | Y |
| Bouvier des Flandres | Working | 13 | 4, 64, 133-138, 163, 187, 224, 403, 423, 443, 473, 534 | 129 | >10 | 7 | No health tests relevant to this breed | No screening schemes relevant to this breed | No recommended screening scheme and/or advice | N |
| Bloodhound | Hound | 11 | 47, 64, 121, 123, 212, 215, 423, 431, 458, 460 | 137 | <10 | 9 | No health tests relevant to this breed | No screening schemes relevant to this breed | No recommended screening scheme and/or advice | N |
| Cardigan Welsh Corgi* | Pastoral | 11 | 54, 88, 147-149, 197, 250, 403, 423, 432, 433 | 135 | >10 | 13 | ES, DM, rcd3-PRA, SCID | rcd3-PRA (DNA test) | ES | Y |
| Staffordshire Bull Terrier | Terrier | 11 | 1 | 5 | >10 | 71 | ES, HC-HSF4, L-2HGA, PHPV | ES, HC-HSF4 (DNA test), L-2HGA (DNA test) | Litter eye screening for PHPV | N |
| Italian Greyhound | Toy | 11 | 54, 233, 238, 243-245, 403, 423, 431, 432, 461 | 98 | >12 | 15 | No health tests relevant to this breed | No screening schemes relevant to this breed | No recommended screening scheme and/or advice | N |
| Gordon Setter | Gundog | 10 | 47, 64, 94, 95, 134, 163, 206, 214-216, 218, 431, 462 | 82 | >10 | 40 | ES, HDS, rcd4-PRA | HDS, rcd4-PRA (DNA test) | ES, NTPL1 | N |
| Afghan Hound | Hound | 10 | 4, 9, 11, 12, 13-16, 17-22, 23-25, 64, 135, 441 | 99 | >10 | 10 | No health tests relevant to this breed | No screening schemes relevant to this breed | No recommended screening scheme and/or advice | N |
| Whippet | Hound | 10 | 1 | 18 | >12 | 92 | MDR1, CEA/CH, PFK | No mandatory screening schemes | No recommended screening scheme and/or advice | N |
| Bearded Collie | Pastoral | 10 | 64, 90-94, 96, 217, 431, 432, 449 | 61 | >10 | 46 | HDS, CEA/CH | HDS | CEA/CH (DNA test) | N |
| Kerry Blue Terrier* | Terrier | 10 | 30, 151, 267, 268, 114, 423, 431, 432, 443 | 89 | >10 | 11 | DM, FVXI, vWD | No mandatory screening schemes | No recommended screening scheme and/or advice | Y |
| Soft-Coated Wheaton Terrier | Terrier | 10 | 54, 404-407, 431, 443 | 77 | >10 | 36 | ES, HDS, PLN, DM, kidney testing | No mandatory screening schemes | HDS, ES, kidney function test | N |
| Schnauzer | Utility | 10 | 30, 54, 134, 145, 199, 203, 391, 392, 423, 431, 432, 442, 443 | 83 | >10 | 17 | No health tests relevant to this breed | No screening schemes relevant to this breed | No recommended screening scheme and/or advice | N |
| Welsh Springer Spaniel | Gundog | 9 | 10, 163, 233, 250, 265, 335, 423, 424, 425, 431, 432 | 69 | >10 | 33 | ES, ES (Gonioscopy only), HDS | HDS, ES (Gonioscopy only) | ES | N |
| Manchester Terrier* | Terrier | 9 | 30, 114, 294, 423, 431, 432, 461 | 111 | >12 | 22 | vWD | No mandatory screening schemes | vWD (DNA test avaliable) | Y |
| Leonberger | Working | 9 | 250, 265, 283-288, 505 | 76 | >10 | 26 | ES, EDS, HDS, LPN1 | HDS, ES | NTPL1, EDS, LPN1 (additional test) | N |
| Portuguese Water Dog | Working | 9 | 30, 55, 64, 250, 351, 358-361, 423, 450, 462 | 132 | >10 | 6 | GM1, pcrd-PRA, IC | No mandatory screening schemes | prcd-PRA (additional test avaliable) | N |
| Flat Coated Retriever | Gundog | 8 | 1 | 35 | >10 | 63 | ES, ES (Gonioscopy only), HDS | HDS, ES (gonioscope only) | ES, NTPL1, NTPL2, NTPL3 | N |
| Basenji | Hound | 8 | 30, 76-86, 197, 423, 433 | 155 | >10 | 4 | ES, FS, PRA, PKD | No mandatory screening schemes | ES | N |
| Finnish Spitz | Hound | 8 | 92, 182, 431, 432, 491-494 | 166 | >10 | 5 | No health tests relevant to this breed | No screening schemes relevant to this breed | No recommended screening scheme and/or advice | N |
| King Charles Spaniel | Toy | 8 | 269-271, 423, 504, 505 | 95 | >12 | 7 | No health tests relevant to this breed | No screening schemes relevant to this breed | No recommended screening scheme and/or advice | N |
| Curly Coated Retriever* | Gundog | 7 | 343, 368, 369 , 423, 450, 462 | 127 | >10 | 12 | HDS, EIC, GSD | HDS | No recommended screening scheme and/or advice | Y |
| Borzoi | Hound | 7 | 30, 54, 107, 121, 124, 125, 176, 375, 423 | 119 | >10 | 7 | DM | No mandatory screening schemes | No recommended screening scheme and/or advice | N |
| Bedlington Terrier | Terrier | 7 | 96-99, 250, 423, 451, 452 | 65 | >10 | 12 | ES, COMMD1 | ES | COMMD1 (DNA test) | N |
| Australian Silky Terrier | Toy | 7 | 27, 64, 197, 327, 431-433, 462 | 175 | >12 | 7 | pcrd-PRA | No mandatory screening schemes | No recommended screening scheme and/or advice | N |
| Papillon | Toy | 7 | 54, 64, 114, 332, 333, 423, 450 | 55 | >12 | 24 | ES, cord1-PRA, PRA, vWD, slipping patella | No mandatory screening schemes | ES, slipping patella | N |
| Schipperke | Utility | 7 | 92, 388-390, 423, 450, 462 | 150 | >12 | 2 | MPSIIIB, pcrd-PRA | No mandatory screening schemes | MPSIIIB (DNA test) | N |
| Neapolitan Mastiff | Working | 7 | 64, 121, 297, 506, 507 | 93 | <10 | 6 | ES, HDS, heart testing | No mandatory screening schemes | HDS, ES, heart testing | N |
| Clumber Spaniel* | Gundog | 6 | 163, 265, 331, 418,423, 429, 432 | 94 | >10 | 16 | ES, HDS, PDP-1, PFK | HDS | PDP-1 (DNA test), ES | Y |
| Sussex Spaniel* | Gundog | 6 | 54, 163, 421-423 | 143 | >10 | 10 | HDS, PDP-1, PFK | No mandatory screening schemes | HDS | Y |
| Deerhound* | Hound | 6 | 171, 197, 255, 394, 395, 433, 523 | 85 | >10 | 17 | FVII, vWD, liver shunt screening (puppies) | No mandatory screening schemes | Litters screened for liver shunt prior to being sold | Y |
| Saluki | Hound | 6 | 27, 91, 376-379, 462 | 114 | >12 | 8 | No health tests relevant to this breed | No screening schemes relevant to this breed | No recommended screening scheme and/or advice | N |
| Briard | Pastoral | 6 | 139, 122, 224, 250, 423, 431, 474 | 107 | >10 | 12 | ES, HDS, CSNB | HDS, CSNB (DNA test) | ES | N |
| Hungarian Kuvasz | Pastoral | 6 | 54, 55, 64, 248, 462 | 207 | >10 | 0 | pcrd-PRA | No mandatory screening schemes | No recommended screening scheme and/or advice | N |
| Irish Terrier* | Terrier | 6 | 197, 253, 254, 423, 431, 444, 505 | 81 | >10 | 27 | No health tests relevant to this breed | No screening schemes relevant to this breed | No recommended screening scheme and/or advice | Y |
| Sealyham Terrier* | Terrier | 6 | 30, 54, 187, 250, 265, 403, 423, 430 | 146 | >10 | 3 | ES, PLL | PLL (DNA test) | ES | Y |
| Chinese Crested | Toy | 6 | 49, 54 150, 151, 250 | 64 | >12 | 21 | pcrd-PRA, DM, vWD, PLL | No mandatory screening schemes | PLL (DNA test), pcrd-PRA (DNA test) | N |
| Japanese Shiba Inu | Utility | 6 | 30, 250, 397-400, 535 | 92 | >10 | 10 | ES (Gonioscopy only), GM1 | ES (Gonioscopy only) | No recommended screening scheme and/or advice | N |
| German Wirehaired Pointer | Gundog | 5 | 30, 64, 205, 423 | 73 | >10 | 14 | HDS, EIC, HBIX, JEB, vWD | HDS, vWD (DNA test) | No recommended screening scheme and/or advice | N |
| Tervueren Belgian Shepherd | Pastoral | 5 | 96, 102-104, 454 | 115 | >10 | 13 | ES, HDS | ES, HDS | No recommended screening scheme and/or advice | N |
| Hungarian Puli | Pastoral | 5 | 54, 163, 249, 250, 431 | 145 | >10 | 3 | ES, HDS | HDS | ES | N |
| Lakeland Terrier* | Terrier | 5 | 96, 276, 403, 423, 462 | 86 | >10 | 19 | PLL | No mandatory screening schemes | No recommended screening scheme and/or advice | Y |
| Norfolk Terrier | Terrier | 5 | 188, 310, 311, 423 | 62 | >10 | 46 | No health tests relevant to this breed | No screening schemes relevant to this breed | No recommended screening scheme and/or advice | N |
| Skye Terrier* | Terrier | 5 | 176, 265, 393, 401, 402, 403 | 156 | >10 | 8 | No health tests relevant to this breed | No screening schemes relevant to this breed | No recommended screening scheme and/or advice | Y |
| Coton de Tulear | Toy | 5 | 54, 165-168 | 113 | >12 | 9 | BNA, CMR, HUU, vWD, DM, PH, CA | No mandatory screening schemes | No recommended screening scheme and/or advice | N |
| Havanese | Toy | 5 | 54, 171, 245, 265, 432 | 104 | >12 | 18 | HAVIII | No mandatory screening schemes | No recommended screening scheme and/or advice | N |
| German Pinscher | Working | 5 | 199, 201-204 | 181 | >10 | 3 | vWD | No mandatory screening schemes | No recommended screening scheme and/or advice | N |
| Irish Water Spaniel* | Gundog | 4 | 145, 419, 420, 423, 462 | 118 | >10 | 10 | HDS, PFK | HDS | No recommended screening scheme and/or advice | Y |
| Malinois Belgian Shepherd | Pastoral | 4 | 47, 100, 101, 453 | 125 | >10 | 3 | ES, HDS | ES, HDS | No recommended screening scheme and/or advice | N |
| Australian Terrier | Terrier | 4 | 64, 431, 432, 529 | 154 | >10 | 6 | vWD | No mandatory screening schemes | No recommended screening scheme and/or advice | N |
| Beauceron | Working | 4 | 33, 95, 450 | 177 | >10 | 4 | ES, HDS | HDS | NTPL1, NTPL2, ES (annual test), no puppy to leave breeder before 8 weeks of age | N |
| Dogue de Bordeaux | Working | 4 | 1 | 20 | >10 | 18 | HDS, CMR | HDS | No recommended screening scheme and/or advice | N |
| Irish Red & White Setter* | Gundog | 3 | 250, 251, 265 | 126 | >10 | 21 | ES, HDS, CLAD, rcd1-PRA, rcd4-PRA, vWD | ES, CLAD (DNA test), vWD (DNA test) | HDS | Y |
| Kooikerhondje | Gundog | 3 | 30, 272-274 | 178 | >10 | 0 | HNM, vWD | No mandatory screening schemes | No recommended screening scheme and/or advice | N |
| Large Munsterlander | Gundog | 3 | 64, 211, 281, 282 | 116 | >10 | 10 | ES, HDS, BHFD, HUU | HDS, ES | HUU (DNA test) | N |
| Nova Scotia Duck Trolling Retriever | Gundog | 3 | 54, 63, 318, 430 | 96 | >10 | 19 | ES, HDS, CEA/CH, pcrd-PRA, DM | HDS, pcrd-PRA (DNA test), CEA/CH (DNA test) | ES, NTPL1, NTPL2, dogs <2yrs not to be used as stud, DM (additional DNA test) | N |
| American Water Spaniel | Gundog | 3 | 423, 450, 480 | 206 | >10 | 0 | No health tests relevant to this breed | No screening schemes relevant to this breed | No recommended screening scheme and/or advice | N |
| Field Spaniel* | Gundog | 3 | 423 | 144 | >10 | 5 | ES, HDS, PFK, heart testing | HDS | Heart testing, ES, NTPL1, NTPL2, dogs <12 months not to be used as stud | Y |
| Foxhound | Hound | 3 | 36, 38, 54, 183, 495 | 190 | >10 | 0 | No health tests relevant to this breed | No screening schemes relevant to this breed | No recommended screening scheme and/or advice | N |
| Ibizan Hound | Hound | 3 | 27, 54, 96 | 182 | >10 | 3 | No health tests relevant to this breed | No screening schemes relevant to this breed | No recommended screening scheme and/or advice | N |
| Otterhound* | Hound | 3 | 121, 163, 330, 331 | 158 | >10 | 2 | EDS, HDS, GT | HDS, EDS | Breed club to advice on epilepsy | Y |
| Lancashire Heeler* | Pastoral | 3 | 250, 277, 278 | 112 | >12 | 6 | ES, CEA/CH, PLL | ES, PLL (DNA test) | CEA/CH (DNA test) | Y |
| Swedish Lapphund | Pastoral | 3 | 181, 250, 428 | 201 | >10 | 1 | pcrd-PRA, GSDII | No mandatory screening schemes | No recommended screening scheme and/or advice | N |
| Dandie Dinmont Terrier* | Terrier | 3 | 169-171, 403 | 123 | >10 | 13 | ES | No mandatory screening schemes | ES | Y |
| Parson Russell Terrier | Terrier | 3 | 211, 250, 277, 334 | 58 | >12 | 37 | ES, HUU, LOA, PLL, SCA | No mandatory screening schemes | ES, PLL (DNA test), LOA (additional DNA test), SCA (additional DNA test) | N |
| Welsh Terrier* | Terrier | 3 | 49, 187, 265, 423, 436 | 75 | >10 | 25 | PLL | No mandatory screening schemes | No recommended screening scheme and/or advice | Y |
| Affenpinscher | Toy | 3 | 2, 3, 4, 5 | 117 | >12 | 15 | Patellar testing | No mandatory screening schemes | Patella testing | N |
| Japanese Chin | Toy | 3 | 64, 423, 432 | 88 | >10 | 14 | No health tests relevant to this breed | No screening schemes relevant to this breed | No recommended screening scheme and/or advice | N |
| Tibetan Spaniel | Utility | 3 | 54, 250, 265, 434, 435 | 91 | >12 | 9 | ES, PRA | ES | NTPL2, NTPL5, rcd3-PRA (additional health test) | N |
| Greater Swiss Mountain Dog | Working | 3 | 47, 227, 228 | 179 | >10 | 3 | No health tests relevant to this breed | No screening schemes relevant to this breed | No recommended screening scheme and/or advice | N |
| Italian Spinone | Gundog | 2 | 258, 259 | 68 | >10 | 23 | HDS, CA | HDS | NTPL1, CA (DNA linkage) test | N |
| Lagotto Romagnola | Gundog | 2 | 279, 280 | 164 | >10 | 4 | JE, IC | No screening schemes relevant to this breed | No recommended screening scheme and/or advice | N |
| Spanish Water Dog | Gundog | 2 | 250, 427 | 110 | >10 | 14 | ES, ES (Gonioscopy only), HDS, CH, pcrd-PRA, HUU | HDS, ES (Gonioscopy only), prcd-PRA (DNA test) | NTPL1, NTPL2, ES (annual test) | N |
| Anatolian Shepherd | Pastoral | 2 | 39, 54 | 157 | >10 | 1 | HDS | HDS | No recommended screening scheme and/or advice | N |
| Smooth Collie* | Pastoral | 2 | 63, 250 | 141 | >10 | 8 | ES, HDS, CCN, CEA/CH, DM, MDR1, rcd2-PRA, NCL | ES (annual test) | CEA/CH (DNA test), HDS, ES (litter screening), MDR1 (DNA test), DM (additional DNA test), rcd2-PRA (additional DNA test) | Y |
| Estrela Mountain Dog | Pastoral | 2 | 178, 179 | 165 | >10 | 1 | HDS | HDS | No recommended screening scheme and/or advice | N |
| Finnish Lapphund | Pastoral | 2 | 180, 181 | 151 | >10 | 15 | ES, HDS, pcrd-PRA, GSDII | HDS, ES | pcrd-PRA (DNA test), GSDII (additional DNA test) | N |
| Polish Lowland Sheepdog | Pastoral | 2 | 527, 528 | 147 | >10 | 10 | ES, HDS, rcd4-PRA | HDS | ES | N |
| Miniture Bull Terrier* | Terrier | 2 | 277, 423, 530 | 90 | >10 | 21 | ES, PLL, heart and kidney testing | PLL (DNA test) | Heart and kidney testing, ES | Y |
| Glen Of Imaal Terrier* | Terrier | 2 | 88, 213 | 149 | >10 | 6 | ES, crd3-PRA | crd3-PRA (DNA test) | ES | Y |
| Norwich Terrier* | Terrier | 2 | 96, 188, 423 | 102 | >10 | 15 | PLL | No mandatory screening schemes | No recommended screening scheme and/or advice | Y |
| Griffon Bruxellois | Toy | 2 | 146, 423, 432 | 97 | >12 | 11 | No health tests relevant to this breed | No screening schemes relevant to this breed | No recommended screening scheme and/or advice | N |
| Japanese Akita Inu | Utility | 2 | 206, 462 | 160 | >10 | 4 | No health tests relevant to this breed | No screening schemes relevant to this breed | No recommended screening scheme and/or advice | N |
| Canadian Eskimo Dog | Working | 2 | 265, 432 | 186 | >10 | 1 | No health tests relevant to this breed | No screening schemes relevant to this breed | No recommended screening scheme and/or advice | N |
| Entlebucher Mountain Dog | Working | 2 | 531, 532 | 191 | >12 | 2 | pcrd-PRA | No mandatory screening schemes | No recommended screening scheme and/or advice | N |
| Hovawart | Working | 2 | 246, 247 | 170 | >10 | 7 | HDS, DM, thyroid function test | HDS | Thyroid function test | N |
| Russian Black Terrier | Working | 2 | 120, 370 | 134 | >10 | 7 | HUU | No mandatory screening schemes | NTPL1, HUU (DNA test) | N |
| Tibetan Mastiff | Working | 2 | 163, 296 | 148 | >10 | 4 | HDS | HDS | No recommended screening scheme and/or advice | N |
| German Longhaired Pointer | Gundog | 1 | 121 | 173 | >10 | 4 | JEB, vWD | No mandatory screening schemes | No recommended screening scheme and/or advice | N |
| Grand Basset Griffon Vendeen | Hound | 1 | 87 | 128 | >10 | 7 | No health tests relevant to this breed | No screening schemes relevant to this breed | No recommended screening scheme and/or advice | N |
| Petit Basset Griffon Vendeen | Hound | 1 | 88 | 101 | >10 | 18 | No health tests relevant to this breed | No screening schemes relevant to this breed | No recommended screening scheme and/or advice | N |
| Bavarian Mountain Hound | Hound | 1 | 89 | 163 | >10 | 2 | HDS | No mandatory screening schemes | Aptitude test, NTPL1, HDS | N |
| Grand Bleu de Gacogne | Hound | 1 | 121 | 192 | >10 | 0 | No health tests relevant to this breed | No screening schemes relevant to this breed | No recommended screening scheme and/or advice | N |
| Pharaoh Hound | Hound | 1 | 344 | 169 | >10 | 8 | Slipping patella | No mandatory screening schemes | Slipping patella | N |
| Portuguese Podengo | Hound | 1 | 521 | 142 | >10 | 9 | No health tests relevant to this breed | No screening schemes relevant to this breed | No recommended screening scheme and/or advice | N |
| Sloughi | Hound | 1 | 250, 265 | 187 | >10 | 0 | rcd1-PRA | No mandatory screening schemes | No recommended screening scheme and/or advice | N |
| Catalan Sheepdog | Pastoral | 1 | 426 | 172 | >10 | 6 | ES, HDS | HDS | ES | N |
| Norwegian Buhund | Pastoral | 1 | 250, 312 | 167 | >10 | 3 | ES, HDS | ES, HDS | No recommended screening scheme and/or advice | N |
| Pyrenean Sheepdog (Long haired) | Pastoral | 1 | 363 | 174 | >10 | 6 | HDS, patent ductus arteriosus testing (puppies) | No mandatory screening schemes | HDS, heart testing (puppies) for patent ductus arteriosus (PDA) | N |
| Lowchen (Little Lion Dog) | Toy | 1 | 54 | 122 | >12 | 9 | No health tests relevant to this breed | No screening schemes relevant to this breed | No recommended screening scheme and/or advice | N |
| Canaan Dog | Utility | 1 | 145 | 180 | >10 | 2 | ES, HDS, spillping patella | No mandatory screening schemes | ES, HDS, slipping patellas, check for cryptorchidism | N |
| Japanese Spitz | Utility | 1 | 260 | 106 | >12 | 11 | MD | No mandatory screening schemes | No recommended screening scheme and/or advice | N |
| Korean Jindo | Utility | 1 | 275 | 184 | >10 | 1 | No health tests relevant to this breed | No screening schemes relevant to this breed | No recommended screening scheme and/or advice | N |
| Mexican Hairless | Utility | 1 | 510 | 204 | >10 | 0 | No health tests relevant to this breed | No screening schemes relevant to this breed | No recommended screening scheme and/or advice | N |
| Bracco Italiano | Gundog | N/A | N/A | 153 | >10 | 5 | ES, EDS, HDS | ES, EDS, HDS | No recommended screening scheme and/or advice | N |
| Hungarian Wire Haired Vizsla | Gundog | N/A | N/A | 79 | >10 | 31 | HDS, HUU | HDS | NTPL1, NTPL2 | N |
| Korthals Griffon | Gundog | N/A | N/A | 168 | >10 | 3 | EDS, HDS | EDS, HDS | No recommended screening scheme and/or advice | N |
| Portuguese Pointer | Gundog | N/A | N/A | 198 | >10 | 1 | No health tests relevant to this breed | No screening schemes relevant to this breed | No recommended screening scheme and/or advice | N |
| Slovakian Rough Haired Pointer | Gundog | N/A | N/A | 152 | >10 | 3 | No health tests relevant to this breed | No screening schemes relevant to this breed | HDS | N |
| Small Munsterlander | Gundog | N/A | N/A | 199 | >10 | 0 | rcd4-PRA | No mandatory screening schemes | No recommended screening scheme and/or advice | N |
| Azawakh | Hound | N/A | N/A | 197 | <10 | 0 | No health tests relevant to this breed | No screening schemes relevant to this breed | No recommended screening scheme and/or advice | N |
| Basset Bleu de Gascogne | Hound | N/A | N/A | 194 | >10 | 0 | No health tests relevant to this breed | No screening schemes relevant to this breed | No recommended screening scheme and/or advice | N |
| Basset Fave de Bretagne | Hound | N/A | N/A | 130 | >10 | 14 | No health tests relevant to this breed | No screening schemes relevant to this breed | No recommended screening scheme and/or advice | N |
| Cirneco Dell'Etna | Hound | N/A | N/A | 183 | >12 | 3 | No health tests relevant to this breed | No screening schemes relevant to this breed | NTPL1 | N |
| Hamiltonstovare | Hound | N/A | N/A | 176 | >10 | 3 | No health tests relevant to this breed | No screening schemes relevant to this breed | NTPL1, NTPL2 | N |
| Segugio Italiano | Hound | N/A | N/A | 203 | >10 | 0 | No health tests relevant to this breed | No screening schemes relevant to this breed | No recommended screening scheme and/or advice | N |
| Groenedael Belgian Shepherd | Pastoral | N/A | N/A | 139 | >10 | 11 | ES, HDS | ES, HDS | No recommended screening scheme and/or advice | N |
| Laekenois Belgian Shepherd | Pastoral | N/A | N/A | 195 | >10 | 0 | ES, HDS | ES, HDS | No recommended screening scheme and/or advice | N |
| Bergamasco | Pastoral | N/A | N/A | 193 | >10 | 4 | No health tests relevant to this breed | No screening schemes relevant to this breed | No recommended screening scheme and/or advice | N |
| Komondor | Pastoral | N/A | N/A | 188 | <10 | 0 | No health tests relevant to this breed | No screening schemes relevant to this breed | No recommended screening scheme and/or advice | N |
| Maremma Sheepdog | Pastoral | N/A | N/A | 171 | >10 | 2 | HDS | HDS | No recommended screening scheme and/or advice | N |
| Swedish Vallhund | Pastoral | N/A | N/A | 159 | >10 | 7 | HDS | HDS | No recommended screening scheme and/or advice | N |
| Turkish Kangal Dog | Pastoral | N/A | N/A | 202 | >10 | 0 | No health tests relevant to this breed | No screening schemes relevant to this breed | No recommended screening scheme and/or advice | N |
| Cesky Terrier | Terrier | N/A | N/A | 162 | >10 | 6 | ES | No mandatory screening schemes | ES | N |
| Bolognese | Toy | N/A | N/A | 124 | >12 | 21 | No health tests relevant to this breed | No screening schemes relevant to this breed | No recommended screening scheme and/or advice | N |
| English Toy Terrier (Black & Tan)* | Toy | N/A | N/A | 121 | >12 | 14 | BAER, patella luxation | No mandatory screening schemes | BAER testing, patella luxation screening | Y |
| Eurasier | Utility | N/A | N/A | 133 | >10 | 4 | JEB | No mandatory screening schemes | No recommended screening scheme and/or advice | N |
| German Spitz Klein | Utility | N/A | N/A | 109 | >12 | 12 | ES, HUU | No mandatory screening schemes | ES | N |
| German Spitz Mittel | Utility | N/A | N/A | 136 | >12 | 8 | ES, HUU | No mandatory screening schemes | ES | N |
| Mexican Hairless (Intermediate) | Utility | N/A | N/A | 189 | >10 | 0 | No health tests relevant to this breed | No screening schemes relevant to this breed | No recommended screening scheme and/or advice | N |
| Mexican Hairless (Miniture) | Utility | N/A | N/A | 196 | >10 | 0 | No health tests relevant to this breed | No screening schemes relevant to this breed | No recommended screening scheme and/or advice | N |
| Mexican Hairless (Standard) | Utility | N/A | N/A | 200 | >10 | 0 | No health tests relevant to this breed | No screening schemes relevant to this breed | No recommended screening scheme and/or advice | N |
| Greenland Dog | Working | N/A | N/A | 185 | >10 | 2 | No health tests relevant to this breed | No screening schemes relevant to this breed | No recommended screening scheme and/or advice | N |
| Pyrenean Mastiff | Working | N/A | N/A | 205 | <10 | 0 | ES, HDS | No mandatory screening schemes | ES, HDS | N |

Summary of inherited disorders in pedigree dogs. Table organized by number of inherited disorders in the breed. *****, vulnerable UK breed status (UK Kennel Club); Number of total disorders, all known (and suspected) inherited and increased risk disorders for the breed (full details in electronic supplementary material); N/A, no information currently avaliable for this breed; Reference Source, numbered with brief citation at the end the table (full citations in electronic supplementary material); Popularity Rank, number of UK Kennel Club registration statistics for the past 10 years averaged (2004-2013 inclusive, full details in electronic supplementary methods); ^+^, UK Kennel Club Breed Specific Health Tests (as of March 2014); AF, anal furunculosis; AMP, Alaskan Malamute polyneuropathy; ARVC, arrhythmogenic right ventricular cardiomyopathy; BAER, deafness; BHFD, black hair follicular dysplasia; BNA, Bandera’s neonatal ataxia; BTPKD, Bull Terrier polycystic kidney disease; CA, cerebellar ataxia; CC/DE, curlycoat/dryeye; CCN, canine cyclic neutropenia; CD, cone degeneration; CEA/CH, collie eye anomally/ choroidal hypoplasia; CH, congenital hypothyreosis/hypothyroidism; CL, ceroid lipofuscinosis; CLAD, canine leucocyte adhesion deficiency; CM, cobalamin malabsorption; CM/SM, Chiari malformation/syringomyelia scheme; CMHM, BVA/KC CMSM Scheme; CMR, canine multifocal retinopathy; CNM, centronuclear myopathy; COMMD1, Copper toxicosis; cord1-PRA, progressive retinal atrophy-cone and rod deterioration-1; cord2-PRA, progressive retinal atrophy-cone and rod deterioration-2; crd3-PRA, progressive retinal atrophy-cone and rod dystropy-3; CSNB, congenital stationary night blindness; CU, cystinuria; DCM, dilated cardiomyopathy; DFM, dwarfism; DM, degenerative myelopathy; ED, ectodermal dysplasia; EDS, BVA/KC Elbow Dysplasia Scheme; EF, episodic falling; EIC, exercise induced collapse; ES, BVA/KC/ISDS Eye Scheme (including annual eye testing); FN, familial nephropathy; FS, Fanconi syndrome; Fuco, fucosidosis; FVII, factor VII deficiency; FVXI, factor XI deficiency; GCL, globoid cell leukodystrophy; GM1, gangliosidosis; GN, Greyhound neuropathy; GSD, glycogenesis type IIIa; GSDI, glycogen storage disease type I (Pompe's disease); GSDII, glycogen storage disease type II (Pompe's disease) GT, Glanzmann's thronbasthenia type1; HAVIII, Haemophilia A (factor VIII deficiency); HBIXl, Haemophilia B (factor IX deficiency); HC-HSF4, hereditary cataracts; HDS, BVA/KC Hip Dysplasia Scheme; HN, hereditary nephritis; HNM, hereditary necrotising myelopathy; HNP, hereditary nasal parakeratosis; HUU, hyperuricosuria; IC, improper coat; ICT-A, ichthyosis; IRIDA, iron refractory iron deficiency anemia; JE, juvenile epilepsy; JEB, junctional epidermolysis bullosa; L-2HGA, L-2-hydroxyglutaric aciduria; LD, Lafora's disease; LOA, late onset ataxia; LPN1, Leonberger polyneuropathy; MD, muscular dystrophy; MDR1, multiple drug sensitivity/resistance; MLS, Musladin-Leuke syndrome; MPS, mucopolysaccharidosis type VI; MPSIIIA, mucopolysaccharidosis type IIIA; MPSIIIB, mucopolysaccharidosis type IIIB; MPSVII, mucopolysaccharidosis type VII; MYO, myotonia congenital; N/A, No information currently avaliable for this breed; NCCD, neonatal cerebellar cortical degeneration; NCL, neuronal ceroid lipofuscinosis; NE, neonatal encephalopathy; NHPH4, progressive retinal atrophy CRD; NPY, narcolepsy; NTPL1, bitches < 2 yrs not to produce a litter; NTPL2, bitches not to produce > 1 litter in a 12-month period; NTPL3, bitches not to produce > 3 litters in a lifetime; NTPL4, bitches not to produce > 4 litters in a lifetime; NTPL5, bitches not to produce > 5 litters in a lifetime; OI, osteogenesis imperfecta; OSD, retinal/ocularskeletal dysplasia; PCD, primary ciliary dyskinesia; pcrd-PRA, progressive rod cone degeneration -progressive retinal atrophy; PDE, Pug dog encephalitis; PDP-1, pyruvate dehydrogenase phosphate 1 deficiency; PFK, phosphofructokinase deficiency; PH, primary hyperoxaluria; PHPT, primary hyperparathyroidism; PHPV, persistent hyperplastic primary vitreous; PKD, pyruvate kinase deficiency; PLL, primary lens luxation; PLN, protein losing nephropathy; POAG, primary open angle glaucoma; PRA, progressive retinal atrophy; PRA-D, progressive retinal atrophy-dominant; rcd1-PRA, progressive retinal atrophy-rod-cone degeneration-1; rcd2-PRA, progressive retinal atrophy-rod-cone degeneration-2; rcd3-PRA, progressive retinal atrophy-rod-cone degeneration-3; rcd4-PRA, progressive retinal atrophy-rod-cone degeneration-4; RCND, renal cystadenocarcinoma nodular dermatofibrosis; SAN, sensory ataxic neuropathy; SAT, sebaceous adenitis testing; SCA, spinocerebellar ataxia; SCID, X-linked severe combined immunodeficiency; SD, Startle disease; TNS, trapped neutrophil syndrome; TPA, thrombopathia; VDD, vitamin D deficiency (Rickets) type 2; vWD, von Willebrand disease (types 1, 2, 3); XL-PRA, X-linked progressive retinal atrophy.

NOTE: This is not an exhaustive list of all inherited disorders in pedigree dogs and new DNA disease tests are continously being developed.

**1.** Asher et al. (2009) **2.** Waldman (1995) **3.** Piek et al. (1996) **4.** O’Brien (1975) **5.** Hendricks (1992) **6.** Fasanella et al. (2010) **7.** Torrez & Hunt (2006) **8.** Bannasch et al. (2010) **9.** Roberts & Helper (1972) **10.** Barnett (1980) **11.** Sewell et al. (1997) **12.** Anderson et al. (1989) **13.** Cummings & deLahunta (1978) **14.** Averill & Bronson (1977) **15.** Jones & Richards (1977) **16.** Targett & McInnes (1998) **17.** Silverman & Kuttel (1982) **18.** Fossum et al. (1986) **19.** Williams & Duncan (1986) **20**. Gelzer et al. (1997) **21.** Neath et al. (2000) **22.** Johnston et al. (1984) **23.** Grondalen (1973) **24.** Curtis & Barnett (1981) **25.** Curtis & Barnett (1983) **26.** Miller & Dunstan (1993) **27.** Summers et al. (1995) **28.** Cordy & Snelbaker (1952) **29.** Dice (1976) **30.** Brooks (1999) **31**. Gu et al. (1999) **32.** Dodds et al. (1981) **33.** Villamil et al. (2011) **34.** Reetz et al. (1977) **35.** Strain (1996) **36.** Adams (1956) **37.** Hiraide & Paparella (1988) **38.** Strain et al. (2009) **39.** Temizsoylu & Avki (2003) **40.** Sisk et al. (1990) **41.** Vandevelde & Fatzer (1980) **42.** Wood et al. (1987) **43.** Studdert et al. (1991) **44.** Tisdall et al. (1994) **45.** Krotscheck et al. (2007) **46.** Van Steenbeek et al. (2011) **47.** Clements et al. (2007) **48.** Collier et al. (1989) **49.** Gould et al. (2011) **50.** Brenner et al. (1997) **51.** De Bosschere et al. (2002) **52.** Li et al. (2006) **53.** Laratta et al. (1988) **54.** Strain (2004) **55.** Zangerl et al. (2006) **56.** Finnigan et al. (2007) **57.** Gracis et al. (2000) **58.** Wood & Patterson (2001) **59.** Sommerland et al. (2010) **60.** Rubin et al. (1991) **61.** Lowe et al. (2003) **62.** Munyard et al. (2007) **63.** Parker et al. (2007) **64.** LaFond et al. (2002) **65.** Gelatt et al. (1981) **66.** Sponenberg & Bowling (1985) **67.** Senders et al. (1986) **68.** Weissl et al. (2011) **69.** Barbet et al. (2009) **70.** Gramer et al. (2010) **71.** Mellersh et al. (2009) **72.** O’Brien & Katz (2008) **73.** Katz et al. (2011) **74.** Guziewicz et al. (2007) **75.** Hoffmann et al. (2012) **76.** Breitschwerdt et al. (1982) **77.** Breitschwerdt et al. (1991) **78.**  Olivero et al. (2011) **79.** Barnett & Knight (1969) **80.** Mason (1976) **81.** James (1991) **82.** Searcy et al. (1971) **83.** Giger & Noble (1991) **84.** Whitney & Lothrop (1995) **85.** Bovee et al. (1978) **86.** Yearley et al. (2004) **87.** Klarenbeek et al. (2007) **88.** Parker et al. (2009) **89.** Flegel et al. (2007) **90.** Harper (1978) **91.** Schmutz et al. (1998) **92.** Ihrke et al. (1985**) 93.** Oberbauer et al. (2002) **94**. Ovrebo et al. (2001) **95.** Scott et al. (1995) **96.** Krohne (2001) **97.** Johnson et al. (1980) **98.** Yuzbasiyan et al. (1997) **99.** Dietz (1985) **100.** Ramos-Vara et al. (2004) **101.** Kleiter et al. (2011) **102.** Seppälä et al. (2012) **103.** Mahaffey et al. (1978) **104.** Lubbes et al. (2009) **105.** Beuing et al. (2005) **106.** Kathmann et al. (1999) **107.** Dodds (1989) **108.** Minkus et al. (1994) **109.** Carmichael ey al., (1996) **110.** Palmer et al. (1987) **111.** Abadie et al. (2009) **112.** Weissenbock et al. (1996) **113.** Willis (2000) **114.** Brewer et al. (1998) **115.** Chaudieu et al. (2004) **116.** Hayashi et al. (2004) **117.** Hagman et al. (2011) **118.** Wininger et al. (2011) **119.** Barnett (1985) **120.** Bende & Nemeth (2004) **121.** Evans & Adams (2010a) **122.** Janutta & Distl (2006) **123.** Hamil (1990) **124.** Conaway et al. (1985) **125.** Storey et al. (2005) **126.** Mellersh et al. (2007) **127.** Edmonds et al. (1972) **128.** Cooley & Dice (1990) **129.** Hayes et al. (1975) **130.** Coyne & Fingland (1992) **131.** Hayes & Wilson (1986) **132.** Eneroth et al. (1999) **133.** Peeters & Ubbink (1994) **134.** Comhaire & Snaps (2008) **135.** Burbidge (1995) **136.** Van Rensberg et al. (1992) **137.** Van der Linde-Sipman (1987) **138.** Temwichitir et al. (2010) **139.** Lightfoot et al. (1996) **140.** Richtsmeier et al. (1994) **141.** Cork et al. (1991)  **142.** Sack et al. (1984) **143.** Hubert et al. (1987) **144.** Higgins et al. (1998) **145.** Johnson & Patterson (2003) **146.** Knowler et al. (2014) **147.** Petersen-Jones et al. (1999)  **148.** Somberg et al. (1995) **149.** Tanaka et al. (2001) **150.** O’Brien et al. (2004) **151.** O’Brien et al. (2005) **152.** Collins et al. (1992) **153.** Kirberger & Stander (2007) **154.** Corcoran et al. (1994) **155.** Edwards et al. (1992) **156.** Farrow & Malik (1981) **157.** Ramos-Vara et al. (2000) **158.** Buchanan (1992) **159.** Gonsalves-Hubers (2005) **160.** Booth (1998) **161.** Vandevelde et al. (1978) **162.** Cerundolo & Lloyd (1998) **163.** Orthopedic Foundation (2003) **164.** Batchelor et al. (2007) **165.** Tipold et al. (2000) **166.** Coates et al. (2002) **167.** Vidgren et al. (2012) **168.** Grahn et al. (2006) **169.** Scholten-Sloof et al. (1992) **170.** Ahonen et al. (2013a)  **171.** Tobias & Rohrbach (2003) **172.** Kijas et al. (2003) **173.** Katz et al. (2005) **174.** Boari et al. (1994) **175.** Slater et al. (1991) **176.** Nachreiner et al. (2002) **177.** Marfe et al. (2012) **178.** Ginja et al. (2009) **179.** Lobo et al. (2010) **180.** Aguirre-Hernandez et al. (2007) **181.** Seppälä et al. (2013) **182.** Jeserevics et al. (2007) **183.** Mason et al. (1996) **184.** von Sandersleben et al. (1986) **185.** Jenkins et al. (1976) **186.** Bjorck et al. (1962) **187.** Gelatt & Mackay (2004) **188.** Curtis & Barnett (1980) **189.** Patterson (1989) **190.** Ordeix et al. (2009) **191.** Rohdin et al. (2010) **192.** Slappendel (1975) **193.** Done et al. (1975) **194.** Tanaka et al. (2003) **195.** Hansen (1952) **196.** Poncet et al. (2005) **197.** Hoppe & Denneberg (2001) **198.** Gaudet (1985) **199.** Oliveira et al. (2011) **200.** Mazzuccheli et al. (2012) **201.** Pfahler et al. (2014) **202.** Leppanen et al. (2001) **203.** Kim et al. (2005) **204.** Philipp et al. (2011) **205.** van Dongen et al. (2001) **206.** Genevois et al. (2008)  **207.** Greco et al. (1991)  **208.** Fyfe et al. (1991) **209.** Kidd et al. (2004) **210.** Fyfe et al. (2010) **211.** Karmi et al. (2010) **212.** Wilbe et al. (2010) **213.** Kropatsch et al. (2010) **214.** Tiemeyer et al. (1984) **215.** Glickman et al. (2000) **216.** Yaeger et al. (2000) **217.** Harlos (2010) **218.** Downs et al. (2013)  **219.** Bingel & Sande (1994) **220.** Coppens et al. (2000) **221.** Boudreaux et al. (1996) **222.** Golden et al. (1980) **223.** Grahn et al. (1998) **224.** Breur et al. (2001) **225**. Fogh & Fogh (1988) **226.** Wright & Brownlie (1985) **227.** Ekenstedt et al. (2011) **228.** Boudreaux & Martin (2011) **229.** Lynch (2007) **230.** Skelly et al. (1997) **231.** Slatter et al. (1980) **232.** Bagshaw et al. (1978) **233.** Bennett (1974) **234.** Cowan et al. (1997) **235.** Shiel et al. (2010) **236.** Gunby et al. (2004) **237.** Karlsson et al. (2013) **238.** Sams & Muir (1988) **239.** Gaughan & Bruyette (2001) **240.** Brenner et al. (2009) **241.** Drögemüller et al. (2010) **242.** Court et al. (1999) **243.** Pedersen et al. (2011) **244.** Roux et al. (2011) **245.** Starr et al. (2007) **246.** Rugbjerg et al. (2003) **247.** Ferm et al. (2009) **248.** Hazlett et al. (2005) **249.** Ruth (2012) **250.** Crispin & Warren (2008) **251.** Gu et al. (2004)  **252.** Presthus & Nordstoga (1993) **253.** Wentink et al. (1972) **254.** Binder et al. (2000) **255.** Martin et al. (2008)  **256.** Clercx et al. (2003) **257.** Casal et al. (2006) **258.** Wheeler & Rusbridge (1996) **259.** Hill (2006) **260.** Jones et al. (2004) **261.** Werner et al. (2005) **262.** Hall & Wallace (1996)  **263.** Goldstein et al. (2007) **264.** Buchanan & Patterson (2003) **265.** Gelatt (2007) **266.** Mausberg et al. (2008) **267.** Knowler et al. (1994) **268.** Kniazev et al. (2003) **269.** Wright et al. (1987) **270.** Swenson et al. (1996) **271.** Buchanan et al. (1997) **272.** Mandigers et al. (1993) **273.** Schulze et al. (1998) **274.** van Oost et al. (2004) **275.** Yamato et al. (1999) **276.** Lowe & King (2004) **277.** Sargan et al. (2007) **278.** Bedford (1998)  **279.** Jokinen et al. (2007a) **280.** Jokinen et al. (2007b) **281.** Coppieters et al. (2012) **282.** von Bomhard et al. (2006) **283.** Smallwood & Barsanti (1995) **284.** Hultin et al. (2011) **285.** Chetboul et al. (2003) **286.** Heinrich et al. (2006) **287.** Anfinsen et al. (2011) **288.** Krontveit et al. (2012) **289.** Hayes et al. (1985) **290.** Brix et al. (1995) **291.** O’Brien et al. (1999) **292.** Stalis et al. (1995) **293.** Parker et al. (2012) **294.** Vasseur et al. (1989) **295.** Osborne et al. (1999) **296.** Cummings et al. (1981) **297.** Morgan et al. (1993) **298.** Snaps et al. (1998) **299.** Kazmierski et al. (2001) **300.** Reist-Marti et al. (2012) **301.** Watson et al. (1999) **302.** Henthorn et al. (2000) **303.** Groondalen (1981) **304.** Kirberger & Fourie (1998) **305.** Kittleson & Kienle (1998) **306.** Fascetti et al. (2003) **307.** Lipsitz et al. (1999) **308.** Wilke et al. (2006) **309.** Young et al. (2006) **310.** Barnhart et al. (2004) **311.** Gelain et al. (2010) **312.** Bjerkas & Haaland (1995) **313.** Stannard & Pulley (1975) **314.** Wiersma et al. (2005) **315.** Oshima et al. (2004) **316.** Melniczek et al. (1999) **317.** Kyöstilä et al. (2013) **318.** Hughes et al. (2007) **319.** Green & Lantz (1978) **320.** Koch (1972) **321.** Merveille et al. (2014) **322.** Anniko et al. (1977) **323.** Williams & Maggio-Price (1984) **324.** Breitschwerdt et al. (1992) **325.** Watson & Dixon (1977) **326.** Steinberg et al. (2000) **327.** Hunt (2004) **328.** Wieczorek et al. (2006) **329.** Geyer et al. (2005) **330.** Boudreaux & Catalfamo (2001) **331.**  Wang et al. (1999) **332.** Nibe et al. (2007) **333.** Ahonen et al. (2013b) **334.** Forman et al. (2013) **335.** Priester (1974) **336.** Poncet et al. (2006) **337.** Petrick (1996) **338.** Goggin et al. (1970) **339.** Oswald & Orton (1993) **340.** Moore & Thorton (1983) **341.** Coates et al. (2007) **342.** Smith et al. (2011)  **343.** Minor et al. (2011) **344.** Campbell & Crow (2010) **345.** Neer et al. (1995) **346.** Nowacka et al. (2005) **347.** Cummings et al. (1981) **348.** Whitbread et al. (1983) **349.** Henthorn et al. (2004) **350.** Klein et al. (1988) **351.** Brooks et al. (2008) **352.** Tidholm (1997) **353.** Buback et al. (1996)  **354.** Varshney (2007) **355.** Takada et al. (2002) **356.** Mausberg et al. (2007) **357.** Bergstrom et al. (2006) **358.** Alroy et al. (2005) **359.** Wang et al. (2000) **360.** Oberbauer et al. (2006) **361.** Parker et al. (2010) **362.** Gabriel et al. (2006) **363.** Kemp et al. (2009) **364.** Gelatt et al. (1979) **365.** Johnson et al. (1988) **366.** Cerundolo et al. (2005) **367.** Awano et al. (2009) **368.** Coward (1989) **369.** Gregory et al. (2007) **370.** Lohmann & Klesen (1997) **371.** Martin & Leipold (1974) **372.** Bech-Nielsen et al. (1978) **373.** Szczulowska (1967) **374.** Villagomez & Alonso (1998) **375.** Kammermann et al. (1971) **376.** Appleby et al. (1982) **377.** Kent et al. (1999) **378.** Ogburn et al. (1981) **379.** Hargis et al. (1991) **380.** Kramer et al. (1988) **381.** Kimmel et al. (2002) **382.** Zheng et al. (1994) **383.** Acland & Aguirre (1991) **384.** McCaw & Aronson (1984) **385.** Cummings et al. (1986) **386.** Ling et al. (1979) **387.** Mulvihull & Priester (1973) **388.** Knowles et al. (1993) **389.** Delauche et al. (1998) **390.** Ellinwood et al. (2003)  **391.** Paltrinieri et al. (2007) **392.** Gillard et al. (2014) **393.** Holt & Moore (1995) **394.** Breur et al. (1989) **395.** Philips et al. (2007) **396.** Ashton et al. (1968) **397.** Fujise et al. (1997) **398.** Yamato et al. (2000) **399.** Kato et al. (2006) **400.** Takeuchi et al. (2009) **401.** McGrotty et al. (2003) **402.** Lau (1977) **403.** Gelatt et al. (2008)  **404.** Peterson et al. (1996) **405.** Van der Woerdt et al. (1995) **406.** Littman et al. (2000) **407.** Eriksen & Grondalen (1984) **408.** Kittleson et al. (1997) **409.** Yakely (1971) **410.** Giger et al. (1992) **411.** Parry et al. (1988) **412.** Miller et al. (2000) **413.** Meyers-Wallen et al. (1995) **414.** Callan et al. (1995) **415.** Kwochka & Rademakers (1989)  **416.** Scott-Moncrieff et al. (2001) **417.** Hoppe & Svalastoga (1980)  **418.** Herrtage & Houlton (1979) **419.** Cerundolo et al. (2000) **420.** Freeman et al. (2013) **421.** Houlton & Herrtage (1980) **422.** Cameron et al. (2007) **423.** Rubin (1989) **424.** Cottrell & Barnett (1988) **425.** Caswell & Nykamp (2003) **426.** Hunter et al. (2007) **427.** André et al. (2008) **428.** Catchpole et al. (2013) **429.** Shelton (1999) **430.** Feldman & Nelson (1996) **431.** Goldschmidt & Shofer (1992) **432.** Gelatt & MacKay (2005) **433.** Case et al. (1992) **434.** Jansen & Arnesen (1990) **435.** Bjerkas & Narfstrom (1994) **436.** Ellison & Halling (2004) **437.** Miller et al. (1984) **438.** Bagley (2005) **439.** Giger (2003) **440.** Selby et al. (1981) **441.** Panciera (1994) **442.** Bryan et al. (2006) **443.** da Silva et al. (2012) **444.** Smith (2006) **445.** Brons et al. (2013)  **446.** Alam et al. (2007) **447.** Mortari et al. (2009) **448.** Baker-Gabb et al. (2003) **449.** Robins & Innes (2006) **450.** Scott et al. (2001a) **451.** Scott & Anderson (1991)  **452.**  Miller (2008) **453.** Lit et al. (2013) **454.** Chavkin et al. (1994) **455.** Zhou et al. (2010) **456.** Padgett et al. (1995) **457.** Egenvall et al. (2001) **458.** Kaswan & Salisbury (1990) **459.** Mills et al. (1985) **460.** Venter et al. (1996) **461.** Scott et al. (2001b) **462.** Cerundolo (1999) **463.** Capen & Martin (1975) **464.** Davis (1958) **465.** Bellenger et al. (1990) **466.** Bellenger (1980) **467.** Hayes & Fraumeni (1974) **468.** Noureddine et al. (2004) **469.** Grenn & Lindo (1969) **470.** Gutierrez-Quintana et al. (2014) **471.** Corcoran & Kock (1993) **472.** Paradis et al. (1989) **473.** Teske et al. (2002) **474.** Hargis et al. (1992) **475.** Wood et al. (2007) **476.** March et al. (2009) **477.** Evans & Adams (2010b) **478.** Morgan (1989) **479.** Engstrom (1966) **480.** Lothrop (1988) **481.** Witsberger et al. (2008) **482.** Schultheiss (2006) **483.** Edwards et al. (1989) **484.** Fox et al. (1999) **485.** Bussadori et al. (2001) **486.** Day (1994) **487.** Onions (1984) **488.** Bedford (1988) **489.** Post et al. (1991) **490.** Sueki et al. (1997) **491.** Vianna & Tobias (2005) **492.** Marmor (1982) **493.** Miller et al. (2004) **494.** Boudreaux et al. (1994) **495.** Palmer et al. (1984) **496.** Buchanan (1999) **497.** Hayes (1984) **498.** de Lahunta (1983) **499.** Brown et al. (1985) **500.** Murphy et al. (2001) **501.** Matic (1988) **502.** Patterson (1968) **503.** Smith & Knottenbelt (1988) **504.** Guilford et al. (1996) **505.** Niskanen & Thrusfield (1998) **506.** Borgarelli et al. (2006) **507.** Duval et al. (1999) **508.** Rudd et al. (1990) **509.** Ho et al. (2011) **510.** Kimura et al. (1993) **511.** Acland & Aguirre (1987) **512.** Holt & Thrusfield (1993) **513.** Aldrich et al. (1997) **514.** DeNovo (2003) **515.** D’Anna et al. (2007) **516.** Romagnoli (1991) **517.** Weaver (1983) **518.** Cummings et al. (1983) **519.** Morrison et al. (1987) **520.** Gurguis et al. (1990) **521.** van Tongern et al. (2000) **522.** Russell et al. (2006) **523.** Dukes-McEwan et al. (2003) **524.** Sevelius et al. (1994) **525.** Lawson (1973) **526.** Sanchez et al. (2007) **527.** Narfström et al. (2007) **528.** Watson et al. (1993)  **529.** Fall et al. (2007) **530.** Curtis et al. (1983) **531.** Spiess (1993) **532.** Heitmann et al. (2005).
