# Supplementary material for: The challenges of pedigree dog health: approaches to combating inherited disease
Source: Canine Genet Epidemiol. 2015 Feb 11;2:3. doi: 10.1186/s40575-015-0014-9 (PMC4579364; doi:10.1186/s40575-015-0014-9)
Supplement: Additional file 2: Table S2. — Inherited disorders in pedigree dogs. [file 40575_2015_14_MOESM2_ESM.docx]

**Table S2**. Inherited disorders in pedigree dogs.

Table organized in alpha order by affected organ system. Organ system, the primary organ system affected by the disorder ; Condition, the name of inherited disorder and common synonyms in brackets; Breed, the breeds affected by the disorder denoted by code (AT = Airedale Terrier, AUT = Australian Terrier, ACD = Australian Cattle Dog, AP = Affenpinscher, AH = Afghan Hound, ASD = Anatolian Shepherd Dog, AS = Australian Shepherd, AST = Australian Silky Terrier, AWS = American Water Spaniel, BJI = Basenji, BGVG = Basset Griffon Vendeen (Grand), BGVP = Basset Griffon Vendeen (Petit), BMH = Bavarian Mountain Hound, BC = Bearded Collie, BN = Beauceron, BT = Bedlington Terrier, BSM = Belgian Shepherd (Malanois), BST = Belgian Shepherd (Tervueren), BMD = Bernese Mountain Dog, BH = Bloodhound, BI = Borzoi, BOT = Boston Terrier, BDF = Bouvier des Flandres, BD = Briard, BY = Brittany, CD = Canaan Dog, CED = Canadian Eskimo Dog, CC = Chinese Crested, CCR = Curly Coated Retriever, CWCW = Chow Chow, CBS = Clumber Spaniel, CS = Collie (Smooth), CSD = Catalan Sheepdog, CDT = Coton de Tulear, CSA = Cocker Spaniel (American), CBR = Chesapeake Bay Retriever, DDT = Dandie Dinmont Terrier, DH = Deerhound, ES = English Setter, EMD = Estrela Mountain Dog, EBMD = Entlebucher Mountain Dog, FH = Foxhound, FL = Finnish Lapphund, FS = Finnish Spitz, FTS = Fox Terrier (Smooth), FTW = Fox Terrier (Wire), FB = French Bulldog, FSL = Field Spaniel, GB = Griffon Bruxellois, GBDG = Grand Bleu de Gacogne, GS = Giant Schnauzer, GST = Gordon Setter, GP = German Pinscher, GWP = German Wirehaired Pointer, GLP = German Longhaired Pointer, GIT = Glen of Imaal Terrier, GSMD = Greater Swiss Mountain Dog, GH = Greyhound, HE = Havanese, HK = Hungarian Kuvasz, HP = Hungarian Puli, HT = Hovawart, IH = Ibizan Hound, IGH = Italian Greyhound, IT = Irish Terrier, IW = Irish Wolfhound, IRWS = Irish Red & White Setter, IS = Italian Spinone, IWS = Irish Water Spaniel, JAI = Japanese Akita Inu, JC = Japanese Chin, JSI = Japanese Shiba Inu, JS = Japanese Spitz, KBT = Kerry Blue Terrier, KHE = Kooikerhondje, KCS = King Charles Spaniel, KH = Keeshond, KJ = Korean Jindo, LBR = Leonberger, LLD = Lowchen (Little Lion Dog), LH = Lancashire Heeler, LM = Large Munsterlander, LT = Lakeland Terrier, LR = Lagotto Romagnolo, MF = Mastiff, ME = Maltese, MT = Manchester Terrier, MP = Miniature Pinscher, MBT = Miniature Bull Terrier, MH = Mexican Hairless, NFL = Newfoundland, NEH = Norwegian Elkhound, NBH = Norwegian Buhund, NSDT = Nova Scotia Duck Tolling Retriever, NMF = Neapolitan Mastiff, NT = Norfolk Terrier, NWT = Norwich Terrier, OES = Old English Sheepdog, OH = Otterhound, PH = Pharoah Hound, PRT = Parson Russell Terrier, PWD = Portuguese Water Dog, PDO = Portuguese Podenco, PLS = Polish Lowland Sheepdog, PMD = Pyrenean Mountain Dog, PN = Papillon, POM = Pomeranian, PKE = Pekingese, PR = Pointer, PSD = Pyrenean Sheepdog, RBT = Russian Black Terrier, SCWT = Soft-Coated Wheaton Terrier, SL = Swedish Lapphund, SB = Saint Bernard, SD = Samoyed, SI = Sloughi, SKI = Saluki, SKE = Schipperke, ST = Sealyham Terrier, SKY = Skye Terrier, SS = Sussex Spaniel, SWD = Spanish Water Dog, SZR = Schnauzer, TS = Tibetan Spaniel, TM = Tibetan Mastiff, WCC = Welsh Corgi (Cardigan), WCP = Welsh Corgi (Pembroke), WSS = Welsh Springer Spaniel, WT = Welsh Terrier)**;** Inheritance, the mode of inheritance (if known); DNA test, DNA test developed and available for screening schemes; Related Disorders, any related or associated disorders**;** References, numbered with brief citation at the end the table (full citations in references). The web-based database ‘‘Inherited Diseases in Dogs’’ (Sargan, D. R. 2004. IDID: inherited diseases in dogs: web-based information for canine inherited disease genetics. *Mammalian Genome*, *15*(6), 503-506. http://www.vet.cam.ac.uk/idid) and Breed Predispositions to Disease in Dogs and Cats (Gough, A., Thomas, A. 2011. John Wiley & Sons) were used as resources.

(**NOTE**: This is not an exhaustive list of all inherited disorders in pedigree dogs and new DNA disease tests are continously being developed)

| **Organ system** | | | | | | **Condition** | | | | | | **Breed** | | **Group** | | | | **Inheritance** | **Age of onset** | **DNA test** | **Related disorders** | **References** |
| --- | --- | --- | --- | --- | --- | --- | --- | --- | --- | --- | --- | --- | --- | --- | --- | --- | --- | --- | --- | --- | --- | --- |
| Behavioural | | | | | | Interdog dominance aggression | | | | | | HT | | WORKING | | | | Unknown | - | No |  | 246 |
| Behavioural | | | | | | Aggressive temperament | | | | | | JSI | | UTILITY | | | | Unknown | - | No |  | 400 |
| Behavioural | | | | | | Nervousness | | | | | | PR | | GUNDOG | | | | Unknown (strong line inheritance) | - | - |  | 350, 520 |
| Behavioural | | | | | | Unpredictable behavioural changes | | | | | | BSM | | PASTORAL | | | | Unknown | - | No | Seizures | 453 |
| Cardiovascular | | | | | | Haemophilia (A and/or B) | | | | | | AT  AS  BJI  BY  CWCW  FTW  FB  GWP  PMD  ME  MT  OES  WCP  PWD  JSI  SB  ST  POM  CSA | | TERRIER  PASTORAL  HOUND  GUNDOG  UTILITY  TERRIER  UTILITY  GUNDOG  PASTORAL  TOY  TERRIER  PASTORAL  PASTORAL  WORKING  UTILITY  WORKING  TERRIER  TOY  GUNDOG | | | | X-linked recessive | -  -  -  -  -  -  -  -  -  -  -  -  -  -  -  -  -  -  - | Yes  -  -  -  -  -  -  -  -  -  -  -  -  -  -  -  -  Yes  - |  | 30, 31, 192, 222, 319, 351, 411 |
| Cardiovascular | | | | | | von Williebrand disease (Type 1, 2, and 3) | | | | | | AT  BMD  GH  IW  KBT  GWP  KHE  MT  PN  WCP  SZR  CBR | | TERRIER  WORKING  HOUND  HOUND  TERRIER  GUNDOG  GUNDOG  TERRIER  TOY  PASTORAL  UTILITY  GUNDOG | | | | Unknown  Autosomal recessive  Unknown  Auto. rec. (suggested)  Autosomal recessive  Autosomal recessive  Autosomal recessive  Auto. rec. (suggested)  Unknown  Autosomal dominant  Unknown  Autosomal recessive | -  -  -  -  -  -  -  -  -  -  -  - | -  Yes  -  -  Yes  Yes  Yes  Yes  Yes  Yes  -  - |  | 30, 32, 114, 205, 274, 365 |
| Cardiovascular | | | | | | Factor I (Fibrinogen) deficiency (hypofibrinogenemia) | | | | | | BMD  BI  SB | | WORKING  HOUND  WORKING | | | | Auto. rec. (probable)  Auto. rec. (probable)  Familial, unknown | -  -  - | -  -  - |  | 30, 107, 375 |
| Cardiovascular | | | | | | Factor VII (plasma protein) deficiency | | | | | | BMD | | WORKING | | | | Auto. rec. (probable) | - | - |  | 30 |
| Cardiovascular | | | | | | Factor XI (Plasma thrombo-plastin antecedent) | | | | | | PMD  KBT | | PASTORAL  TERRIER | | | | Autosomal recessive  Autosomal recessive | -  - | -  Yes |  | 30, 225, 267 |
| Cardiovascular | | | | | | Factor X (Stuart-Prower Factor) deficiency | | | | | | CSA | | GUNDOG | | | | Autosomal dominant | <2 weeks | - |  | 30 |
| Cardiovascular | | | | | | High K erythrocytes | | | | | | KJ  JSI | | UTILITY  UTILITY | | | | Autosomal recessive  Autosomal recessive | -  - | -  - |  | 275, 397 |
| Cardiovascular | | | | | | Stomatocytosis | | | | | | SZR | | UTILITY | | | | Familial | - | No | Anemia | 391 |
| Cardiovascular | | | | | | Hyperlipidaemia | | | | | | BY | | GUNDOG | | | | Familial | - | - |  | 143 |
| Cardiovascular | | | | | | Postoperative haemorrhage | | | | | | GSMD | | WORKING | | | | Familial | - | No |  | 228 |
| Cardiovascular | | | | | | Wolman disease  (Lipid storage disease) | | | | | | FTS  FTW | | TERRIER  TERRIER | | | | Unknown  Unknown | -  - | -  - | Intestinal mal absorption | 184 |
| Cardiovascular | | | | | | Patent ductus arteriosus | | | | | | WCC  KH  ME  AS  WCP  POM  SKI  CSA | | PASTORAL  UTILITY  TOY  PASTORAL  PASTORAL  TOY  HOUND  GUNDOG | | | | Unknown  Polygenic  Breed at increased risk  Breed at increased risk  Familial, unknown  Unknown  Familial, unknown  Unknown | -  -  -  -  -  -  -  - | -  -  -  -  -  -  -  - | Heart failure | 149, 199, 264, 339, 352, 378, 501 |
| Cardiovascular | | | | | | Peripheral artery occlusive disease (femoral) | | | | | | KCS | | TOY | | | | Unknown | - | - |  | 271 |
| Cardiovascular | | | | | | Pharmacogenetic abnormality | | | | | | GS | | WORKING | | | | Unknown | - | - |  | 209 |
| Cardiovascular | | | | | | Sick sinus syndrome  (Sinus node disease) | | | | | | CSA | | GUNDOG | | | | Familial, unknown | 7-10 years | - | Fibrosis of sinus node | 412 |
| Cardiovascular | | | | | | Dilated cardiomyopathy (DCM) | | | | | | EMD  IW  SB  NFL  OES  DH  NMF  PWD  CSA  AT | | PASTORAL  HOUND  WORKING  WORKING  PASTORAL  HOUND  WORKING  WORKING  GUNDOG  TERRIER | | | | Unknown  Unknown  Unknown  Unknown  Breed at increased risk  Unknown  Unknown  Autosomal recessive  Unknown  Familial (suspected) | -  Adult  5 years  5 years  -  -  5 years  2-30 weeks  -  - | No  No  No  No  No  No  No  Yes  No  No | Other heart disease, atrial fibrillation, congestive heart failure | 179, 255, 352, 358, 408, 506, 523 |
| Cardiovascular | | | | | | Glanzmann’s Type I thrombasthenia | | | | | | PMD  OH | | PASTORAL  HOUND | | | | Unknown  Autosomal recessive | -  - | -  Yes |  | 221, 330 |
| Cardiovascular | | | | | | Spitz dog thrombopathia | | | | | | FS | | HOUND | | | | Unknown | - | Yes |  | 494 |
| Cardiovascular | | | | | | Canine leukocyte adhesion deficiency (CLAD) | | | | | | IRWS | | GUNDOG | | | | Unknown | - | Yes |  | 251 |
| Cardiovascular | | | | | | Thrombocytopenia  (immune mediated) | | | | | | OES | | PASTORAL | | | | Unknown | - | - | Clotting problems | 323 |
| Cardiovascular | | | | | | Thrombopathy  (Delta storage pool defect) | | | | | | CSA | | GUNDOG | | | | Familial, unknown | - | - |  | 414 |
| Cardiovascular | | | | | | Persistent right aortic arch | | | | | | GP  GH | | WORKING  HOUND | | | | Familial  Familial | -  - | No |  | 204, 236 |
| Cardiovascular | | | | | | Aortic aneurysm | | | | | | LBR | | WORKING | | | | Familial | - | - | Twisting ascending aorta, dilation of thoracic arteries | 285 |
| Cardiovascular | | | | | | Conotruncal defect  (Cardiac malformation) | | | | | | KH | | UTILITY | | | | Autosomal recessive | - | - |  | 261 |
| Cardiovascular | | | | | | Ventricular septal defect | | | | | | GP | | WORKING | | | | Unknown | Neonatal | No |  | 199 |
| Cardiovascular | | | | | | Atrial septal defect | | | | | | SD | | PASTORAL | | | | Familial, unknown | - | - |  | 387 |
| Cardiovascular | | | | | | Cardiac valvular defects | | | | | | CSA | | GUNDOG | | | | Unknown | - | - | Heart murmur | 158 |
| Cardiovascular | | | | | | Mitral valve disease  (left atrio-v, myxomatous, chronic valvular) | | | | | | KCS  ME  PKE | | TOY  TOY  TOY | | | | Polygenic  Breed at increased risk  Breed at increased risk | 5 years  -  - | -  No  - | Heart murmur | 270, 293 |
| Cardiovascular | | | | | | Tricuspid valve dysplasia (right atrio-ventricular) | | | | | | MF  PMD | | WORKING  PASTORAL | | | | Unknown  Breed at increased risk | -  - | -  - |  | 158, 496 |
| Cradiovascular | | | | | | Mitral stenosis | | | | | | NFL | | WORKING | | | | Autosomal dominant | - | - | Heart failure | 305 |
| Cardiovascular | | | | | | Pulmonic stenosis | | | | | | CWCW  FTS  FTW  FB  SD  SZR  MF | | UTILITY  TERRIER  TERRIER  UTILITY  PASTORAL  UTILITY  WORKING | | | | Unknown  Polygenic (suspected)  Unknown  Unknown  Polygenic (suspected)  Unknown  Polygenic (suggested) | -  -  -  -  -  -  - | -  -  -  -  -  No  - | Abnormal cardiac rhythms and/or abnormalities, congestive heart failure | 158, 189, 199, 384, 484, 485 |
| Cardiovascular | | | | | | Subaortic stenosis | | | | | | NFL | | WORKING | | | | Autosomal dominant | 3-12 months | - | Heart murmur | 300 |
| Cardiovascular | | | | | | Tetralogy of Fallot | | | | | | FTW  KH | | TERRIER  UTILITY | | | | Congenital, rare  Breed at increased risk | -  - | -  - |  | 496, 502 |
| Cardiovascular | | | | | | Macrothrobocytopenia | | | | | | NT | | TERRIER | | | | Familial, unknown | 10-12 months | No |  | 311 |
| Endocrine | | | | | | Portosystemic shunt | | | | | | ACD  IW  DDT  HE  ME  OES  DH  AST | | PASTORAL  HOUND  TERRIER  TOY  TOY  PASTORAL  HOUND  TOY | | | | Breed at increased risk  Unknown  Breed at increased risk  Breed at increased risk  Breed at increased risk  Unknown  Polygenic (suspected)  Breed at increased risk | < 1 year  -  < 1 year  < 1 year  < 1 year  < 1 year  < 1 year  < 1 year | No | Hepatic encephalitis | 44-46, 171, 327 |
| Endocrine | | | | | | Hypoadrenocorticism (Addison’s disease) | | | | | | BC  PWD  LBR  SCWT  NSDT | | PASTORAL  WORKING  WORKING  TERRIER  GUNDOG | | | | Unknown  Autosomal recessive  Familial  Unknown  Autosomal recessive | 4 years  4-5 years  -  4 years  2-3 years | -  -  -  -  No | Azotemia, hyperphosphatemia, hyperkalemia | 93, 318, 283, 360, 404, 430 |
| Endocrine | | | | | | Hyperadrenocorticism  (Cushing’s-like syndrome)  (Pituitary dependent and non) | | | | | | CWCW  DDT  POM  SD  BOT  AS | | UTILITY  TERRIER  TOY  PASTORAL  UTILITY  PASTORAL | | | | Unknown  Familial  Unknown  Unknown  Breed at increased risk  Breed at increased risk | -  10 years  -  -  10 years  10 years | -  -  -  -  -  - | Partial adrenal dysfunction, alopecia (alopecia X) | 162, 169, 355, 386, 463, 475 |
| Endocrine | | | | | | Copper toxicosis | | | | | | BT  SKY | | TERRIER  TERRIER | | | | Autosomal recessive  Familial, unknown | 12 months  - | Yes  - | Anorexia, seizures, portosystemic shunt | 97, 98, 401 |
| Endocrine | | | | | | Sodium thiopental sensitivity  (Thiopental pharmacokinetics) | | | | | | GH  IGH | | HOUND  TOY | | | | Unknown  Breed at increased risk | -  - | No |  | 238 |
| Endocrine | | | | | | Anaesthesia idiosyncrasies | | | | | | GH | | HOUND | | | | Unknown | - | No |  | 242 |
| Endocrine | | | | | | Diabetes mellitus | | | | | | KH  SD  SL  FS  HP  MP  AUT | | UTILITY  PASTORAL  PASTORAL  HOUND  PASTORAL  TOY  TERRIER | | | | Autosomal recessive  Familial  Familial  Breed at increased risk  Breed at increased risk  Breed at increased risk  Familial | <6 months  7-9 years  7 years  -  7-9 years  4-14 years  4-7 years | -  -  -  -  -  -  - | Hyperglycaemia, glucosuria | 380, 381, 428, 430, 492, 529 |
| Endocrine | | | | | | Lymphocytic thyroiditis (primary hypothyroidism) | | | | | | BI  ES  GS  GH  HT  AT  POM  AH  PR  SKY | | HOUND  GUNDOG  WORKING  HOUND  WORKING  TERRIER  TOY  HOUND  GUNDOG  TERRIER | | | | Autosomal recessive  Unknown  Breed at increased risk  Unknown  Breed at increased risk  Breed at increased risk  Breed at increased risk  Breed at increased risk  Breed at increased risk  Breed at increased risk | 2 years  -  2-3 years  -  3-7 years  2-3 years  2-3 years  2-3 years  -  - | -  -  -  -  No  -  -  -  -  - | Obesity, hair loss | 124, 176, 207, 212, 239, 247 |
| Endocrine | | | | | | Hyperparathyroidism  (Primary, PHPT) | | | | | | KH | | UTILITY | | | | Autosomal dominant | >7 years | Yes | Hypercalcaemia, parathyroid adenoma | 263 |
| Endocrine | | | | | | Hypoparathyroidism  (Primary) | | | | | | SB | | WORKING | | | | Breed at increased risk | Any age | - |  | 522 |
| Endocrine | | | | | | Growth hormone-responsive dermatosis | | | | | | CWCW  KH  POM  SD  AWS | | UTILITY  UTILITY  TOY  PASTORAL  GUNDOG | | | | Unknown  Breed at increased risk  Breed at increased risk  Breed at increased risk  Breed at increased risk | 1-2 years | -  -  -  -  - |  | 480 |
| Gastrointestinal | | | | | | Ivermectin sensitivity | | | | | | AS  LT  OES | | PASTORAL  TERRIER  PASTORAL | | | | Autosomal recessive  Unknown  Autosomal recessive | -  -  - | Yes  No  No | Gastrointestinal tract toxicosis, blindness, gait deficit (hind limb ataxia) | 69, 70, 276, 329 |
| Gastrointestinal | | | | | | Immunoproliferative enteropathy | | | | | | BJI | | HOUND | | | | Familial, presumed autoimmune | <3 years | - |  | 76-78 |
| Gastrointestinal | | | | | | Gastric carcinoma | | | | | | BST | | PASTORAL | | | | Unknown | 9 years | No |  | 104 |
| Gastrointestinal | | | | | | Congenital megaoesophagus | | | | | | FTW  FTS | | TERRIER  TERRIER | | | | Autosomal recessive | -  - | -  - | Myasthenia gravis | 185 |
| Gastrointestinal | | | | | | Exocrine pancreatic insufficiency | | | | | | CWCW  ES | | UTILITY  GUNDOG | | | | Unknown  Familial | -  Juvenile | No | Diabetes | 164, 174 |
| Gastrointestinal | | | | | | Taurine deficiency | | | | | | NFL | | WORKING | | | | Familial | - | - | Cardiomyopathy | 306 |
| Gastrointestinal | | | | | | Ulcerative colitis (Histiocytic inflammatory bowel disease) | | | | | | FB | | UTILITY | | | | Unknown | - | - | Brachycephalic respiratory syndrome | 194 |
| Gastrointestinal | | | | | | Haemorrgagic gastroenteritis | | | | | | KCS  PKE | | TOY  TOY | | | | Breed at increased risk  Breed at increased risk | > 5 years  5-7 years | -  - |  | 504 |
| Gastrointestinal | | | | | | Chronic hepatitis | | | | | | CSA | | GUNDOG | | | | Breed at increased risk | - | - |  | 524 |
| Gastrointestinal | | | | | | Protein losing enteropathy (PLE) | | | | | | SCWT | | TERRIER | | | | Familial, unknown | 4-6 years | - | Inflammatory bowel disease | 406 |
| Gastrointestinal | | | | | | Pancreatic acinar atrophy | | | | | | GH | | HOUND | | | | Familial | - | No |  | 240 |
| Gastrointestinal | | | | | | Antral pyloric hypertrophy (Pyloric stenosis) | | | | | | BOT  ME  PKE | | UTILITY  TOY  TOY | | | | Congenital  Congenital  Breed at increased risk | 6-12 months  -  Adult | -  -  - |  | 464, 465, 514 |
| Gastrointestinal | | | | | | Gastric dilation (volvulus) | | | | | | BH  BI  GST  IW  SB  NMF  OH  GBDG  GLP | | HOUND  HOUND  GUNDOG  HOUND  WORKING  WORKING  HOUND  HOUND  GUNDOG | | | | Familial, unknown  Breed at increased risk  Familial, unknown  Familial, unknown  Breed at increased risk  Breed at increased risk  Breed at increased risk  Breed at increased risk  Breed at increased risk | Adult-elderly | -  -  -  -  -  No  No  -  - |  | 121, 215 |
| Gastrointestinal | | | | | | Imerslund-Grasbeck syndrome  (Cobalamin malabsorption) | | | | | | GS  AS | | WORKING  PASTORAL | | | | Autosomal recessive  - | 6-12 weeks  - | Yes  Yes |  | 207, 439 |
| Gastrointestinal | | | | | | Cleft palate | | | | | | BOT  BY  PSD | | UTILITY  GUNDOG  PASTORAL | | | | Autosomal recessive | Birth | No |  | 127, 140, 363 |
| Immune | | | | | | Complement (C3) deficiency | | | | | | BY | | GUNDOG | | | | Autosomal recessive | - | - | Spinal muscular atrophy | 141 |
| Immune | | | | | | Severe combined immunodeficiency (SCID) | | | | | | WCC | | PASTORAL | | | | X-linked recessive | - | Yes |  | 148 |
| Immune | | | | | | Autoimmune disorders (multiple involving glandular tissues) | | | | | | IGH | | TOY | | | | Increased risk in case series | Adult | No | Adrenals, thyroid, pancreas, gonads and skin affected | 243 |
| Immune | | | | | | Haemolytic anaemia  (auto-immune, AIHA; immune-mediated, IMHA) | | | | | | CSA  FS  OES  MP | | GUNDOG  HOUND  PASTORAL  TOY | | | | Unknown  Breed at increased risk  Breed at increased risk  Unknown | Adult  Adult  5-6 years  1-7 years | No | Bilirubinaemia, other autoimmune disease(s) | 416, 459, 493 |
| Integument | | | | | | Alopecia  (Alopecia X, colour dilution alopecia, seasonal flank alopecia) | | | | | | AP  AT  CWCW  GP  KH  MP  POM  CBR  CCR  SD  SZR  IWS  BMD  GH  NFL  SKI  SKE  AST | | TOY  TERRIER  UTILITY  WORKING  UTILITY  TOY  TOY  GUNDOG  GUNDOG  PASTORAL  UTILITY  GUNDOG  WORKING  HOUND  WORKING  HOUND  UTILITY  TOY | | | | Familial  Breed at increased risk  X-linked (suggested)  Unknown  X-linked (suggested)  Unknown  X-linked (suggested)  Unknown  Familial, unknown  X-linked (suggested)  Unknown  Autosomal dominant  -  -  Unknown  -  -  - | 2-5 years  2-4 years  <1 year  6 mo-2 years  <1 year  6 mo-2 years  -  5 mo-4 years  1 year  -  6 mo-2 years  2-5 years  -  -  18 months  -  -  - | No  -  -  -  -  -  No  No  -  -  No  -  -  -  -  -  -  - | Hyperadrenocorticism | 2, 26, 162, 203, 266, 356, 366, 368, 419, 462 |
| Integument | | | | | | Follicular dysplasia  (including Black hair) | | | | | | BC  GST  LM  SKI  CCR  IWS  PWD  PN  PR  SKE  CSA | | PASTORAL  GUNDOG  GUNDOG  HOUND  GUNDOG  GUNDOG  WORKING  TOY  GUNDOG  UTILITY  GUNDOG | | | | Autosomal recessive  Familial  Auto. rec. (suspected)  Auto. rec. (suspected)  Unknown  -  Unknown  Familial  Familial  Auto. rec. (suspected)  Familial | 4 weeks  -  2-4 years  4 weeks  2-4 years  -  2-5 years  < 1 year  1-2 years  < 1 year  < 1 month | -  -  Yes  -  -  -  -  -  -  -  - | Deafness, onychodystrophy, SLO | 90, 91, 94, 282, 379, 462, 450 |
| Integument | | | | | | Pattern baldness | | | | | | BOT  GH  IGH  MT  PWD  AWS | | UTILITY  HOUND  TOY  TERRIER  WORKING  GUNDOG | | | | Unknown | 6 months  6 months  6 months  6 months  6 months  6 months | -  -  -  -  -  - |  | 450, 461, 462 |
| Integument | | | | | | Sebaceous adenitis | | | | | | JAI  SD | | UTILITY  PASTORAL | | | | Unknown | -  - | -  - |  | 462 |
| Integument | | | | | | Familial canine dermatomyositis | | | | | | BN  CWCW  HK  LT | | WORKING  UTILITY  PASTORAL  TERRIER | | | | Familial, unknown | < 6 months  -  -  - | -  -  -  - |  | 450, 462 |
| Integument | | | | | | Ectodermal dysplasia (hairlessness) | | | | | | CC | | TOY | | | | Dominant polygenic | Birth | - |  | 150 |
| Integument | | | | | | Zinc responsive dermatosis | | | | | | PH | | HOUND | | | | Unknown | - | No |  | 344 |
| Integument | | | | | | Vogt-Koyanagi-Harada-like Syndrome | | | | | | CWCW  SD | | UTILITY  PASTORAL | | | | Breed at increased risk | -  1-5 years | -  - |  | 478 |
| Integument | | | | | | Tyrosinase deficiency | | | | | | CWCW | | UTILITY | | | | Unknown | Puppy | - |  | 479 |
| Integument | | | | | | Neoplasia  (Cutaneous & oral melanoma, cutaneous lymphoma & histiocytoma, cutaneous haemangioma, renal neoplasia, haemangiosarcoma,  fibrosarcoma, histiocytic sarcoma, squamous cell carcinoma, sweat gland tumor, testicular neoplasia) | | | | | | AT  BN  BMD  BOT  CWCW  GH  SZR  GST  AST  BY  CSA  OES  BC  BH  BD  MF  FTS  FTW  GS  BC  PMD  IT  IW  IGH  MT  NEH  PKE  CBR  SCWT  AUT | | TERRIER  PASTORAL  WORKING  UTILITY  UTILITY  HOUND  UTILITY  GUNDOG  TOY  GUNDOG  GUNDOG  PASTORAL  PASTORAL  HOUND  PASTORAL  WORKING  TERRIER  TERRIER  WORKING  PASTORAL  PASTORAL  TERRIER  HOUND  TOY  TERRIER  HOUND  TOY  GUNDOG  TERRIER  TERRIER | | | | Breed at increased risk  Breed at increased risk  Breed at increased risk  Breed at increased risk  Breed at increased risk  Breed at increased risk  Unknown  Breed at increased risk  -  Breed at increased risk  -  Breed at increased risk  Breed at increased risk  Breed at increased risk  Breed at increased risk  Breed at increased risk  Breed at increased risk  Breed at increased risk  Breed at increased risk  Breed at increased risk  Breed at increased risk  Breed at increased risk  Breed at increased risk  Breed at increased risk  Breed at increased risk  Breed at increased risk  Breed at increased risk  Breed at increased risk  Breed at increased risk  Breed at increased risk | -  -  -  8 years  8-10 years  3 years  -  8 years  8 years  8-9 years  -  8-10 years  8 years  8 years  9 years  3 years  8 years  8 years  Adult  8 years  9 years  9 years  9 years  9 years  9 years  10 years  9-11 years  9 years  8 years  8 years | No |  | 33, 111, 129, 157, 361, 392, 431, 442, 472, 474, 482, 487, 499, 517 |
| Integument | | | | | | Chemodectoma | | | | | | BOT | | UTILITY | | | | Breed at increased risk | Elderly | - |  | 467 |
| Integument | | | | | | Malignant Pilomatricoma | | | | | | AT  KBT  OES  SCWT  BDF  SZR | | TERRIER  TERRIER  PASTORAL  TERRIER  WORKING  UTILITY | | | | Increased risk in case series | 6 years  -  -  -  -  - | -  -  -  -  -  - |  | 443 |
| Integument | | | | | | Histiocytosis | | | | | | BMD | | WORKING | | | | Polygenic (suggested) | 3-4 years | - |  | 456 |
| Integument | | | | | | Mast cell tumor | | | | | | ACD  BOT  ES  FTS | | PASTORAL  UTILITY  GUNDOG  TERRIER | | | | Breed at increased risk | 4 months  -  4 months  4 months | -  -  -  - |  | 33, 95, 431, 448 |
| Integument | | | | | | Chronic superficial keratitis (pannus) | | | | | | BST  GH  AS | | PASTORAL  HOUND  PASTORAL | | | | Breed at increased risk  Auto. rec. (suspected)  Breed at increased risk | 4-7 years  2 years  4-7 years | -  -  - |  | 229, 454 |
| Integument | | | | | | Trichoblastoma  (basal cell tumors) | | | | | | BC  FS  HP  KBT  SCWT  WSS | | PASTORAL  HOUND  PASTORAL  TERRIER  TERRIER  GUNDOG | | | | Breed at increased risk | 7 years  8 years  > 5 years  5-7 years  7-9 years  > 5 years | -  -  -  -  -  - |  | 431 |
| Integument | | | | | | Perianal (hepatoid) gland adenoma | | | | | | BY  PKE  SD | | GUNDOG  TOY  PASTORAL | | | | Breed at increased risk | 10 years | -  -  - |  | 431 |
| Integument | | | | | | Pemphigus foliaceus | | | | | | BC  CWCW  NFL  SKE  FS | | PASTORAL  UTILITY  WORKING  UTILITY  HOUND | | | | Breed at increased risk  Unknown  Breed at increased risk  Breed at increased risk  Breed at increased risk | 4 years  -  3 months  3 months  - | -  -  -  -  - | Alopecia, peripheral collarettes, footpad lesions, cracking, dermatitis | 92, 159 |
| Integument | | | | | | Familial chronic pemphigus (epidermal acantholysis) | | | | | | ES | | GUNDOG | | | | Autosomal dominant | - | - |  | 490 |
| Integument | | | | | | Pyoderma/furunculosis  (Muzzle folliculitis) | | | | | | MF | | WORKING | | | | Breed at increased risk | - | - |  | 486 |
| Integument | | | | | | Palmoplantar hyperkeratosis | | | | | | IT | | TERRIER | | | | Autosomal recessive | 6 months | - |  | 254 |
| Integument | | | | | | Seborrhoea | | | | | | CSA | | GUNDOG | | | | Auto. rec. (probable) | 1 year | - | Secondary bacterial infections | 415, 450 |
| Integument | | | | | | Symmetrical lupoid onychodystrophy (SLO) | | | | | | BC  GS  GST | | PASTORAL  WORKING  GUNDOG | | | | Familial | -  -  - | No | Autoimmune dermatitis, alopecia, black hair follicular dysplasia | 94, 217 |
| Integument | | | | | | Histoplasmosis | | | | | | BY | | GUNDOG | | | | Breed at increased risk | < 2 years | - |  | 440 |
| Integument | | | | | | Epidermolysis bullosa  (dystrophic & junctional) | | | | | | BN | | WORKING | | | | Autosomal recessive | - | - | Crusted papules, erosions in genital region | 95, 450 |
| Integument | | | | | | Vitiligo | | | | | | BST | | PASTORAL | | | | Unknown | Juvenile | - | Autoimmune disorders | 103 |
| Integument | | | | | | Multiple cancer syndrome | | | | | | ES | | GUNDOG | | | | Unknown | - | No |  | 177 |
| Integument | | | | | | Dermoid sinus | | | | | | CWCW | | UTILITY | | | | Unknown | - | - |  | 160 |
| Integument | | | | | | Ocular dermoid cysts | | | | | | SB | | WORKING | | | | Unknown | - | No |  | 265, 373, 423 |
| Integument | | | | | | Demodex injai infestation | | | | | | FTW | | TERRIER | | | | Familial | - | - | Hypothyroidism | 190 |
| Integument | | | | | | Cutaneous vasculopathy | | | | | | GH | | HOUND | | | | Unknown | - | - |  | 234 |
| Integument | | | | | | Epidermal hyperkeratosis  (Keratinization defect) | | | | | | NT | | TERRIER | | | | Autosomal recessive | Birth | - |  | 310 |
| Integument | | | | | | Ectopic cilia | | | | | | PKE | | TOY | | | | Breed at increased risk | - | - |  | 265, 515 |
| Integument | | | | | | Entropion  (and/or Ectropion) | | | | | | BH  AT  BT  BMD  BDF  CWCW  MF  ES  FB  PMD  IW  KBT  LBR  NFL  NEH  PKE  POM  CBR  CCR  SB  CSA  CBS  WSS  MBT  JC | | HOUND  TERRIER  TERRIER  WORKING  WORKING  UTILITY  WORKING  GUNDOG  UTILITY  PASTORAL  HOUND  TERRIER  WORKING  WORKING  HOUND  TOY  TOY  GUNDOG  GUNDOG  WORKING  GUNDOG  GUNDOG  GUNDOG  TERRIER  TOY | | | | Polygenic (suspected) | -  -  -  -  -  2-6 weeks  -  -  -  -  -  -  -  -  -  -  -  -  -  -  -  -  -  -  - | No  -  -  -  -  -  -  -  -  -  -  -  -  -  -  -  -  -  -  -  -  -  -  -  - | Ectropin, damage to conjunctiva, macroblepharon | 123, 423, 265, 488 |
| Integument | | | | | | Trichiasis (caruncular) | | | | | | BOT | | UTILITY | | | | Breed at increased risk | - | - |  | 423 |
| Integument | | | | | | Distichiasis | | | | | | FB  HE  PKE  CSA | | UTILITY  TOY  TOY  GUNDOG | | | | Breed at increased risk | -  -  -  - | -  -  -  - | Entropion | 265, 423, 525 |
| Integument | | | | | | Cornifying epithelioma  (keratoacanthoma) | | | | | | NEH  BC  BT  KH  KBT  PKE  SD  SCWT | | HOUND  PASTORAL  TERRIER  UTILITY  TERRIER  TOY  PASTORAL  TERRIER | | | | Breed at increased risk | 7 years | -  -  -  -  -  -  -  - |  | 313, 431, 451, 503 |
| Musculoskeletal | | | | | | Legg-Calve Perthe disease | | | | | | AP  AS  MT  MP | | TOY  PASTORAL  TERRIER  TOY | | | | Increased risk in case series (familial)  Autosomal recessive  Unknown | 4-12 months  4-12 months  4-12 months  - | -  -  -  - |  | 3, 64, 163, 294 |
| Musculoskeletal | | | | | | Elbow deformity  (elbow incongruity) | | | | | | AH  NFL  SKY | | HOUND  WORKING  TERRIER | | | | Familial  Unknown  Autosomal recessive | -  -  - | -  -  - | Ununited anconal processes | 23, 303, 402 |
| Musculoskeletal | | | | | | Congenital elbow luxation | | | | | | BC  BOT  OES  PKE  MP  POM | | PASTORAL  UTILITY  PASTORAL  TOY  TOY  TOY | | | | Unknown  Unknown, uncommon | 4-5 months  < 3 months  4-5 months  4-5 months  < 3 months  4-5 months | -  -  -  -  -  - |  | 449 |
| Musculoskeletal | | | | | | Inherited polyneuropathy | | | | | | GH  LBR | | HOUND  WORKING | | | | Autosomal recessive  X-linked recessive | -  1-9 years | Yes  No | Ataxia, exercise intolerance, distal limb atrophy, dyspnea | 241, 284 |
| Musculoskeletal | | | | | | Ankyloglossia  (tied tongue) | | | | | | ASD | | PASTORAL | | | | Familial, X-linked | - | - |  | 39 |
| Musculoskeletal | | | | | | GM1 gangliosidosis | | | | | | PWD  JSI | | WORKING  UTILITY | | | | Autosomal recessive  Autosomal recessive | 2 months  5 months | Yes | Neurological impairment, ataxia | 359, 398 |
| Musculoskeletal | | | | | | Myasthenia gravis | | | | | | FTS  FTW  NFL | | TERRIER  TERRIER  WORKING | | | | Autosomal recessive  Autosomal recessive  Familial, unknown | 5 weeks  5 weeks  5-8 weeks | -  -  - | Muscular weakness | 185, 307, 437 |
| Musculoskeletal | | | | | | Mitochondrial myopathy | | | | | | OES  SS  CBS | | PASTORAL  GUNDOG  GUNDOG | | | | Unknown  Unknown, rare  Unknown, rare | -  -  - | -  -  - |  | 324, 421, 429 |
| Musculoskeletal | | | | | | Nemaline rod myopathy | | | | | | SKE | | UTILITY | | | | Unknown | Adult | - | Exercise intolerance | 389 |
| Musculoskeletal | | | | | | Elbow dysplasia | | | | | | ACD  BSM  BMD  BH  CWCW  ES  GST  GSMD  IW  MF  TM  NFL  SB  BDF  POM | | PASTORAL  PASTORAL  WORKING  HOUND  UTILITY  GUNDOG  GUNDOG  WORKING  HOUND  WORKING  WORKING  WORKING  WORKING  WORKING  TOY | | | | Polygenic  -  Males higher risk  -  Polygenic  Polygenic  Polygenic  Polygenic  Polygenic  Polygenic  Polygenic  Breed at increased risk  Polygenic  Breed at increased risk  Unknown | -  -  -  -  -  -  -  -  -  -  -  -  -  -  - | -  -  -  -  -  -  -  -  -  -  -  -  -  -  - | Osteochondrosis dissecans | 47, 64, 105, 153, 163, 304, 449 |
| Musculoskeletal | | | | | | Hip Dysplasia | | | | | | BMD  BH  BDF  BD  BY  CWCW  ES  EMD  GS  JAI  GST  SD  HK  AT  BS  CBR  GWP  PMD  KH  MF  NMF  NFL  NEH  OES  PWD  SB  HP  LBR  IW  OH  CBS  SZR  IWS  SS  WSS  BC | | WORKING  HOUND  WORKING  PASTORAL  GUNDOG  UTILITY  GUNDOG  PASTORAL  WORKING  UTILITY  GUNDOG  PASTORAL  PASTORAL  TERRIER  PASTORAL  GUNDOG  GUNDOG  PASTORAL  UTILITY  WORKING  WORKING  WORKING  HOUND  PASTORAL  WORKING  WORKING  PASTORAL  WORKING  HOUND  HOUND  GUNDOG  UTILITY  GUNDOG  GUNDOG  GUNDOG  PASTORAL | | | | Polygenic  Unknown  Unknown  Unknown  Unknown  Unknown  Unknown  Unknown  Unknown  Unknown  Unknown  Unknown  Unknown  Unknown  Unknown  Unknown  Unknown  Unknown  Unknown  Unknown  Unknown  Unknown  Unknown  Unknown  Unknown  Unknown  Unknown  Unknown  Unknown  Unknown  Unknown  Unknown  Unknown  Unknown  Unknown  Unknown | -  -  -  -  -  -  -  -  -  -  -  -  -  -  -  -  -  -  -  -  -  12-18 months  -  -  -  -  -  12-18 months  12-18 months  -  -  -  -  -  -  - | No | Gait problems, exercise intolerance | 64, 122, 134, 163, 178, 224, 206, 249, 288, 331, 420, 455, 481 |
| Musculoskeletal | | | | | | Oculo-skeletal dysplasia (short-limbed dwarfism) | | | | | | SD | | PASTORAL | | | | Autosomal recessive | - | Yes | Elbow and carpel deformity, cataracts, retinal detachment | 383 |
| Musculoskeletal | | | | | | Panosteitis  (Enostosis, eosinophilic) | | | | | | CWCW  MF  AH  BMD  CBR  ES  GS  PMD  IW  NMF  NFL  SB | | UTILITY  WORKING  HOUND  WORKING  GUNDOG  GUNDOG  WORKING  PASTORAL  HOUND  WORKING  WORKING  WORKING | | | | Breed at increased risk | Juvenile | -  -  -  -  -  -  -  -  -  -  -  - |  | 64 |
| Musculoskeletal | | | | | | Temporomandibular joint dystrophy | | | | | | CSA | | GUNDOG | | | | Unknown | 6 months | No |  | 417 |
| Musculoskeletal | | | | | | Myotonic myopathy  (Myotonia) | | | | | | ACD  CWCW | | PASTORAL  UTILITY | | | | Autosomal recessive | -  12 weeks | Yes  - | Abnormalities in skull development and dentition | 56, 57, 156 |
| Musculoskeletal | | | | | | Polydactyly  (skeletal malformations) | | | | | | AS  BMD  PMD  SB | | PASTORAL  WORKING  PASTORAL  WORKING | | | | X-linked (suggested)  Unknown  Autosomal dominant  Autosomal recessive | Birth | -  -  -  - | Syndactyly, brachygnatism, cleft palate and lip, anotia, extra thoracic vertebra, rib | 66, 67, 113, 224, 374 |
| Musculoskeletal | | | | | | Cranial cruciate ligament rupture | | | | | | BMD  NFL  CWCW  MF  NMF  CBR  SB | | WORKING  WORKING  UTILITY  WORKING  WORKING  GUNDOG  WORKING | | | | Unknown  Unknown  Unknown  Breed at increased risk  Unknown  Breed at increased risk  Unknown | -  >2 years  Elderly  Elderly  -  > 1 year  Elderly | No |  | 116, 308, 481, 507 |
| Musculoskeletal | | | | | | Pectus excavatum | | | | | | WT | | TERRIER | | | | Unknown | - | - |  | 436 |
| Musculoskeletal | | | | | | Patellar luxation  (including medical patellar luxation, MPL) | | | | | | CWCW  POM  ACD  BOT  CSA  AST  FTW  PMD  KH  ME  PN  PKE  MP  AUT  JC | | UTILITY  TOY  PASTORAL  UTILITY  GUNDOG  TERRIER  TERRIER  PASTORAL  UTILITY  TOY  TOY  TOY  TOY  TERRIER  TOY | | | | Unknown  Unknown  Unknown  -  Auto. rec. (suspected)  -  Breed at increased risk  Unknown  Unknown  Unknown  Unknown  Unknown  Unknown  Breed at increased risk  Breed at increased risk | 8 weeks  -  -  -  -  -  -  -  -  -  -  -  -  - | -  -  -  -  -  -  -  -  -  -  -  -  -  -  - |  | 64, 163, 446, 447 |
| Musculoskeletal | | | | | | Osteochondrosis dissecans (shoulder lameness) | | | | | | ES  LM  BMD  BDF  MF  GWP  PMD  HK  IW  NFL  OES  CBR  SB | | GUNDOG  GUNDOG  WORKING  WORKING  WORKING  GUNDOG  PASTORAL  PASTORAL  HOUND  WORKING  PASTORAL  GUNDOG  WORKING | | | | Polygenic  Breed at increased risk  Polygenic  Breed at increased risk  Breed at increased risk  Breed at increased risk  Unknown  Breed at increased risk  Breed at increased risk  Breed at increased risk  Breed at increased risk  Breed at increased risk  Breed at increased risk | 4-7 months  4-7 months  -  4-7 months  4-7 months  4-7 months  4-7 months  4-7 months  4-7 months  4-7 months  4-7 months  4-7 months  4-7 months | -  -  -  -  -  -  -  -  -  -  -  -  - |  | 64, 175, 281, 508 |
| Musculoskeletal | | | | | | Malignant hyperthermia | | | | | | GH | | HOUND | | | | Autosomal dominant | Birth | Yes |  | 232 |
| Musculoskeletal | | | | | | X-linked myopathy | | | | | | BGVG  JS  OES  WCP | | HOUND  UTILITY  PASTORAL  PASTORAL | | | | X-linked (assumed)  X-linked recessive  X-linked (assumed)  Unknown | 2 months  -  6 months  - | No | Exercise intolerance | 87, 260, 328, 342 |
| Musculoskeletal | | | | | | Congenital (X-linked) myopathy  (central core myopathy) | | | | | | IT  SD | | TERRIER  PASTORAL | | | | X-linked recessive  Unknown | -  - | No | Cardiomyopathy | 252, 253 |
| Musculoskeletal | | | | | | Chondrodysplasia  (osteochondrodysplasia) | | | | | | BGVP  WCC  WCP  GIT  PMD  HE  NFL  NEH  PKE  DH | | HOUND  PASTORAL  PASTORAL  TERRIER  PASTORAL  TOY  WORKING  HOUND  TOY  HOUND | | | | Dominant  (in basset hound)  Autosomal recessive  Autosomal recessive  Autosomal recessive  Autosomal recessive  Autosomal recessive  Unknown  Autosomal recessive | -  -  -  -  -  -  -  -  -  4-5 weeks | No | Deafness, patellar luxation, abnormal hips, glucosuria | 88, 219, 245, 309, 394 |
| Musculoskeletal | | | | | | Osteochondrosis (sacral) | | | | | | MF | | WORKING | | | | Unknown | - | No |  | 298 |
| Musculoskeletal | | | | | | Osteosarcoma  (Osteogenic sarcoma) | | | | | | GH  IW  LBR  NFL  MF  SB  DH | | HOUND  HOUND  WORKING  WORKING  WORKING  WORKING  HOUND | | | | Breed at increased risk in case series (familial)  Auto. dom (suspected) | Adult  (middle age) | No | Lung metastasis | 237, 287, 299, 372, 395 |
| Musculoskeletal | | | | | | Cystic bone lesions | | | | | | OES | | PASTORAL | | | | Familial, unknown | - | - |  | 325 |
| Musculoskeletal | | | | | | Radial head subluxation | | | | | | BDF | | WORKING | | | | Unknown | Birth | No | Skeletal deformities | 138 |
| Musculoskeletal | | | | | | English Pointer Enchondrodystrophy | | | | | | PR | | GUNDOG | | | | Autosomal recessive | 6 weeks | - |  | 348 |
| Musculoskeletal | | | | | | Hemivertebrae | | | | | | FB  PKE  BOT | | UTILITY  TOY  UTILITY | | | | Familial  Familial  Unknown | -  -  - | -  -  - | Hind limb weakness | 193, 469 |
| Musculoskeletal | | | | | | Intervertebral disc herniation | | | | | | FB  PKE | | UTILITY  TOY | | | | Unknown  Unknown | -  <2 years | No  - |  | 195, 338 |
| Musculoskeletal | | | | | | Perineal hernia | | | | | | BOT | | UTILITY | | | | Unknown | 9 years | - |  | 466 |
| Musculoskeletal | | | | | | Sacrocaudal dysgenesis | | | | | | BOT | | UTILITY | | | | Congenital | - | - |  | 470 |
| Musculoskeletal | | | | | | Hindlimb lameness | | | | | | GH | | HOUND | | | | Autosomal dominant (presumed) | 5 months | - |  | 230 |
| Musculoskeletal | | | | | | Hypertonic myopathy (exercise induced hypertonia) | | | | | | KCS | | TOY | | | | Unknown | - | - |  | 269 |
| Musculoskeletal | | | | | | Pyruvate dehydrogenase phosphatase 1 deficiency (Exercise intolerance, Exercise induced collapse) | | | | | | WCP  CBR  CCR  CBS  SS | | PASTORAL  GUNDOG  GUNDOG  GUNDOG  GUNDOG | | | | Autosomal recessive | -  1 year  -  1 year  1 year | No  Yes  -  Yes  Yes | Cardiomyopathy, severe exercise intolerance | 343, 418, 422 |
| Musculosketetal | | | | | | Hereditary necrotizing myopathy | | | | | | KHE | | GUNDOG | | | | Autosomal recessive | 3-12 months | - | Malacia of spinal cord with Wallerian degeneration | 272 |
| Musculoskeletal | | | | | | Periodontal disease | | | | | | IGH | | HOUND | | | | Unknown | 1-3 years | No |  | 244 |
| Musculoskeletal | | | | | | Enamel hypoplasia | | | | | | IGH | | HOUND | | | | Unknown | Juvenile | No | Periodontal disease | 245 |
| Musculoskeletal | | | | | | Oligodontia | | | | | | KBT | | TERRIER | | | | Familial | Juvenile | No |  | 268 |
| Musculoskeletal | | | | | | Hereditary spinal muscular atrophy | | | | | | BY  PR | | GUNDOG  GUNDOG | | | | Autosomal dominant  Unknown | < 1 year  5 months | -  - |  | 142, 429 |
| Musculoskeletal | | | | | | Syringomyelia | | | | | | GB | | TOY | | | | Polygenic | - | No |  | 146 |
| Musculoskeletal | | | | | | Mucopolysaccharidosis (Maroteaux-Lamy syndrome) | | | | | | MP | | TOY | | | | Autosomal recessive | - | Yes | Facial dysmorphia, paralysis | 345 |
| Musculoskeletal | | | | | | Glycogen storage disease (Pompe disease, all types) | | | | | | FL  ME  CCR  CSA  SL | | PASTORAL  TOY  GUNDOG  GUNDOG  PASTORAL | | | | Mutation  Autosomal recessive  Autosomal recessive  Autosomal recessive  Mutation | -  <2 months  2 months  -  - | -  -  Yes  Yes  No | Megaoesophagus, exhaustion | 181, 290, 369, 410 |
| Nervous System | | | | | | Lacrimal punctal atresia | | | | | | BT | | TERRIER | | | | Unknown | - | - |  | 452 |
| Nervous System | | | | | | Aniridia | | | | | | CSD | | PASTORAL | | | | Unknown | - | No | Keratitis, cataract, glaucoma | 426 |
| Nervous System | | | | | | Vitreal degeneration  (Vitreal syneresis) | | | | | | GH  BOT  IGH | | HOUND  UTILITY  TOY | | | | Breed at increased risk  Breed at increased risk  Familial, unknown | >7 years  3 years  2 years | -  -  - | Cataract, glaucoma | 229, 423 |
| Nervous System | | | | | | Hereditary myelopathy  (Afghan myelopathy) | | | | | | AH | | HOUND | | | | Autosomal recessive | 3-13 months | - | Demyelination and myelomalacia | 13-16 |
| Nervous System | | | | | | Hypomyelinating neuropathy  (Dysmyelination of the central nervous syetem) | | | | | | BMD  CWCW  SD | | WORKING  UTILITY  PASTORAL | | | | Auto. rec. (suggested)  Familial  X-linked (suspected) | 9 weeks  Birth  2-8 weeks | -  -  - |  | 110, 161, 385 |
| Nervous System | | | | | | Hypertrophic neuropathy | | | | | | TM | | WORKING | | | | Autosomal recessive | 8-10 weeks | - |  | 296 |
| Nervous System | | | | | | Degenerative myelopathy | | | | | | BMD  PMD  IH  WCP  CBR | | WORKING  PASTORAL  HOUND  PASTORAL  GUNDOG | | | | Autosomal recessive  Unknown  Autosomal recessive  Autosomal recessive  Autosomal recessive | 8 years  Adult  Birth  11 years  5-13 years | Yes  No  -  Yes  Yes | Ataxia, hypermetria, seizures | 27, 118, 226, 341, 367, 476 |
| Nervous System | | | | | | Neuroaxonal dystrophy (NAD) | | | | | | GS  PN | | WORKING  TOY | | | | Autosomal recessive  Familial, unknown | Birth  3 months | No  - | Cerebellar hypoplasia, respiratory failure, tremor | 210, 332 |
| Nervous System | | | | | | Cerebellar hypoplasia | | | | | | FTW | | TERRIER | | | | Congenital, rare | 2-3 weeks | - |  | 498 |
| Nervous System | | | | | | Acral mutilation syndrome | | | | | | PR | | GUNDOG | | | | Autosomal recessive | 3-8 months | - |  | 347 |
| Nervous System | | | | | | Hereditary sensory neuropathy | | | | | | PR | | GUNDOG | | | | Autosomal recessive | 3-6 months | - | Self mutilation (Acral mutilation) | 518 |
| Nervous System | | | | | | Lethal astrocytosis | | | | | | GST | | GUNDOG | | | | Autosomal recessive | 3-4 weeks | - |  | 216 |
| Nervous System | | | | | | Motor neuron abiotrophy | | | | | | SKI | | HOUND | | | | Unknown | 9 weeks | - |  | 377 |
| Nervous System | | | | | | Galactosialidosis  (Lysosomal storage disease) | | | | | | SKE | | UTILITY | | | | Unknown | 5 years | - |  | 388 |
| Nervous System | | | | | | Mucopolysaccharidosis IIIB (Sanfilippo syndrome IIIB) | | | | | | SKE | | UTILITY | | | | Autosomal recessive | 3 years | Yes | Cerebellar atrophy | 390 |
| Nervous System | | | | | | Cerebellar cortical abiotrophies | | | | | | AT  BMH  CWCW  LR  PDO | | TERRIER  HOUND  UTILITY  GUNDOG  HOUND | | | | Unknown  Unknown  Unknown  Unknown  Auto. rec. (suspected) | -  -  -  -  2-3 weeks | No | Cerebellar hypoplasia, ataxia, tremors | 27, 28, 89, 279, 521 |
| Nervous System | | | | | | Cerebellar ataxia | | | | | | BSM  CC  CDT  IS  KBT | | PASTORAL  TOY  TOY  GUNDOG  TERRIER | | | | Autosomal recessive | 8 weeks  3-4 months  2 weeks  5 months  3-4 months | No  -  -  - |  | 101, 151, 150, 258 |
| Nervous System | | | | | | Hereditary ataxia | | | | | | FTS | | TERRIER | | | | Autosomal recessive | 2-6 months | - | Myelopathy | 186, 191 |
| Nervous System | | | | | | Hound ataxia | | | | | | FH | | HOUND | | | | Unknown | 2-7 years | - |  | 495 |
| Nervous System | | | | | | Spinocerebellar degeneration | | | | | | BY  PRT | | GUNDOG  TERRIER | | | | Autosomal dominant  Autosomal recessive | 6-7 years  6-12 months | -  Yes | Ataxia, tremors, hind limb hypermetria | 144, 334 |
| Nervous System | | | | | | Cerebellar degeneration | | | | | | CDT  GST  OES | | TOY  GUNDOG  PASTORAL | | | | Unknown  Autosomal recessive  Autosomal recessive | 8 weeks  6-24 months  Adult-Elderly | -  -  - | Autoimmune deficiencies, hypermetria, ataxia | 165, 214, 326 |
| Nervous System | | | | | | Canine multiple system degeneration (CMSD) | | | | | | CC  KBT | | TOY  TERRIER | | | | Autosomal recessive  Autosomal recessive | -  3-6 months | -  - |  | 151 |
| Nervous System | | | | | | Lissenencephaly | | | | | | FTW | | TERRIER | | | | Unknown, rare | < 1 year | - |  | 498 |
| Nervous System | | | | | | Meningoencephalitis | | | | | | GH | | HOUND | | | | Unknown | 4-18 months | - | Ataxia, blindness | 235 |
| Nervous System | | | | | | Polioencephalomyelopathy | | | | | | ACD | | PASTORAL | | | | Maternal, mitochondrial | 5-6 months | - | Hypertrophy of astrocytes | 50-52 |
| Nervous System | | | | | | Degenerative encephalomyelopathy | | | | | | HK | | PASTORAL | | | | Familial | - | No | Ataxia | 248 |
| Nervous System | | | | | | Necrotizing encephalitis | | | | | | ME | | TOY | | | | Familial, unknown | >9 months | - | Ataxia, depression, generalized seizures | 292 |
| Nervous System | | | | | | Australian Stumpy Tailed Cattle Dog | | | | | | ACD | | PASTORAL | | | | Associated with coat colour and speckling | - | No | Deafness (unilateral or bilateral) | 59 |
| Nervous System | | | | | | Hepatocerebellar degeneration | | | | | | BMD | | WORKING | | | | Autosomal recessive | 1-2 months | - | Ataxia, head tremors | 109 |
| Nervous System | | | | | | Hydrocephalus  (Dandy Walker-like syndrome) | | | | | | BOT | | UTILITY | | | | Congenital | < 3 months | - |  | 468 |
| Nervous System | | | | | | White/Grey matter spongy degeneration | | | | | | AST  SKI | | TOY  HOUND | | | | Autosomal recessive  Unknown | -  - | -  - | Tremor, ataxia | 27 |
| Nervous System | | | | | | Malonic acidurea | | | | | | ME | | TOY | | | | Autosomal recessive (probable) | 3 years | - | Hypoglucaemia, acidosis | 291 |
| Nervous System | | | | | | Generalized tremors | | | | | | ME | | TOY | | | | Familial | < 5 years | - |  | 292 |
| Nervous System | | | | | | Intradural vasculitis | | | | | | WSS | | GUNDOG | | | | Familial | - | - | Ataxia and pyrexia, necrotizing arthritis | 425 |
| Nervous System | | | | | | Microphthalmia  (and/or Coloboma) | | | | | | AS  SB  SCWT  BJI  KCS  NT | | PASTORAL  WORKING  TERRIER  HOUND  TOY  TERRIER | | | | Autosomal recessive  Familial, unknown  Unknown  Unknown  Auto. rec. (suspected)  Unknown | -  -  -  -  -  - | -  -  -  -  -  - | Retinal dysplasia, cataract, persistent pupillary membranes | 65, 371, 405, 423 |
| Nervous System | | | | | | Deafness  (sensorial, sensorineural, congenital deafness with suspected inheritance,  Schiebe’s type deafness) | | | | | | FH  ACD  ES  PMD  GH  BI  AS  BOT  WCC  CC  CWCW  CDT  FB  HE  IH  IGH  KH  LLD  ME  NSDT  OES  PN  HP  SB  SD  ST  SS  TS  PR  HK  MP  SZR  SCWT  ASD | | HOUND  PASTORAL  GUNDOG  PASTORAL  HOUND  HOUND  PASTORAL  TERRIER  PASTORAL  TOY  UTILITY  TOY  UTILITY  TOY  HOUND  HOUND  PASTORAL  TOY  TOY  GUNDOG  PASTORAL  TOY  PASTORAL  WORKING  PASTORAL  TERRIER  GUNDOG  UTILITY  GUNDOG  PASTORAL  TOY  UTILITY  TERRIER  PASTORAL | | | | Incomplete dominant  Unknown  Familial  Unknown  Familial (coat colour)  Unknown  Unknown  Unknown  Unknown  Unknown  Unknown  Unknown  Unknown  Unknown  Unknown  Unknown  Unknown  Unknown  Unknown  Unknown  Incomplete dominant  Unknown  Unknown  Unknown  Familial, unknown  Unknown  Unknown  Unknown  Autosomal recessive  Unknown  Unknown  Unknown  Unknown  Unknown | -  4 weeks  6 weeks  4 weeks  -  -  -  -  -  -  -  -  -  -  -  -  -  -  < 4 months  -  -  -  -  -  -  -  -  -  -  -  -  -  -  - | Yes | Often syndromic deafness, microphthalmia and other eye defects, impairment of sperm function | 34-38, 54, 220, 322, 349, 350 |
| Nervous System | | | | | | Leukodystrophy | | | | | | ACD | | PASTORAL | | | | Familial, mitochondrial transmission | 2-9 weeks | No | Ataxia, paresis, paralysis, spasticity, cranial nerve dysfunction | 52, 58 |
| Nervous System | | | | | | Alexander Disease  (progressive paralysis and dementia) | | | | | | BMD | | WORKING | | | | Unknown | 9 weeks | - | Demyelination | 112 |
| Nervous System | | | | | | Epilepsy  (Grand mal seizures, idiopathic epilepsy) | | | | | | AS  BST  BMD  BY  CD  FS  GS  PMD  GSMD  IGH  WSS  IW  IS  KH  LR  POM  SB  SZR  FTW  PR | | PASTORAL  PASTORAL  WORKING  GUNDOG  UTILITY  HOUND  WORKING  PASTORAL  WORKING  TOY  GUNDOG  HOUND  GUNDOG  UTILITY  GUNDOG  TOY  WORKING  UTILITY  TERRIER  GUNDOG | | | | Breed at increased risk  Single locus  Polygenic recessive  Unknown  Unknown  Unknown  Unknown  Unknown  Unknown  Unknown  Unknown  Autosomal recessive  Unknown  Autosomal recessive  Familial  Unknown  Unknown  Unknown  Unknown  Breed at increased risk | 6 months  6 mo-6 years  1-3 years  -  -  -  -  -  -  -  -  3 years  -  >12 months  5-9 weeks  -  6 mo- 6 years  -  6 mo- 6 years  - | No  -  -  -  -  -  -  -  -  -  -  -  -  -  Yes  -  -  -  -  - | Ataxia, crawling, swaying, fearful behaviour, disorientation | 68, 102, 106, 145, 182, 227, 257, 259, 262, 280, 354, 438 |
| Ocular | | | | | | Cataract  (early and late onset) | | | | | | AH  AS  BC  BT  BST  RBT  BOT  CWCW  FTS  FTW  BP  HE  IH  IRWS  GS  LBR  NBH OES  CBR  CSA  WSS  LT  NWT  PR  SD  AST  ACD  BMD  BDF  GB  WCC  WCP  FS  FB  GP  GWP  IW  IGH  KH  KBT  KCS  ME  MT  NT  NEH  PN  PKE  MP  POM  CCR  SB  SKE  SZR  CBS  IWS  AWS  FSL  AUT  JC  CED  EBMD | | HOUND  PASTORAL  PASTORAL  TERRIER  PASTORAL  TERRIER  UTILITY  UTILITY  TERRIER  TERRIER  WORKING  TOY  HOUND  GUNDOG  WORKING  WORKING  PASTORAL  PASTORAL  GUNDOG  GUNDOG  GUNDOG  TERRIER  TERRIER  GUNDOG  PASTORAL  TOY  PASTORAL  WORKING  WORKING  TOY  PASTORAL  PASTORAL  HOUND  UTILITY  WORKING  GUNDOG  HOUND  TOY  UTILITY  TERRIER  TOY  TOY  TERRIER  TERRIER  HOUND  TOY  TOY  TOY  TOY  GUNDOG  WORKING  UTILITY  UTILITY  GUNDOG  GUNDOG  GUNDOG  GUNDOG  TERRIER  TOY  WORKING  WORKING | | | | Autosomal recessive  Mutation  Unknown  Autosomal recessive  Autosomal recessive  Unknown  Autosomal recessive  Familial  Unknown  Unknown  Autosomal recessive  Breed at increased risk  Autosomal recessive  Auto. dom (suspected)  Unknown  Familial  Autosomal dominant  Autosomal recessive  Autosomal dominant  Autosomal recessive  Autosomal recessive  Autosomal recessive  Autosomal recessive  Auto. dom (suspected)  Auto. rec. (suspected)  Unknown  Unknown  Unknown  Unknown  Unknown  Unknown  Unknown  Unknown  Unknown  Auto. rec. (suspected)  Unknown  Unknown  Unknown  Unknown  Unknown  Unknown  Breed at increased risk  Breed at increased risk  Unknown  Unknown  Unknown  Unknown  Breed at increased risk  Unknown  Unknown  Unknown  Unknown  Unknown  Unknown  Unknown  Unknown  Unknown  Unknown  Breed at increased risk  Unknown  Autosomal recessive | 4 months  -  2-5 years  2 months  2 years  -  8 wks, 5 yrs  -  -  3 years  -  -  7 months  -  6-7 years  -  6 weeks  -  6 mo-2 years  1-5 years  8-12 weeks  1-3 years  6mo-2 years  2-3 years  6 months  4-5 years  -  1 year  3 mo -1 year  -  -  1 year  -  6 mo -3 years  -  6-18 months  1-2 years  2-3 years  5 months  2 years  6 months  -  5 years  5 years  1-3 years  2-3 years  -  1-5 years  4 years  5-8 years  6-18 months  7 years  < 1 year  -  5 years  < 1 year  3 years  -  -  -  1-2 years | No  Yes  -  -  -  -  -  -  -  -  -  -  -  -  -  No  -  -  -  -  -  -  -  -  -  -  -  -  -  -  -  -  -  Yes  -  -  -  -  -  -  -  -  -  -  -  -  -  -  -  -  -  -  -  -  -  -  -  -  -  -  - | -  Microphthalmia (merle dogs)  Associated with PHTVL | 9, 10, 71, 96, 119, 126, 152, 187, 202, 250, 286, 312, 320, 364, 370, 409, 423, 432, 531 |
| Ocular | | | | | | Primary glaucoma  (POAG, PCAG) | | | | | | BDF  CWCW  DDT  FTW  FTS  CSA  NEH  SD  SB  ACD  JSI  BOT  BY  WCC  GS  GH  IGH  LT  ME  MP  ST  SKY  WSS  WT  MT  PKE | | WORKING  UTILITY  TERRIER  TERRIER  TERRIER  GUNDOG  HOUND  PASTORAL  WORKING  PASTORAL  UTILITY  TERRIER  GUNDOG  PASTORAL  WORKING  HOUND  HOUND  TERRIER  TOY  TOY  TERRIER  TERRIER  GUNDOG  TERRIER  TERRIER  TOY | | | | Unknown  Unknown  Unknown  Unknown  Unknown  Unknown  Unknown  Unknown  Unknown  Unknown  Unknown  Breed at increased risk  Breed at increased risk  -  -  Breed at increased risk  Breed at increased risk  Breed at increased risk  Breed at increased risk  Breed at increased risk  Breed at increased risk  Breed at increased risk  Autosomal dominant  Unknown  Breed at increased risk  Unknown | -  3-6 years  > 6 years  -  -  6 years  >5 years  6 years  -  -  -  -  -  -  -  -  2 years  -  6-16 years  -  -  -  10 weeks  -  2-4 years  - | -  -  -  -  -  -  -  -  -  -  -  -  -  -  -  -  -  -  -  -  -  -  -  -  -  - | Goniodysgenesis, increased intraocular pressure, lens luxation | 137, 154, 170, 187, 265, 315, 399, 403, 423, 424 |
| Ocular | | | | | | Goniodysgenesis | | | | | | JSI  CSA  WSS  SWD  BDF  CWCW  SD | | UTILITY  GUNDOG  GUNDOG  GUNDOG  WORKING  UTILITY  PASTORAL | | | | Unknown | Varies with breed | No | Primary closed angle glaucoma (PCAG) | 250, 424, 452, 533, 534, 535, 536 |
| Ocular | | | | | | Corneal oedema | | | | | | AH | | HOUND | | | | Unknown | - | - | Anterior uveitis, corneal endothelial damage | 24, 25 |
| Ocular | | | | | | Corneal ulcers | | | | | | PKE | | TOY | | | | Unknown | - | - | Eye protrusion | 337 |
| Ocular | | | | | | Corneal dystrophy  (endothelial) | | | | | | AT  BOT  KCS  PR  CSA | | TERRIER  UTILITY  TOY  GUNDOG  GUNDOG | | | | Sex-linked recessive  Unknown  Unknown  Unknown  Unknown | 10 months  5-9 years  2-5 years  6 years  1-7 years | -  -  -  -  - | Loss of vision by 4 years, lipid dystrophy | 29, 128, 423 |
| Ocular | | | | | | Spontaneous chronic corneal epithelial defects (SCCED) | | | | | | BOT  KH | | UTILITY  UTILITY | | | | Breed at increased risk | 6-8 years  8-9 years | -  - | Refractory corneal ulceration, indolent ulcers | 423, 500 |
| Ocular | | | | | | Choroidal hypoplasia  (Collie Eye Anomaly, CEA) | | | | | | CS  LH  AS | | PASTORAL  PASTORAL  PASTORAL | | | | Autosomal recessive  Auto. rec. (suspected)  Autosomal recessive | Puppy-Adult | Yes | Intraocular haemorrhage, blindness, defects of choroid, retina and optic nerve, retinal detachment | 60-63, 250, 278 |
| Ocular | | | | | | Nictitans gland prolapse (including plasmona) | | | | | | FB  CSA  NMF  BH  BI  BOT  PKE  SB | | UTILITY  GUNDOG  WORKING  HOUND  HOUND  UTILITY  TOY  WORKING | | | | Unknown  Breed at increased risk  Unknown  Breed at increased risk  Breed at increased risk  Breed at increased risk  Breed at increased risk  Breed at increased risk | <2 years  1-2 years  1-2 years  -  1-2 years  1-2 years  1-2 years | -  No  -  -  -  -  -  - | Keratoconjunctivitis sicca | 200, 265, 297, 423 |
| Ocular | | | | | | Keratoconjunctivitis sicca | | | | | | BH  BOT  PKE  CSA | | HOUND  UTILITY  TOY  GUNDOG | | | | Unknown  Breed at increased risk  Breed at increased risk  Breed at increased risk | -  -  -  - | -  -  -  - |  | 458, 526 |
| Ocular | | | | | | Ceroid lipofuscinosis  (progressive vision loss) | | | | | | ACD  AS  ES  SKI  PLS | | PASTORAL  PASTORAL  GUNDOG  HOUND  PASTORAL | | | | Autosomal recessive  Autosomal recessive  Autosomal recessive  Unknown  Auto. rec. (suspected) | 12-18 months  -  2-3 weeks  1-2 years  - | No  -  - | Neurological deficits, blindness, gyrate atrophy, dementia | 40-43, 72, 73, 173, 376, 438, 527 |
| Ocular | | | | | | Canine multifocal retinopathy | | | | | | AS  CDT  MF  PMD | | PASTORAL  TOY  WORKING  PASTORAL | | | | Autosomal recessive | -  4 months  13 weeks  13 weeks | Yes |  | 74, 75, 223 |
| Ocular | | | | | | Lens luxation | | | | | | ACD  CC  FTS  FTW  LH  PRT  ST  WT  WCC  WCP  GH  MT  NT  NWT  NEH  MBT | | PASTORAL  TOY  TERRIER  TERRIER  PASTORAL  TERRIER  TERRIER  TERRIER  PASTORAL  PASTORAL  HOUND  TERRIER  TERRIER  TERRIER  HOUND  TERRIER | | | | Autosomal recessive  Unknown  Unknown  Unknown  Autosomal recessive  Unknown  Unknown  Unknown  Unknown  Unknown  Unknown  Breed at increased risk  Unknown  Unknown  Unknown  Unknown | Adult  > 3 years  4-7 years  4-7 years  > 3 years  >3 years  4-6 years  5-6 years  -  -  3-5 years  2-4 years  -  -  2-6 years  > 3 years | Yes  -  -  -  Yes  Yes  Yes  Yes  -  -  -  -  -  -  -  Yes | Often accompanied with glaucoma | 48, 49, 187, 188, 250, 277, 423, 530 |
| Ocular | | | | | | Multiple ocular defects | | | | | | BH  BI  AS  IT  OES  PWD | | HOUND  HOUND  PASTORAL  TERRIER  PASTORAL  WORKING | | | | Unknown  Unknown  Auto. rec. (suggested)  Auto. rec. (suggested)  Unknown  Unknown | -  -  -  -  -  - | -  -  -  -  -  - | Cataract, mutltifocal retinal dysplasia, colobomas, microphthalmia | 65, 423, 460 |
| Ocular | | | | | | Uveal cysts | | | | | | BOT  CBR | | UTILITY  GUNDOG | | | | Breed at increased risk | 6 years  4-6 years | -  - |  | 423, 471 |
| Ocular | | | | | | Persistent hyperplastic primary vitreous (PHPV) | | | | | | BDF | | WORKING | | | | Breed at increased risk | - | - | Persistent hyperplastic tunic vasculosa lentis (PHTVL), retinal dysplasia, optic nerve hypoplasia, cataract, congenital blindness | 136 |
| Ocular | | | | | | Persistent hyperplastic tunic vasculosa lentis (PHTVL) | | | | | | GP | | WORKING | | | | Autosomal recessive | - | No |  | 201 |
| Ocular | | | | | | Persistent hyaloid artery | | | | | | SS | | GUNDOG | | | | Unknown | - | No |  | 423 |
| Ocular | | | | | | Persistent hyaloid remnants | | | | | | AS | | PASTORAL | | | | Unknown | - | No | Unilateral or bilateral obstruction of lens | 62, 67 |
| Ocular | | | | | | Persistent pupillary membranes | | | | | | BJI  WCC  WCP  CWCW  MF  LT  LH | | HOUND  PASTORAL  PASTORAL  UTILITY  WORKING  TERRIER  PASTORAL | | | | Unknown  Familial, unknown  Familial, unknown  Breed at increased risk  Auto. rec. (suspected)  Auto. rec. (suspected)  Breed at increased risk | -  -  -  -  -  -  - | -  -  -  -  -  -  - | Multifocal retinal dysplasia | 79-81, 250, 423 |
| Ocular | | | | | | Borzoi Chorioretinopathy | | | | | | BI | | HOUND | | | | Unique defect in breed, unknown | 7 mo - 7 years | No |  | 125 |
| Ocular | | | | | | Retinal pigment epithelial dystrophy (RPED)  (also hereditary retinal dystrophy, night blindness) | | | | | | BD  CS  WCC  PLS | | PASTORAL  PASTORAL  PASTORAL  PASTORAL | | | | Autosomal recessive  Unknown  Unknown  Unknown | <12 months  4-5 years  -  - | Yes  No  No  - |  | 250, 423, 528 |
| Ocular | | | | | | Retinal dysplasia  (multifocal & total) | | | | | | BT  BMD  ST  HP  CSA  SS  BY  WCP  GS  IW  NEH  CBR  SD  FSL | | TERRIER  WORKING  TERRIER  PASTORAL  GUNDOG  GUNDOG  GUNDOG  PASTORAL  WORKING  HOUND  HOUND  GUNDOG  PASTORAL  GUNDOG | | | | Autosomal recessive  Familial  Autosomal recessive  Auto. rec. (suspected)  Auto. rec. (suspected)  Unknown  Auto. rec. (suspected)  Auto. rec. (suspected)  Auto. rec. (suspected)  Auto. rec. (suspected)  Unknown  Auto. rec. (suspected)  Auto. rec. (suspected)  Auto. rec. (suspected) | Birth  3 months  -  -  -  -  -  -  -  -  -  -  -  - | -  No  -  -  -  -  -  -  -  -  -  -  -  - | Retinal detachment | 99, 115, 250, 396, 423 |
| Ocular | | | | | | Progressive retinal atrophy (PRA) (all types) Progressive rod and cone degeneration, rod-cone dystrophy | | | | | | ACD AS  BD  WCC  MF  FL  GIT  GST  GH  HK  IW  NEH  CBR  NSDT  CSA  TS  CC  PWD  SWD  SL  IRWS  SI  SD  PN  PKE  WSS  AST  FTW  FTS  BT  BMD  ME  MT  OES  MP  PR  CCR  FSL  CED  EBMD | | PASTORAL  PASTORAL  PASTORAL  PASTORAL  WORKING  PASTORAL  TERRIER  GUNDOG  HOUND  PASTORAL  HOUND  HOUND  GUNDOG  GUNDOG  GUNDOG  UTILITY  TOY  WORKING  GUNDOG  PASTORAL  GUNDOG  HOUND  PASTORAL  TOY  TOY  GUNDOG  TOY  TERRIER  TERRIER  TERRIER  WORKING  TOY  TERRIER  PASTORAL  TOY  GUNDOG  GUNDOG  GUNDOG  WORKING  WORKING | | | | Auto rec. or co-dom  Auto. rec. (suspected)  Auto. rec. (suspected)  Auto. rec. (suspected)  Autosomal dominant  Auto. rec. (suspected)  Autosomal recessive  Autosomal recessive  Autosomal recessive  Auto. rec. (suspected)  Autosomal recessive  Autosomal recessive  Auto. rec. (suspected)  Auto. rec. (suspected)  Autosomal recessive  Autosomal recessive  Auto. rec. (suspected)  Autosomal recessive  Auto. rec. (suspected)  Unknown  Autosomal recessive  Autosomal recessive  X-linked  Autosomal recessive  Autosomal recessive  Auto. rec. (assumed)  Autosomal recessive  Auto. rec. (suspected)  Auto. rec. (suspected)  Auto. rec. (suspected)  Familial  Auto. rec. (suspected)  Auto. rec. (suspected)  Auto. rec. (suspected)  Auto. rec. (suspected)  Auto. rec. (suspected)  Auto. rec. (suspected)  Auto. rec. (suspected)  Autosomal recessive  Auto. rec. (suspected) | 3-5 years  -  4 years  8 weeks  4 months  18 months  -  -  12 months  18 months  <6 months  6 weeks  8 mo, 7 years  -  2-3 years  3-7 years  -  3-5 years  18 months  -  6 months  -  2-5 years  > 7 years  8 years  5-7 years  5-11 years  2 years  -  1-2 years  <2 years  4-7 years  5-6 years  4 years  7 years  -  3-5 years  5 years  3-5 years  2-3 years | Yes  Yes  No  Yes  Yes  Yes  No  No  No  Yes  No  Yes  Yes  Yes  Yes  No  Yes  Yes  Yes  Yes  Yes  Yes  Yes  Yes  No  -  Yes  -  -  -  -  -  -  -  -  -  -  -  Yes  Yes | Extensive cataract formation | 53, 55, 115, 139, 147, 172, 180, 213, 218, 231, 250, 265, 333, 335, 423, 427, 435, 457, 511, 532 |
| Respiratory | | | | | | Brachycephalic airway syndrome | | | | | | AP  BOT  FB  PKE | | TOY  UTILITY  UTILITY  TOY | | | | Unknown | -  -  -  1-4 years | -  -  No  - | Associated with selective breeding for facial characters. Brachycephaly, stridor, gastrointestinal tract lesions | 4-8, 196, 336 |
| Respiratory | | | | | | Emphysema | | | | | | AH | | HOUND | | | | Familial, mode unknown | Elderly adult | - | Bronchial dysplasia, hypoplasia with hypoplastic cartilage | 12 |
| Respiratory | | | | | | Laryngeal paralysis  (muscular dystrophy of larynx) | | | | | | BDF  AH  PMD | | WORKING  HOUND  PASTORAL | | | | Autosomal dominant  -  Autosomal recessive | 2-6 months  -  < 2 years | -  -  No | Exercise intolerance | 4, 135, 362 |
| Respiratory | | | | | | Kartagener’s syndrome  (Situs invertus viscerum) | | | | | | CWCW | | UTILITY | | | | Autosomal recessive | < 3 months | - |  | 155, 483 |
| Respiratory | | | | | | Chylothorax  (pleural effusion) | | | | | | AH | | HOUND | | | | Unknown | Adult | - | Torsion of the accessory lung lobes | 17-22 |
| Respiratory | | | | | | Dyspnea  (Hypoplasic trachea) | | | | | | BOT | | UTILITY | | | | Unknown | Birth | - | Elongated soft palate, stenotic nares, cardiac defects, megaesophagus | 130 |
| Respiratory | | | | | | Tracheal collapse | | | | | | POM | | TOY | | | | Unknown | >6 years | - |  | 353 |
| Respiratory | | | | | | Dysphagia | | | | | | BDF | | WORKING | | | | Familial | - | - |  | 133 |
| Respiratory | | | | | | Rhinitis (Bronchopneumonia syndrome) | | | | | | IW | | HOUND | | | | Unknown | - | No |  | 256 |
| Respiratory | | | | | | Ciliary dyskinesia | | | | | | NFL  OES  PR | | WORKING  PASTORAL  GUNDOG | | | | Autosomal recessive  Autosomal recessive  Unknown | Adult  Adult  < 6 months | -  -  - | Pneumocid disease | 301, 321, 519 |
| Urogenital | | | | | | D-glyceric acidurea (Glycerate kinase deficiency) | | | | | | AH | | HOUND | | | | Autosomal recessive | - | - |  | 11 |
| Urogenital | | | | | | Pyruvate kinase deficiency | | | | | | BJI | | HOUND | | | | Autosomal recessive | - | Yes | Heamolytic anaemia | 82-84 |
| Urogenital | | | | | | Fanconi’s Syndrome  (renal tubular transport dysfunction) | | | | | | BJI | | HOUND | | | | Familial, presumed autosomal recessive | 1-5 years | Yes | Polyuream polydipsia, metabolic acidosis, cysteine uroliths, thyroid gland dysfunction | 85, 86 |
| Urogenital | | | | | | Cryptorchidism | | | | | | ME  OES  PKE  POM | | TOY  PASTORAL  TOY  TOY | | | | Auto. rec. (suspected)  Auto. rec. (suspected)  Breed at increased risk  Auto. rec. (suspected) | <Adult | -  -  -  - | Neoplasia | 289, 516 |
| Urogenital | | | | | | Urolithiasis  (Urate and oxalate) | | | | | | RBT  CDT  GS  LM  MF  PRT  AS  ACD  NFL  BJI  DH  MP  OES | | TERRIER  TOY  WORKING  GUNDOG  WORKING  TERRIER  PASTORAL  PASTORAL  WORKING  HOUND  HOUND  TOY  PASTORAL | | | | Autosomal recessive  Unknown  Unknown  Unknown  Autosomal recessive  Unknown  Breed at increased risk  Unknown  Unknown  Breed at increased risk  Unknown  Breed at increased risk  Breed at increased risk | -  3-4 weeks  -  -  2-5 years  -  2-5 years  2-5 years  -  2-5 years  2-5 years  2-5 years  - | Yes  -  -  No  -  Yes  -  -  -  -  -  -  - | Renal failure | 120, 167, 211, 295, 433, 513 |
| Urogenital | | | | | | Canine ectopic ureter | | | | | | FTS  FTW  NFL  SKY | | TERRIER  TERRIER  WORKING  TERRIER | | | | Breed at increased risk | < 1 year | -  -  -  - |  | 393, 497, 509 |
| Urogenital | | | | | | Vaginal hyperplasia  (Vaginal prolapse) | | | | | | MF  NMF | | WORKING  WORKING | | | | Breed at increased risk  Breed at increased risk | 1-3^rd^ oestrus  1^st^ -3^rd^ oestrus | -  - |  | 430, 489 |
| Urogenital | | | | | | Atresia ani  (Congenital anal stenosis) | | | | | | FS  BOT  ME  CWCW | | HOUND  UTILITY  TOY  UTILITY | | | | Breed at increased risk | -  -  -  - | -  -  -  - |  | 491 |
| Urogenital | | | | | | Glomerulocystic kidney disease | | | | | | BSM | | PASTORAL | | | | Unknown | Juvenile | - |  | 100 |
| Urogenital | | | | | | Familial nephropathy  (Familial renal disease; PLN, Protein losing nephropathy) | | | | | | BMD  NEH  SCWT | | WORKING  HOUND  TERRIER | | | | Auto. rec. (suggested)  Unknown  Familial, unknown | 3 years  < 5 months  > 6 years | -  -  - | Interstitial nephritis, renal disease | 108, 314, 406, 407 |
| Urogenital | | | | | | Hereditary nephritis  (Familial renal disease) | | | | | | SD | | PASTORAL | | | | X-linked dominant | 3-4 months | Yes | Hearing loss, renal failure | 382 |
| Urogenital | | | | | | Oxalate nephropathy | | | | | | TS | | UTILITY | | | | Unknown | - | - |  | 434 |
| Urogenital | | | | | | Hypospadias | | | | | | BOT | | UTILITY | | | | Congenital | - | - |  | 131 |
| Urogenital | | | | | | Renal telangiectasia | | | | | | WCP | | PASTORAL | | | | Unknown | 5-13 years | - | Hematouria | 340 |
| Urogenital | | | | | | Pyometra | | | | | | BMD  AT  IT  SB  LBR  KCS | | WORKING  TERRIER  TERRIER  WORKING  WORKING  TOY | | | | Breed at increased risk | 7 years  7 years  7 years  7 years  9 years  - | No |  | 117, 444, 457, 505 |
| Urogenital | | | | | | Prostatic carcinoma  (Canine prostate cancer) | | | | | | BDF  BMD | | WORKING  WORKING | | | | Breed at increased risk | Elderly | -  - |  | 473 |
| Urogenital | | | | | | Sry-negative sex reversal  (no Y chromosome) | | | | | | NEH  MP  CSA | | HOUND  TOY  GUNDOG | | | | Autosomal recessive (expression only in XX animals) | Birth | -  No |  | 316, 346, 413 |
| Urogenital | | | | | | Urethral sphincter mechanism incompetence | | | | | | OES | | PASTORAL | | | | Breed at increased risk | > 1 year | - |  | 512 |
| Urogenital | | | | | | Dystocia  (Obstructed labour) | | | | | | BOT  FB  POM  BMD  ME  WCP  GH  PKE  ST | | UTILITY  UTILITY  TOY  WORKING  TOY  PASTORAL  HOUND  TOY  TERRIER | | | | Breed at increased risk | -  -  -  -  -  -  -  -  - | No |  | 132, 198,233, 357, 430, 477 |
| Urogenital | | | | | | Perinatal mortality | | | | | | MH | | UTILITY | | | | Breed at increased risk | <1 month | - |  | 510 |
| Urogenital | | | | | | Cystinuria | | | | | | FB  IT  DH  WCC  AST  BJI  NFL  ACD | | UTILITY  TERRIER  HOUND  PASTORAL  TERRIER  HOUND  WORKING  PASTORAL | | | | Unknown  Breed at increased risk  Unknown  Breed at increased risk  Unknown  Unknown  Autosomal recessive  Autosomal dominant | 2-5 years  -  -  2-5 years  -  -  -  - | No  No  No  No  No  No  Yes  Yes | Renal failure | 197, 302, 433, 445 |
| Urogenital | | | | | | Renal amyloidosis | | | | | | FH | | HOUND | | | | Familial | - | - |  | 183 |
| Urogenital | | | | | | Renal dysplasia | | | | | | KHE  SCWT | | GUNDOG  TERRIER | | | | Familial  Unknown | <1 year  <1 year | -  Yes | Anemia, uraemia, renal failure | 273, 407 |
|  |  |  |  |  |  |  |  |  |  |  |  |  |  | |  |  |  |  |  |  |  |  |

**1.** Asher et al., (2009) **2.** Waldman (1995) **3.** Piek et al., (1996) **4.** O’Brien (1975) **5.** Hendricks (1992) **6.** Fasanella et al., (2010) **7.** Torrez & Hunt (2006) **8.** Bannasch et al., (2010) **9.** Roberts & Helper (1972) **10.** Barnett (1980) **11.** Sewell et al., (1997) **12.** Anderson et al., (1989) **13.** Cummings & deLahunta (1978) **14.** Averill & Bronson (1977) **15.** Jones & Richards (1977) **16.** Targett & McInnes (1998) **17.** Silverman & Kuttel (1982) **18.** Fossum et al., (1986) **19.** Williams & Duncan (1986) **20**. Gelzer et al., (1997) **21.** Neath et al., (2000) **22.** Johnston et al., (1984) **23.** Grondalen (1973) **24.** Curtis & Barnett (1981) **25.** Curtis & Barnett (1983) **26.** Miller & Dunstan (1993) **27.** Summers et al., (1995) **28.** Cordy & Snelbaker (1952) **29.** Dice (1976) **30.** Brooks (1999) **31**. Gu et al. (1999) **32.** Dodds et al., (1981) **33.** Villamil et al., (2011) **34.** Reetz et al., (1977) **35.** Strain (1996) **36.** Adams (1956) **37.** Hiraide & Paparella (1988) **38.** Strain et al., (2009) **39.** Temizsoylu & Avki (2003) **40.** Sisk et al., (1990) **41.** Vandevelde & Fatzer (1980) **42.** Wood et al., (1987) **43.** Studdert et al., (1991) **44.** Tisdall et al., (1994) **45.** Krotscheck et al., (2007) **46.** Van Steenbeek et al., (2011) **47.** Clements et al., (2007) **48.** Collier et al., (1989) **49.** Gould et al., (2011) **50.** Brenner et al., (1997) **51.** De Bosschere et al., (2002) **52.** Li et al., (2006) **53.** Laratta et al., (1988) **54.** Strain (2004) **55.** Zangerl et al., (2006) **56.** Finnigan et al., (2007) **57.** Gracis et al., (2000) **58.** Wood & Patterson (2001) **59.** Sommerland et al., (2010) **60.** Rubin et al., (1991) **61.** Lowe et al., (2003) **62.** Munyard et al., (2007) **63.** Parker et al., (2007) **64.** LaFond et al., (2002) **65.** Gelatt et al., (1981) **66.** Sponenberg & Bowling (1985) **67.** Senders et al., (1986) **68.** Weissl et al., (2011) **69.** Barbet et al., (2009) **70.** Gramer et al., (2010) **71.** Mellersh et al., (2009) **72.** O’Brien & Katz (2008) **73.** Katz et al., (2011) **74.** Guziewicz et al., (2007) **75.** Hoffmann et al., (2012) **76.** Breitschwerdt et al., (1982) **77.** Breitschwerdt et al., (1991) **78.**  Olivero et al., (2011) **79.** Barnett & Knight (1969) **80.** Mason (1976) **81.** James (1991) **82.** Searcy et al., (1971) **83.** Giger & Noble (1991) **84.** Whitney & Lothrop (1995) **85.** Bovee et al. (1978) **86.** Yearley et al., (2004) **87.** Klarenbeek et al., (2007) **88.** Parker et al., (2009) **89.** Flegel et al., (2007) **90.** Harper (1978) **91.** Schmutz et al., (1998) **92.** Ihrke et al., (1985**) 93.** Oberbauer et al., (2002) **94**. Ovrebo et al., (2001) **95.** Scott et al., (1995) **96.** Krohne (2001) **97.** Johnson et al., (1980) **98.** Yuzbasiyan et al., (1997) **99.** Dietz (1985) **100.** Ramos-Vara et al., (2004) **101.** Kleiter et al., (2011) **102.** Seppälä et al., (2012) **103.** Mahaffey et al., (1978) **104.** Lubbes et al., (2009) **105.** Beuing et al., (2005) **106.** Kathmann et al., (1999) **107.** Dodds (1989) **108.** Minkus et al., (1994) **109.** Carmichael ey al., (1996) **110.** Palmer et al., (1987) **111.** Abadie et al., (2009) **112.** Weissenbock et al., (1996) **113.** Willis (2000) **114.** Brewer et al., (1998) **115.** Chaudieu et al., (2004) **116.** Hayashi et al., (2004) **117.** Hagman et al., (2011) **118.** Wininger et al., (2011) **119.** Barnett (1985) **120.** Bende & Nemeth (2004) **121.** Evans & Adams (2010a) **122.** Janutta & Distl (2006) **123.** Hamil (1990) **124.** Conaway et al., (1985) **125.** Storey et al., (2005) **126.** Mellersh et al., (2007) **127.** Edmonds et al., (1972) **128.** Cooley & Dice (1990) **129.** Hayes et al. (1975) **130.** Coyne & Fingland (1992) **131.** Hayes & Wilson (1986) **132.** Eneroth et al., (1999) **133.** Peeters & Ubbink (1994) **134.** Comhaire & Snaps (2008) **135.** Burbidge (1995) **136.** Van Rensberg et al., (1992) **137.** Van der Linde-Sipman (1987) **138.** Temwichitir et al., (2010) **139.** Lightfoot et al., (1996) **140.** Richtsmeier et al., (1994) **141.** Cork et al., (1991)  **142.** Sack et al. (1984) **143.** Hubert et al., (1987) **144.** Higgins et al., (1998) **145.** Johnson & Patterson (2003) **146.** Knowler et al., (2014) **147.** Petersen-Jones et al., (1999)  **148.** Somberg et al., (1995) **149.** Tanaka et al., (2001) **150.** O’Brien et al., (2004) **151.** O’Brien et al., (2005) **152.** Collins et al., (1992) **153.** Kirberger & Stander (2007) **154.** Corcoran et al., (1994) **155.** Edwards et al., (1992) **156.** Farrow & Malik (1981) **157.** Ramos-Vara et al., (2000) **158.** Buchanan (1992) **159.** Gonsalves-Hubers (2005) **160.** Booth (1998) **161.** Vandevelde et al., (1978) **162.** Cerundolo & Lloyd (1998) **163.** Orthopedic Foundation (2003) **164.** Batchelor et al., (2007) **165.** Tipold et al., (2000) **166.** Coates et al., (2002) **167.** Vidgren et al., (2012) **168.** Grahn et al., (2006) **169.** Scholten-Sloof et al., (1992) **170.** Ahonen et al., (2013a)  **171.** Tobias & Rohrbach (2003) **172.** Kijas et al., (2003) **173.** Katz et al., (2005) **174.** Boari et al., (1994) **175.** Slater et al., (1991) **176.** Nachreiner et al., (2002) **177.** Marfe et al., (2012) **178.** Ginja et al., (2009) **179.** Lobo et al., (2010) **180.** Aguirre-Hernandez et al., (2007) **181.** Seppälä et al., (2013) **182.** Jeserevics et al., (2007) **183.** Mason et al., (1996) **184.** von Sandersleben et al., (1986) **185.** Jenkins et al., (1976) **186.** Bjorck et al., (1962) **187.** Gelatt & Mackay (2004) **188.** Curtis & Barnett (1980) **189.** Patterson (1989) **190.** Ordeix et al., (2009) **191.** Rohdin et al., (2010) **192.** Slappendel (1975) **193.** Done et al., (1975) **194.** Tanaka et al., (2003) **195.** Hansen (1952) **196.** Poncet et al., (2005) **197.** Hoppe & Denneberg (2001) **198.** Gaudet (1985) **199.** Oliveira et al., (2011) **200.** Mazzuccheli et al., (2012) **201.** Pfahler et al., (2014) **202.** Leppanen et al., (2001) **203.** Kim et al., (2005) **204.** Philipp et al., (2011) **205.** van Dongen et al., (2001) **206.** Genevois et al., (2008)  **207.** Greco et al., (1991)  **208.** Fyfe et al., (1991) **209.** Kidd et al., (2004) **210.** Fyfe et al., (2010) **211.** Karmi et al., (2010) **212.** Wilbe et al., (2010) **213.** Kropatsch et al., (2010) **214.** Tiemeyer et al., (1984) **215.** Glickman et al., (2000) **216.** Yaeger et al., (2000) **217.** Harlos (2010) **218.** Downs et al., (2013)  **219.** Bingel & Sande (1994) **220.** Coppens et al., (2000) **221.** Boudreaux et al., (1996) **222.** Golden et al., (1980) **223.** Grahn et al., (1998) **224.** Breur et al., (2001) **225**. Fogh & Fogh (1988) **226.** Wright & Brownlie (1985) **227.** Ekenstedt et al., (2011) **228.** Boudreaux & Martin (2011) **229.** Lynch (2007) **230.** Skelly et al. (1997) **231.** Slatter et al. (1980) **232.** Bagshaw et al., (1978) **233.** Bennett (1974) **234.** Cowan et al., (1997) **235.** Shiel et al., (2010) **236.** Gunby et al., (2004) **237.** Karlsson et al., (2013) **238.** Sams & Muir (1988) **239.** Gaughan & Bruyette (2001) **240.** Brenner et al., (2009) **241.** Drögemüller et al., (2010) **242.** Court et al., (1999) **243.** Pedersen et al., (2011) **244.** Roux et al., (2011) **245.** Starr et al., (2007) **246.** Rugbjerg et al., (2003) **247.** Ferm et al., (2009) **248.** Hazlett et al., (2005) **249.** Ruth (2012) **250.** Crispin & Warren (2008) **251.** Gu et al., (2004)  **252.** Presthus & Nordstoga (1993) **253.** Wentink et al., (1972) **254.** Binder et al., (2000) **255.** Martin et al., (2008)  **256.** Clercx et al., (2003) **257.** Casal et al., (2006) **258.** Wheeler & Rusbridge (1996) **259.** Hill (2006) **260.** Jones et al., (2004) **261.** Werner et al., (2005) **262.** Hall & Wallace (1996)  **263.** Goldstein et al., (2007) **264.** Buchanan & Patterson (2003) **265.** Gelatt (2007) **266.** Mausberg et al., (2008) **267.** Knowler et al., (1994) **268.** Kniazev et al., (2003) **269.** Wright et al., (1987) **270.** Swenson et al., (1996) **271.** Buchanan et al., (1997) **272.** Mandigers et al., (1993) **273.** Schulze et al., (1998) **274.** van Oost et al., (2004) **275.** Yamato et al., (1999) **276.** Lowe & King (2004) **277.** Sargan et al., (2007) **278.** Bedford (1998)  **279.** Jokinen et al., (2007a) **280.** Jokinen et al., (2007b) **281.** Coppieters et al., (2012) **282.** von Bomhard et al., (2006) **283.** Smallwood & Barsanti (1995) **284.** Hultin et al., (2011) **285.** Chetboul et al., (2003) **286.** Heinrich et al., (2006) **287.** Anfinsen et al., (2011) **288.** Krontveit et al., (2012) **289.** Hayes et al., (1985) **290.** Brix et al., (1995) **291.** O’Brien et al., (1999) **292.** Stalis et al., (1995) **293.** Parker et al., (2012) **294.** Vasseur et al., (1989) **295.** Osborne et al., (1999) **296.** Cummings et al., (1981) **297.** Morgan et al., (1993) **298.** Snaps et al., (1998) **299.** Kazmierski et al., (2001) **300.** Reist-Marti et al., (2012) **301.** Watson et al., (1999) **302.** Henthorn et al., (2000) **303.** Groondalen (1981) **304.** Kirberger & Fourie (1998) **305.** Kittleson & Kienle (1998) **306.** Fascetti et al., (2003) **307.** Lipsitz et al., (1999) **308.** Wilke et al., (2006) **309.** Young et al., (2006) **310.** Barnhart et al., (2004) **311.** Gelain et al., (2010) **312.** Bjerkas & Haaland (1995) **313.** Stannard & Pulley (1975) **314.** Wiersma et al., (2005) **315.** Oshima et al., (2004) **316.** Melniczek et al., (1999) **317.** Kyöstilä et al., (2013) **318.** Hughes et al., (2007) **319.** Green & Lantz (1978) **320.** Koch (1972) **321.** Merveille et al., (2014) **322.** Anniko et al., (1977) **323.** Williams & Maggio-Price (1984) **324.** Breitschwerdt et al., (1992) **325.** Watson & Dixon (1977) **326.** Steinberg et al., (2000) **327.** Hunt (2004) **328.** Wieczorek et al., (2006) **329.** Geyer et al., (2005) **330.** Boudreaux & Catalfamo (2001) **331.**  Wang et al., (1999) **332.** Nibe et al., (2007) **333.** Ahonen et al., (2013b) **334.** Forman et al., (2013) **335.** Priester (1974) **336.** Poncet et al., (2006) **337.** Petrick (1996) **338.** Goggin et al., (1970) **339.** Oswald & Orton (1993) **340.** Moore & Thorton (1983) **341.** Coates et al., (2007) **342.** Smith et al., (2011)  **343.** Minor et al., (2011) **344.** Campbell & Crow (2010) **345.** Neer et al., (1995) **346.** Nowacka et al., (2005) **347.** Cummings et al., (1981) **348.** Whitbread et al., (1983) **349.** Henthorn et al., (2004) **350.** Klein et al., (1988) **351.** Brooks et al., (2008) **352.** Tidholm (1997) **353.** Buback et al., (1996)  **354.** Varshney (2007) **355.** Takada et al., (2002) **356.** Mausberg et al., (2007) **357.** Bergstrom et al., (2006) **358.** Alroy et al., (2005) **359.** Wang et al., (2000) **360.** Oberbauer et al., (2006) **361.** Parker et al., (2010) **362.** Gabriel et al., (2006) **363.** Kemp et al., (2009) **364.** Gelatt et al., (1979) **365.** Johnson et al., (1988) **366.** Cerundolo et al., (2005) **367.** Awano et al., (2009) **368.** Coward (1989) **369.** Gregory et al., (2007) **370.** Lohmann & Klesen (1997) **371.** Martin & Leipold (1974) **372.** Bech-Nielsen et al., (1978) **373.** Szczulowska (1967) **374.** Villagomez & Alonso (1998) **375.** Kammermann et al., (1971) **376.** Appleby et al., (1982) **377.** Kent et al., (1999) **378.** Ogburn et al., (1981) **379.** Hargis et al., (1991) **380.** Kramer et al., (1988) **381.** Kimmel et al., (2002) **382.** Zheng et al., (1994) **383.** Acland & Aguirre (1991) **384.** McCaw & Aronson (1984) **385.** Cummings et al., (1986) **386.** Ling et al., (1979) **387.** Mulvihull & Priester (1973) **388.** Knowles et al., (1993) **389.** Delauche et al., (1998) **390.** Ellinwood et al., (2003)  **391.** Paltrinieri et al., (2007) **392.** Gillard et al., (2014) **393.** Holt & Moore (1995) **394.** Breur et al., (1989) **395.** Philips et al., (2007) **396.** Ashton et al., (1968) **397.** Fujise et al., (1997) **398.** Yamato et al., (2000) **399.** Kato et al., (2006a) **400.** Takeuchi et al., (2009) **401.** McGrotty et al., (2003) **402.** Lau (1977) **403.** Gelatt et al., (2008)  **404.** Peterson et al., (1996) **405.** Van der Woerdt et al. (1995) **406.** Littman et al., (2000) **407.** Eriksen & Grondalen (1984) **408.** Kittleson et al., (1997) **409.** Yakely (1971) **410.** Giger et al., (1992) **411.** Parry et al., (1988) **412.** Miller et al., (2000) **413.** Meyers-Wallen et al., (1995) **414.** Callan et al., (1995) **415.** Kwochka & Rademakers (1989)  **416.** Scott-Moncrieff et al., (2001) **417.** Hoppe & Svalastoga (1980)  **418.** Herrtage & Houlton (1979) **419.** Cerundolo et al., (2000) **420.** Freeman et al., (2013) **421.** Houlton & Herrtage (1980) **422.** Cameron et al., (2007) **423.** Rubin (1989) **424.** Cotrell & Barnett (1988) **425.** Caswell & Nykamp (2003) **426.** Hunter et al., (2007) **427.** André et al., (2008) **428.** Catchpole et al., (2013) **429.** Shelton (1999) **430.** Feldman & Nelson (1996) **431.** Goldschmidt & Shofer (1992) **432.** Gelatt & MacKay (2005) **433.** Case et al., (1992) **434.** Jansen & Arnesen (1990) **435.** Bjerkas & Narfstrom (1994) **436.** Ellison & Halling (2004) **437.** Miller et al., (1984) **438.** Bagley (2005) **439.** Giger (2003) **440.** Selby et al., (1981) **441.** Panciera (1994) **442.** Bryan et al., (2006) **443.** da Silva et al., (2012) **444.** Smith (2006) **445.** Brons et al., (2013)  **446.** Alam et al., (2007) **447.** Mortari et al., (2009) **448.** Baker-Gabb et al., (2003) **449.** Robins & Innes (2006) **450.** Scott et al., (2001a) **451.** Scott & Anderson (1991)  **452.**  Miller (2008) **453.** Lit et al., (2013) **454.** Chavkin et al., (1994) **455.** Zhou et al., (2010) **456.** Padgett et al., (1995) **457.** Egenvall et al., (2001) **458.** Kaswan & Salisbury (1990) **459.** Mills et al., (1985) **460.** Venter et al., (1996) **461.** Scott et al., (2001b) **462.** Cerundolo (1999) **463.** Capen & Martin (1975) **464.** Davis (1958) **465.** Bellenger et al., (1990) **466.** Bellenger (1980) **467.** Hayes & Fraumeni (1974) **468.** Noureddine et al., (2004) **469.** Grenn & Lindo (1969) **470.** Gutierrez-Quintana et al., (2014) **471.** Corcoran & Kock (1993) **472.** Paradis et al., (1989) **473.** Teske et al., (2002) **474.** Hargis et al., (1992) **475.** Wood et al., (2007) **476.** March et al., (2009) **477.** Evans & Adams (2010b) **478.** Morgan (1989) **479.** Engstrom (1966) **480.** Lothrop (1988) **481.** Witsberger et al., (2008) **482.** Schultheiss (2006) **483.** Edwards et al., (1989) **484.** Fox et al., (1999) **485.** Bussadori et al., (2001) **486.** Day (1994) **487.** Onions (1984) **488.** Bedford (1988) **489.** Post et al., (1991) **490.** Sueki et al., (1997) **491.** Vianna & Tobias (2005) **492.** Marmor (1982) **493.** Miller et al., (2004) **494.** Boudreaux et al., (1994) **495.** Palmer et al., (1984) **496.** Buchanan (1999) **497.** Hayes (1984) **498.** de Lahunta (1983) **499.** Brown et al., (1985) **500.** Murphy et al., (2001) **501.** Matic (1988) **502.** Patterson (1968) **503.** Smith & Knottenbelt (1988) **504.** Guilford et al., (1996) **505.** Niskanen & Thrusfield (1998) **506.** Borgarelli et al., (2006) **507.** Duval et al., (1999) **508.** Rudd et al., (1990) **509.** Ho et al., (2011) **510.** Kimura et al., (1993) **511.** Acland & Aguirre (1987) **512.** Holt & Thrusfield (1993) **513.** Aldrich et al., (1997) **514.** DeNovo (2003) **515.** D’Anna et al., (2007) **516.** Romagnoli (1991) **517.** Weaver (1983) **518.** Cummings et al., (1983) **519.** Morrison et al., (1987) **520.** Gurguis et al., (1990) **521.** van Tongern et al., (2000) **522.** Russell et al., (2006) **523.** Dukes-McEwan et al., (2003) **524.** Sevelius et al., (1994) **525.** Lawson (1973) **526.** Sanchez et al., (2007) **527.** Narfström et al., (2007) **528.** Watson et al., (1993)  **529.** Fall et al., (2007) **530.** Curtis et al., (1983) **531.** Spiess (1993) **532.** Heitmann et al., (2005) **533**. Bedford (1977) **534**. Rühli (1995) **535**. Kato et al. (2006b)  **536.** Ekesten & Torrang (1995)

**Supplementary Table 3**. UK Kennel Club 10-year registration statistics (2004-2013 inclusive)

| **Breed** | **Group** | **2004** | **2005** | **2006** | **2007** | **2008** | **2009** | **2010** | **2011** | **2012** | **2013** | **TOTAL** | **Rank** | **Vulnerable Breed Status** |
| --- | --- | --- | --- | --- | --- | --- | --- | --- | --- | --- | --- | --- | --- | --- |
| Retriever (Labrador) | GUNDOG | 45381 | 45779 | 45700 | 45079 | 45233 | 40943 | 44099 | 39964 | 36487 | 35026 | 423691 | 1 | N |
| Spaniel (Cocker) | GUNDOG | 16608 | 17468 | 20459 | 20883 | 22508 | 22211 | 23744 | 23258 | 23306 | 22943 | 213388 | 2 | N |
| Spaniel (English Springer) | GUNDOG | 14765 | 15180 | 15133 | 14702 | 14899 | 12700 | 13988 | 12883 | 12792 | 11316 | 138358 | 3 | N |
| German Shepherd | PASTORAL | 13578 | 13165 | 12857 | 12116 | 11903 | 10338 | 10364 | 9893 | 8502 | 7954 | 110670 | 4 | N |
| Staffordshire Bull Terrier | TERRIER | 12038 | 13070 | 12729 | 12167 | 10744 | 8746 | 8663 | 7113 | 6235 | 5767 | 97272 | 5 | N |
| Cavalier King Charles Spaniel | TOY | 10733 | 11165 | 11411 | 11422 | 11226 | 8884 | 8154 | 7446 | 5970 | 5145 | 91556 | 6 | N |
| Retriever (Golden) | GUNDOG | 10489 | 10165 | 9373 | 9557 | 9159 | 7804 | 7911 | 8081 | 7085 | 7117 | 86741 | 7 | N |
| Border Terrier | TERRIER | 7355 | 8338 | 8916 | 8814 | 9145 | 8214 | 8383 | 7188 | 6577 | 6390 | 79320 | 8 | N |
| Boxer | WORKING | 9689 | 9566 | 9066 | 8191 | 7353 | 5947 | 5699 | 5277 | 4622 | 4003 | 69413 | 9 | N |
| West Highland Terrier | TERRIER | 10110 | 9775 | 9300 | 8309 | 7330 | 5890 | 5361 | 4634 | 3975 | 3174 | 67858 | 10 | N |
| Miniature Schnauzer | UTILITY | 3347 | 4122 | 4396 | 5152 | 5333 | 5231 | 5651 | 5924 | 5797 | 5584 | 50537 | 11 | N |
| Pug | TOY | 1675 | 2116 | 2681 | 3547 | 4480 | 4769 | 5726 | 6221 | 7359 | 8071 | 46645 | 12 | N |
| Shih Tzu | UTILITY | 3263 | 3717 | 4436 | 5147 | 5495 | 5127 | 5247 | 5083 | 4565 | 4319 | 46399 | 13 | N |
| Lhasa Apso | UTILITY | 3485 | 3902 | 4154 | 4713 | 5117 | 4674 | 4865 | 4551 | 4449 | 3923 | 43833 | 14 | N |
| Bulldog | UTILITY | 2636 | 2997 | 3522 | 3979 | 4543 | 4217 | 4746 | 4659 | 4782 | 5769 | 41850 | 15 | N |
| Rottweiler | WORKING | 6726 | 6692 | 6575 | 4257 | 2631 | 2156 | 1959 | 1951 | 1554 | 1453 | 35954 | 16 | N |
| Yorkshire Terrier | TOY | 3877 | 3628 | 4042 | 4055 | 3951 | 3767 | 3441 | 3040 | 2505 | 2077 | 34383 | 17 | N |
| Whippet | HOUND | 2129 | 2468 | 2672 | 3043 | 3328 | 3246 | 3557 | 3295 | 3084 | 3226 | 30048 | 18 | N |
| Bull Terrier | TERRIER | 2996 | 3210 | 3361 | 3335 | 2922 | 2624 | 2467 | 2284 | 2132 | 1825 | 27156 | 19 | N |
| Dogue de Bordeaux | WORKING | 1593 | 1951 | 2361 | 2543 | 2452 | 2790 | 2841 | 2895 | 2431 | 2366 | 24223 | 20 | N |
| Border Collie | PASTORAL | 2230 | 2247 | 2219 | 2359 | 2375 | 2356 | 2604 | 2400 | 2144 | 2115 | 23049 | 21 | N |
| Bichon Frise | TOY | 2311 | 2246 | 2329 | 2694 | 2757 | 2430 | 2509 | 2217 | 1734 | 1614 | 22841 | 22 | N |
| Beagle | HOUND | 1451 | 1709 | 1817 | 2124 | 2405 | 2592 | 2877 | 2687 | 2728 | 2365 | 22755 | 23 | N |
| Doberman | WORKING | 3341 | 3316 | 3388 | 2427 | 1871 | 1600 | 1678 | 1457 | 1346 | 1212 | 21636 | 24 | N |
| Weimararner | GUNDOG | 2841 | 2848 | 2711 | 2724 | 2288 | 1951 | 1969 | 1581 | 1307 | 1261 | 21481 | 25 | N |
| French Bulldog | UTILITY | 350 | 324 | 526 | 692 | 1025 | 1521 | 2204 | 2771 | 4648 | 6990 | 21051 | 26 | N |
| Chihuahua (Long/smooth coat) | TOY | 788 | 884 | 1161 | 1435 | 1941 | 2104 | 2698 | 3060 | 3039 | 3111 | 20221 | 27 | N |
| Shar-Pei | UTILITY | 1449 | 1512 | 1987 | 2040 | 2261 | 2174 | 2304 | 2067 | 1706 | 1808 | 19308 | 28 | N |
| Siberian Husky | WORKING | 1527 | 1753 | 1993 | 2000 | 2094 | 2072 | 2209 | 1940 | 1684 | 1339 | 18611 | 29 | N |
| Dalmation | UTILITY | 2223 | 1930 | 1984 | 1657 | 1581 | 1356 | 1474 | 1488 | 1364 | 1163 | 16220 | 30 | N |
| Carin Terrier | TERRIER | 1761 | 1934 | 1834 | 1873 | 1946 | 1502 | 1453 | 1329 | 1035 | 1085 | 15752 | 31 | N |
| Great Dane | WORKING | 1728 | 1688 | 1839 | 1897 | 1419 | 1306 | 1429 | 1323 | 1281 | 1191 | 15101 | 32 | N |
| Poodle (Toy) | UTILITY | 1305 | 1517 | 1513 | 1671 | 1751 | 1403 | 1478 | 1476 | 1308 | 1304 | 14726 | 33 | N |
| Shetland Sheepdog | PASTORAL | 1638 | 1596 | 1577 | 1655 | 1634 | 1374 | 1333 | 1386 | 1085 | 1112 | 14390 | 34 | N |
| Retriever (Flat Coated) | GUNDOG | 1309 | 1707 | 1527 | 1718 | 1353 | 1233 | 1438 | 1387 | 1184 | 1299 | 14155 | 35 | N |
| German Shorthaired Pointer | GUNDOG | 1448 | 1489 | 1530 | 1497 | 1442 | 1281 | 1410 | 1424 | 1255 | 1367 | 14143 | 36 | N |
| Tibetian Terrier | UTILITY | 1303 | 1187 | 1378 | 1384 | 1600 | 1433 | 1429 | 1328 | 1283 | 1431 | 13756 | 37 | N |
| Rhodesian Ridgeback | HOUND | 1541 | 1493 | 1548 | 1618 | 1590 | 1101 | 1243 | 1200 | 1070 | 1066 | 13470 | 38 | N |
| Bullmastiff | WORKING | 1896 | 1623 | 1600 | 1594 | 1390 | 1149 | 1169 | 1044 | 948 | 672 | 13085 | 39 | N |
| Hungarian Vizsla | GUNDOG | 867 | 943 | 1030 | 1133 | 1306 | 1332 | 1498 | 1588 | 1607 | 1765 | 13069 | 40 | N |
| Akita | UTILITY | 1688 | 1462 | 1275 | 1375 | 1080 | 1046 | 1038 | 955 | 732 | 654 | 11305 | 41 | N |
| Basset Hound | HOUND | 1190 | 1375 | 1495 | 1258 | 1433 | 1031 | 1003 | 912 | 766 | 721 | 11184 | 42 | N |
| Irish Setter | GUNDOG | 1183 | 1278 | 1229 | 1029 | 1332 | 847 | 1126 | 869 | 924 | 859 | 10676 | 43 | N |
| Collie (Rough) | PASTORAL | 1241 | 1258 | 1023 | 1196 | 1171 | 870 | 1046 | 1046 | 943 | 842 | 10636 | 44 | N |
| Alaskan Malamute | WORKING | 586 | 718 | 889 | 1161 | 1245 | 1195 | 1232 | 1295 | 1053 | 851 | 10225 | 45 | N |
| Poodle (Standard) | UTILITY | 1055 | 1047 | 879 | 1057 | 1029 | 955 | 1037 | 900 | 963 | 832 | 9754 | 46 | N |
| Newfoundland | WORKING | 838 | 1080 | 1145 | 957 | 1046 | 898 | 1026 | 862 | 921 | 861 | 9634 | 47 | N |
| Poodle (Miniture) | UTILITY | 849 | 1009 | 978 | 1038 | 996 | 916 | 945 | 957 | 952 | 900 | 9540 | 48 | N |
| Scottish Terrier | TERRIER | 1168 | 1121 | 1137 | 1031 | 918 | 729 | 837 | 695 | 677 | 712 | 9025 | 49 | N |
| Dachund (Smooth/Long/Wire Hair) | HOUND | 676 | 696 | 738 | 817 | 940 | 848 | 914 | 889 | 837 | 815 | 8168 | 50 | N |
| Pomeranian | TOY | 671 | 703 | 830 | 703 | 830 | 761 | 810 | 717 | 676 | 731 | 7432 | 51 | N |
| Maltese | TOY | 373 | 404 | 441 | 557 | 624 | 831 | 911 | 997 | 1085 | 1143 | 7366 | 52 | N |
| Airedale Terrier | TERRIER | 988 | 727 | 861 | 869 | 864 | 601 | 599 | 612 | 600 | 555 | 7276 | 53 | N |
| Pointer | GUNDOG | 686 | 702 | 829 | 704 | 742 | 741 | 678 | 751 | 687 | 699 | 7219 | 54 | N |
| Papillon | TOY | 710 | 726 | 850 | 829 | 896 | 839 | 654 | 614 | 529 | 543 | 7190 | 55 | N |
| Boston Terrier | UTILITY | 242 | 265 | 298 | 430 | 566 | 576 | 823 | 1076 | 1255 | 1447 | 6978 | 56 | N |
| Bernese Mountain Dog | WORKING | 812 | 768 | 792 | 631 | 680 | 706 | 613 | 730 | 494 | 636 | 6862 | 57 | N |
| Parson Russell Terrier | TERRIER | 804 | 776 | 764 | 892 | 680 | 714 | 554 | 539 | 562 | 499 | 6784 | 58 | N |
| Fox Terrier (Wire) | TERRIER | 748 | 668 | 688 | 634 | 763 | 604 | 693 | 646 | 669 | 604 | 6717 | 59 | N |
| St Bernard | WORKING | 771 | 797 | 674 | 777 | 714 | 608 | 570 | 592 | 557 | 526 | 6586 | 60 | N |
| Bearded Collie | PASTORAL | 821 | 650 | 720 | 606 | 643 | 528 | 572 | 547 | 480 | 552 | 6119 | 61 | N |
| Norfolk Terrier | TERRIER | 543 | 663 | 631 | 686 | 635 | 603 | 534 | 577 | 546 | 568 | 5986 | 62 | N |
| Old English Sheepdog | PASTORAL | 603 | 663 | 536 | 692 | 529 | 413 | 507 | 401 | 429 | 461 | 5234 | 63 | N |
| Chinese Crested | TOY | 419 | 413 | 493 | 579 | 638 | 613 | 598 | 505 | 563 | 390 | 5211 | 64 | N |
| Bedlington Terrier | TERRIER | 536 | 531 | 471 | 495 | 505 | 491 | 569 | 558 | 506 | 482 | 5144 | 65 | N |
| Pekingese | TOY | 613 | 651 | 530 | 567 | 490 | 454 | 460 | 451 | 457 | 354 | 5027 | 66 | N |
| Welsh Corgi (Pembroke) | PASTORAL | 618 | 491 | 533 | 471 | 464 | 329 | 363 | 371 | 333 | 328 | 4301 | 67 | N |
| Italian Spinone | GUNDOG | 293 | 425 | 353 | 450 | 459 | 379 | 434 | 509 | 441 | 517 | 4260 | 68 | N |
| Spaniel (Welsh Springer) | GUNDOG | 420 | 442 | 352 | 376 | 510 | 381 | 338 | 396 | 348 | 353 | 3916 | 69 | N |
| Spaniel (American Cocker) | GUNDOG | 599 | 447 | 501 | 435 | 363 | 322 | 324 | 325 | 318 | 263 | 3897 | 70 | N |
| Chow Chow | UTILITY | 381 | 364 | 438 | 448 | 453 | 300 | 441 | 328 | 336 | 393 | 3882 | 71 | N |
| Irish Wolfhound | HOUND | 384 | 460 | 418 | 487 | 439 | 323 | 352 | 321 | 302 | 322 | 3808 | 72 | N |
| German Wirehaired Pointer | GUNDOG | 420 | 432 | 287 | 392 | 437 | 423 | 348 | 344 | 380 | 333 | 3796 | 73 | N |
| English Setter | GUNDOG | 547 | 427 | 450 | 416 | 399 | 295 | 349 | 234 | 314 | 326 | 3757 | 74 | N |
| Welsh Terrier | TERRIER | 305 | 326 | 386 | 377 | 360 | 337 | 432 | 415 | 352 | 447 | 3737 | 75 | N |
| Leonberger | WORKING | 348 | 353 | 438 | 383 | 446 | 306 | 399 | 358 | 298 | 391 | 3720 | 76 | N |
| Soft-Coated Wheaten Terrier | TERRIER | 287 | 321 | 403 | 324 | 352 | 284 | 405 | 433 | 455 | 372 | 3636 | 77 | N |
| Miniature Pinscher | TOY | 188 | 270 | 312 | 331 | 397 | 384 | 390 | 411 | 428 | 368 | 3479 | 78 | N |
| Hungarian Wire Haired Vizsla | GUNDOG | 183 | 160 | 253 | 252 | 262 | 284 | 393 | 473 | 368 | 620 | 3248 | 79 | N |
| Samoyed | PASTORAL | 387 | 408 | 338 | 339 | 354 | 280 | 333 | 316 | 245 | 224 | 3224 | 80 | N |
| Irish Terrier | TERRIER | 227 | 270 | 324 | 271 | 369 | 289 | 357 | 277 | 306 | 362 | 3052 | 81 | Y |
| Gordon Setter | GUNDOG | 356 | 309 | 345 | 312 | 362 | 192 | 306 | 245 | 252 | 273 | 2952 | 82 | N |
| Schnauzer | UTILITY | 347 | 335 | 367 | 218 | 331 | 279 | 282 | 231 | 272 | 286 | 2948 | 83 | N |
| Mastiff | WORKING | 434 | 476 | 483 | 354 | 299 | 252 | 157 | 173 | 140 | 139 | 2907 | 84 | N |
| Deerhound | HOUND | 331 | 264 | 252 | 327 | 309 | 324 | 256 | 237 | 260 | 236 | 2796 | 85 | Y |
| Lakeland Terrier | TERRIER | 225 | 330 | 277 | 269 | 303 | 283 | 254 | 247 | 208 | 221 | 2617 | 86 | Y |
| Giant Schnauzer | WORKING | 339 | 270 | 313 | 216 | 276 | 196 | 244 | 204 | 237 | 187 | 2482 | 87 | N |
| Japanese Chin | TOY | 201 | 239 | 233 | 242 | 279 | 246 | 302 | 261 | 256 | 182 | 2441 | 88 | N |
| Kerry Blue Terrier | TERRIER | 274 | 277 | 263 | 267 | 198 | 190 | 223 | 212 | 210 | 169 | 2283 | 89 | Y |
| Bull Terrier (Miniture) | TERRIER | 201 | 275 | 212 | 294 | 225 | 227 | 223 | 216 | 192 | 161 | 2226 | 90 | Y |
| Tibetan Spaniel | UTILITY | 254 | 238 | 264 | 249 | 202 | 227 | 232 | 217 | 193 | 140 | 2216 | 91 | N |
| Japanese Shiba Inu | UTILITY | 185 | 147 | 170 | 190 | 173 | 214 | 240 | 226 | 238 | 223 | 2006 | 92 | N |
| Neapolitan Mastiff | WORKING | 295 | 316 | 263 | 255 | 205 | 114 | 109 | 209 | 99 | 107 | 1972 | 93 | N |
| Spaniel (Clumber) | GUNDOG | 158 | 192 | 247 | 223 | 198 | 211 | 271 | 35 | 151 | 247 | 1933 | 94 | Y |
| King Charles Spaniel | TOY | 157 | 193 | 251 | 177 | 187 | 150 | 199 | 180 | 217 | 161 | 1872 | 95 | N |
| Retriever (Nova Scotia Duck Trolling) | GUNDOG | 99 | 99 | 157 | 162 | 187 | 162 | 195 | 304 | 210 | 192 | 1767 | 96 | N |
| Griffon Bruxellois | TOY | 172 | 185 | 174 | 170 | 224 | 173 | 201 | 160 | 148 | 155 | 1762 | 97 | N |
| Italian Greyhound | TOY | 114 | 114 | 97 | 137 | 157 | 193 | 215 | 260 | 208 | 231 | 1726 | 98 | N |
| Afghan Hound | HOUND | 235 | 213 | 194 | 198 | 147 | 140 | 122 | 124 | 162 | 138 | 1673 | 99 | N |
| Pyrenean Mountain Dog | PASTORAL | 222 | 220 | 178 | 159 | 211 | 143 | 78 | 180 | 105 | 82 | 1578 | 100 | N |
| Basset Griffon Vendeen (Petit) | HOUND | 209 | 220 | 154 | 136 | 102 | 178 | 122 | 142 | 154 | 148 | 1565 | 101 | N |
| Norwich Terrier | TERRIER | 124 | 131 | 147 | 128 | 152 | 160 | 172 | 158 | 170 | 194 | 1536 | 102 | Y |
| Fox Terrier (Smooth) | TERRIER | 188 | 212 | 173 | 137 | 178 | 133 | 155 | 137 | 94 | 122 | 1529 | 103 | Y |
| Havanese | TOY | 69 | 92 | 89 | 121 | 140 | 162 | 176 | 199 | 232 | 225 | 1505 | 104 | N |
| Australian Shepherd | PASTORAL | 164 | 90 | 176 | 113 | 155 | 181 | 135 | 142 | 124 | 202 | 1482 | 105 | N |
| Japanese Spitz | UTILITY | 118 | 112 | 154 | 181 | 190 | 165 | 126 | 129 | 151 | 125 | 1451 | 106 | N |
| Briard | PASTORAL | 144 | 158 | 161 | 112 | 201 | 148 | 112 | 159 | 126 | 126 | 1447 | 107 | N |
| Brittany | GUNDOG | 150 | 126 | 167 | 203 | 158 | 80 | 136 | 94 | 137 | 158 | 1409 | 108 | N |
| German Spitz (Klein) | UTILITY | 133 | 165 | 163 | 169 | 157 | 140 | 124 | 125 | 105 | 106 | 1387 | 109 | N |
| Spannish Water Dog | GUNDOG | 44 | 97 | 86 | 135 | 146 | 156 | 188 | 190 | 146 | 198 | 1386 | 110 | N |
| Manchester Terrier | TERRIER | 115 | 140 | 133 | 113 | 135 | 146 | 107 | 152 | 124 | 198 | 1363 | 111 | Y |
| Lancashire Heeler | PASTORAL | 147 | 166 | 173 | 146 | 158 | 133 | 134 | 98 | 104 | 103 | 1362 | 112 | Y |
| Coton de Tulear | TOY | 25 | 55 | 75 | 104 | 101 | 116 | 140 | 253 | 227 | 262 | 1358 | 113 | N |
| Saluki | HOUND | 104 | 86 | 104 | 73 | 131 | 189 | 238 | 140 | 150 | 95 | 1310 | 114 | N |
| Belgian Shepherd (Tervueren) | PASTORAL | 178 | 124 | 169 | 195 | 140 | 75 | 142 | 92 | 112 | 83 | 1310 | 115 | N |
| Large Munsterlander | GUNDOG | 165 | 133 | 122 | 154 | 119 | 140 | 102 | 119 | 96 | 108 | 1258 | 116 | N |
| Affenpinscher | TOY | 178 | 147 | 128 | 118 | 144 | 144 | 68 | 114 | 121 | 63 | 1225 | 117 | N |
| Spaniel (Irish Water) | GUNDOG | 121 | 106 | 105 | 162 | 101 | 131 | 117 | 101 | 148 | 101 | 1193 | 118 | Y |
| Borzoi | HOUND | 139 | 138 | 154 | 114 | 133 | 118 | 89 | 67 | 122 | 109 | 1183 | 119 | N |
| Retriever (Chesapeake Bay) | GUNDOG | 172 | 139 | 168 | 110 | 126 | 72 | 111 | 133 | 76 | 69 | 1176 | 120 | N |
| English Toy Terrier (Black & Tan) | TOY | 103 | 103 | 114 | 128 | 117 | 113 | 136 | 95 | 126 | 115 | 1150 | 121 | Y |
| Lowchen (Little Lion Dog) | TOY | 120 | 136 | 106 | 124 | 121 | 112 | 112 | 108 | 111 | 92 | 1142 | 122 | N |
| Dandie Dinmont Terrier | TERRIER | 81 | 149 | 78 | 124 | 119 | 108 | 151 | 98 | 120 | 105 | 1133 | 123 | Y |
| Bolognese | TOY | 63 | 67 | 72 | 113 | 136 | 128 | 133 | 113 | 131 | 143 | 1099 | 124 | N |
| Belgian Shepherd (Malinois) | PASTORAL | 66 | 74 | 86 | 92 | 129 | 92 | 138 | 116 | 111 | 151 | 1055 | 125 | N |
| Irish Red & White Setter | GUNDOG | 123 | 120 | 115 | 93 | 142 | 85 | 83 | 119 | 89 | 82 | 1051 | 126 | Y |
| Retriever (Curly Coated) | GUNDOG | 131 | 82 | 187 | 81 | 128 | 93 | 72 | 62 | 71 | 118 | 1025 | 127 | Y |
| Basset Griffon Vendeen (Grand) | HOUND | 137 | 100 | 84 | 127 | 81 | 53 | 107 | 116 | 102 | 117 | 1024 | 128 | N |
| Bouvier des Flandres | WORKING | 132 | 159 | 103 | 96 | 87 | 71 | 105 | 98 | 57 | 78 | 986 | 129 | N |
| Basset Fave de Bretagne | HOUND | 78 | 87 | 103 | 89 | 122 | 82 | 117 | 75 | 103 | 95 | 951 | 130 | N |
| Keeshond | UTILITY | 67 | 86 | 99 | 95 | 88 | 77 | 113 | 122 | 90 | 100 | 937 | 131 | N |
| Portuguese Water Dog | WORKING | 39 | 41 | 83 | 46 | 74 | 91 | 139 | 97 | 125 | 195 | 930 | 132 | N |
| Eurasier | UTILITY | 23 | 44 | 39 | 76 | 145 | 91 | 161 | 147 | 90 | 93 | 909 | 133 | N |
| Russian Black Terrier | WORKING | 136 | 129 | 129 | 93 | 54 | 35 | 57 | 66 | 75 | 52 | 826 | 134 | N |
| Welsh Corgi (Cardigan) | PASTORAL | 76 | 77 | 84 | 68 | 81 | 79 | 46 | 108 | 94 | 102 | 815 | 135 | Y |
| German Spitz (Mittel) | UTILITY | 72 | 85 | 80 | 104 | 68 | 94 | 87 | 71 | 82 | 57 | 800 | 136 | N |
| Bloodhound | HOUND | 126 | 104 | 70 | 100 | 114 | 60 | 55 | 59 | 50 | 51 | 789 | 137 | N |
| Norwegian Elkhound | HOUND | 118 | 116 | 84 | 98 | 55 | 98 | 33 | 42 | 62 | 71 | 777 | 138 | N |
| Belgian Shepherd (Groenendael) | PASTORAL | 126 | 66 | 40 | 92 | 89 | 90 | 95 | 60 | 71 | 33 | 762 | 139 | N |
| Australian Cattle Dog | PASTORAL | 93 | 90 | 69 | 80 | 84 | 92 | 69 | 65 | 65 | 33 | 740 | 140 | N |
| Collie (Smooth) | PASTORAL | 72 | 72 | 53 | 63 | 43 | 74 | 54 | 75 | 88 | 82 | 676 | 141 | Y |
| Portuguese Podengo | HOUND | 47 | 61 | 108 | 73 | 108 | 64 | 72 | 80 | 23 | 29 | 665 | 142 | N |
| Spaniel (Sussex) | GUNDOG | 79 | 77 | 74 | 61 | 56 | 60 | 68 | 52 | 74 | 55 | 656 | 143 | Y |
| Spaniel (Field) | GUNDOG | 86 | 86 | 64 | 67 | 75 | 51 | 55 | 46 | 47 | 29 | 606 | 144 | Y |
| Hungarian Puli | PASTORAL | 66 | 73 | 55 | 78 | 70 | 37 | 56 | 59 | 71 | 30 | 595 | 145 | N |
| Sealyham Terrier | TERRIER | 51 | 58 | 57 | 65 | 43 | 47 | 49 | 63 | 76 | 68 | 577 | 146 | Y |
| Polish Lowland Sheepdog | PASTORAL | 37 | 43 | 68 | 63 | 63 | 75 | 26 | 55 | 65 | 69 | 564 | 147 | N |
| Tibetan Mastiff | WORKING | 37 | 30 | 60 | 57 | 59 | 48 | 40 | 63 | 75 | 73 | 542 | 148 | N |
| Glen Of Imaal Terrier | TERRIER | 43 | 45 | 41 | 36 | 89 | 46 | 61 | 67 | 57 | 55 | 540 | 149 | Y |
| Schipperke | UTILITY | 77 | 70 | 88 | 23 | 63 | 51 | 49 | 31 | 39 | 37 | 528 | 150 | N |
| Finnish Lapphund | PASTORAL | 39 | 49 | 32 | 65 | 46 | 50 | 55 | 68 | 63 | 54 | 521 | 151 | N |
| Slovakian Rough Haired Pointer | GUNDOG | 11 | 30 | 54 | 53 | 55 | 68 | 56 | 61 | 43 | 69 | 500 | 152 | N |
| Bracco Italiano | GUNDOG | 23 | 31 | 51 | 31 | 57 | 46 | 79 | 43 | 72 | 62 | 495 | 153 | N |
| Australian Terrier | TERRIER | 60 | 60 | 62 | 38 | 55 | 57 | 36 | 46 | 30 | 33 | 477 | 154 | N |
| Basenji | HOUND | 53 | 55 | 37 | 42 | 59 | 37 | 49 | 28 | 46 | 46 | 452 | 155 | N |
| Skye Terrier | TERRIER | 50 | 30 | 84 | 37 | 27 | 75 | 37 | 44 | 42 | 17 | 443 | 156 | Y |
| Anatolian Shepherd | PASTORAL | 66 | 41 | 54 | 48 | 48 | 47 | 27 | 39 | 44 | 27 | 441 | 157 | N |
| Otterhound | HOUND | 11 | 50 | 51 | 41 | 38 | 57 | 57 | 38 | 37 | 42 | 422 | 158 | Y |
| Swedish Vallhund | PASTORAL | 40 | 39 | 46 | 40 | 40 | 50 | 29 | 43 | 33 | 58 | 418 | 159 | N |
| Japanese Akita Inu | UTILITY | 0 | 0 | 38 | 29 | 39 | 24 | 70 | 50 | 68 | 84 | 402 | 160 | N |
| Greyhound | HOUND | 31 | 49 | 44 | 48 | 46 | 33 | 65 | 14 | 30 | 40 | 400 | 161 | Y |
| Cesky Terrier | TERRIER | 31 | 57 | 44 | 60 | 29 | 28 | 34 | 24 | 26 | 26 | 359 | 162 | N |
| Bavarian Mountain Hound | HOUND | 3 | 20 | 10 | 16 | 32 | 54 | 73 | 39 | 10 | 80 | 337 | 163 | N |
| Lagotto Romagnola | GUNDOG | 47 | 20 | 25 | 16 | 31 | 32 | 23 | 35 | 45 | 46 | 320 | 164 | N |
| Estrela Mountain Dog | PASTORAL | 34 | 39 | 25 | 29 | 46 | 41 | 33 | 21 | 11 | 19 | 298 | 165 | N |
| Finnish Spitz | HOUND | 36 | 37 | 23 | 35 | 36 | 20 | 36 | 10 | 11 | 19 | 263 | 166 | N |
| Norwegian Buhund | PASTORAL | 36 | 15 | 20 | 28 | 22 | 22 | 29 | 14 | 32 | 45 | 263 | 167 | N |
| Korthals Griffon | GUNDOG | 13 | 20 | 16 | 22 | 14 | 43 | 26 | 37 | 16 | 55 | 262 | 168 | N |
| Pharoah Hound | HOUND | 35 | 20 | 21 | 9 | 27 | 36 | 38 | 29 | 14 | 27 | 256 | 169 | N |
| Hovawart | WORKING | 12 | 38 | 27 | 41 | 18 | 45 | 4 | 34 | 11 | 17 | 247 | 170 | N |
| Maremma Sheepdog | PASTORAL | 17 | 19 | 18 | 9 | 37 | 28 | 25 | 25 | 25 | 31 | 234 | 171 | N |
| Catalan Sheepdog | PASTORAL | 0 | 0 | 7 | 15 | 46 | 28 | 25 | 53 | 32 | 24 | 230 | 172 | N |
| German Longhaired Pointer | GUNDOG | 26 | 9 | 36 | 40 | 5 | 28 | 23 | 26 | 17 | 9 | 219 | 173 | N |
| Pyrenean Sheepdog (Long haired) | PASTORAL | 34 | 20 | 25 | 27 | 27 | 6 | 36 | 5 | 12 | 26 | 218 | 174 | N |
| Australian Silky Terrier | TOY | 19 | 31 | 18 | 23 | 41 | 11 | 23 | 11 | 19 | 11 | 207 | 175 | N |
| Hamiltonstovare | HOUND | 32 | 24 | 20 | 19 | 17 | 16 | 6 | 27 | 6 | 17 | 184 | 176 | N |
| Beauceron | WORKING | 8 | 24 | 16 | 20 | 8 | 25 | 1 | 31 | 19 | 23 | 175 | 177 | N |
| Kooikerhondje | GUNDOG | 17 | 0 | 11 | 11 | 5 | 11 | 14 | 10 | 33 | 33 | 145 | 178 | N |
| Greater Swiss Mountain Dog | WORKING | 0 | 0 | 0 | 0 | 5 | 24 | 23 | 26 | 22 | 41 | 141 | 179 | N |
| Canaan Dog | UTILITY | 22 | 13 | 22 | 13 | 8 | 6 | 12 | 24 | 10 | 8 | 138 | 180 | N |
| German Pinscher | WORKING | 19 | 10 | 14 | 15 | 16 | 1 | 12 | 11 | 13 | 15 | 126 | 181 | N |
| Ibizan Hound | HOUND | 6 | 14 | 7 | 7 | 17 | 25 | 8 | 7 | 19 | 4 | 114 | 182 | N |
| Cirneco Dell'Etna | HOUND | 3 | 7 | 1 | 10 | 23 | 10 | 8 | 22 | 3 | 19 | 106 | 183 | N |
| Korean Jindo | UTILITY | 0 | 0 | 19 | 13 | 6 | 13 | 21 | 0 | 14 | 11 | 97 | 184 | N |
| Greenland Dog | WORKING | 10 | 17 | 20 | 5 | 2 | 5 | 9 | 1 | 14 | 10 | 93 | 185 | N |
| Canadian Eskimo Dog | WORKING | 0 | 7 | 4 | 3 | 12 | 11 | 7 | 1 | 15 | 12 | 72 | 186 | N |
| Sloughi | HOUND | 19 | 6 | 0 | 8 | 0 | 4 | 8 | 1 | 14 | 11 | 71 | 187 | N |
| Komondor | PASTORAL | 3 | 0 | 13 | 1 | 16 | 8 | 6 | 9 | 9 | 2 | 67 | 188 | N |
| Mexican Hairless (Intermediate) | UTILITY | 0 | 0 | 0 | 5 | 3 | 6 | 15 | 6 | 20 | 2 | 57 | 189 | N |
| Foxhound | HOUND | 0 | 5 | 0 | 14 | 7 | 1 | 0 | 9 | 0 | 11 | 47 | 190 | N |
| Entlebucher Mountain Dog | WORKING | 5 | 0 | 1 | 1 | 1 | 6 | 6 | 7 | 11 | 6 | 44 | 191 | N |
| Grand Bleu de Gacogne | HOUND | 2 | 17 | 3 | 2 | 19 | 0 | 0 | 0 | 0 | 0 | 43 | 192 | N |
| Bergamasco | PASTORAL | 4 | 14 | 0 | 0 | 0 | 0 | 1 | 2 | 14 | 7 | 42 | 193 | N |
| Basset Bleu de Gascogne | HOUND | 8 | 4 | 7 | 1 | 0 | 5 | 0 | 4 | 0 | 11 | 40 | 194 | N |
| Belgian Shepherd (Laekenois) | PASTORAL | 1 | 0 | 0 | 0 | 13 | 2 | 17 | 0 | 0 | 1 | 34 | 195 | N |
| Mexican Hairless (Miniture) | UTILITY | 0 | 0 | 0 | 0 | 0 | 0 | 2 | 1 | 5 | 23 | 31 | 196 | N |
| Azawakh | HOUND | 11 | 7 | 1 | 0 | 1 | 1 | 6 | 0 | 3 | 0 | 30 | 197 | N |
| Portuguese Pointer | GUNDOG | 0 | 0 | 0 | 0 | 0 | 0 | 0 | 0 | 0 | 27 | 27 | 198 | N |
| Small Munsterlander | GUNDOG | 0 | 15 | 3 | 0 | 0 | 0 | 0 | 0 | 1 | 0 | 19 | 199 | N |
| Mexican Hairless (Standard) | UTILITY | 0 | 0 | 0 | 0 | 1 | 0 | 1 | 11 | 2 | 0 | 15 | 200 | N |
| Swedish Lapphund | PASTORAL | 1 | 0 | 1 | 7 | 0 | 3 | 0 | 0 | 1 | 1 | 14 | 201 | N |
| Turkish Kangal Dog | PASTORAL | 0 | 0 | 0 | 0 | 0 | 0 | 0 | 0 | 0 | 14 | 14 | 202 | N |
| Segugio Italiano | HOUND | 5 | 0 | 0 | 6 | 0 | 0 | 0 | 0 | 0 | 0 | 11 | 203 | N |
| Mexican Hairless | UTILITY | 2 | 1 | 4 | 0 | 0 | 0 | 0 | 0 | 0 | 0 | 7 | 204 | N |
| Pyrenean Mastiff | WORKING | 2 | 1 | 0 | 0 | 0 | 0 | 1 | 0 | 0 | 0 | 4 | 205 | N |
| Spaniel (American Water) | GUNDOG | 0 | 0 | 1 | 0 | 0 | 0 | 0 | 0 | 1 | 1 | 3 | 206 | N |
| Hungarian Kuvasz | PASTORAL | 2 | 0 | 1 | 0 | 0 | 0 | 0 | 0 | 0 | 0 | 3 | 207 | N |

**References**

Abadie, J., Hédan, B., Cadieu, E., De Brito, C., Devauchelle, P., Bourgain, C., Parker, H.G., Vaysse, A., Margaritte-Jeannin, P., Galibert, F., Ostrander, E.A., André, C. 2009. Epidemiology, pathology, and genetics of histiocytic sarcoma in the Bernese mountain dog breed. J Hered. 100 Suppl 1:S19-27.

Acland, G.M., Aguire, G.D. 1987. Retinal degeneration in the dog: IV: Early retinal degeneration in Norwegian Elkhounds. Experimental Eye Research, 44(4): 491-521.

Acland, G.M., Aguirre, G.D. 1991. Retinal dysplasia in the Samoyed dog is the heterozygous phenotype of the gene (drds) for short limbed dwarfism and ocular defects. Transactions of the American College Veterinary Ophthalmology 22:44.

Adams, E.W. 1956. Hereditary deafness in a family of Foxhounds. Journal of the American Veterinary Medical Association 128: 302-303.

Aguirre-Hernandez, J., Wickstrom, K., Sargan, D.R. 2007. The Finnish lapphund retinal atrophy locus maps to the centromeric region of CFA9. BMC Vet Res.3:14.

Ahonen S.J., Pietila E, Mellersh C.S., Tiira, K., Hansen, L., Johnson, G.S., Lohi, H. 2013a. Genome-wide association study identifies a novel canine glaucoma locus. PLoS ONE 8(8): e70903. doi:10.1371/journal.pone. 0070903.

Ahonen SJ, Arumilli M, Lohi H. 2013b. A CNGB1 frameshift mutation in Papillon and Phalène dogs with progressive retinal atrophy. PLoS One. 8:e72122. doi: 10.1371/journal.pone.0072122.

Alam, M.R., Lee, J.I., Kang, H.S., et al. 2007, Frequency and distribution of patellar luxation in dogs. 134 cases (2000 to 2005) Vet Comp Orthop Traumat. 20:59–64.

Aldrich, J., Ling, G. V., Ruby, A. L., Johnson, D. L., Franti, C. E. 1997. Silica‐Containing Urinary Calculi in Dogs (1981–1993). Journal of Veterinary Internal Medicine, 11(5), 288-295.

Alroy, J., Rush, J.E., Sarkar, S. 2005. Infantile dilated cardiomyopathy in Portuguese water dogs: Correlation of the autosomal recessive trait with low plasma taurine at infancy. Amino Acids. 28(1):51-6.

Anderson, W.I., King, J.M., Flint, T.J. 1989. Multifocal Bullous Emphysema with Concurrent Bronchial Hypoplasia in Two Aged Afghan Hounds. Journal of Comparative Pathology 100(4): 469.

André, C., G. Chaudieu, A. Thomas, O. Jongh, J-P. Jegou, S. Chahory, B. Clerc, P. Pilorge, and O. Brenac. 2008. Hereditary retinopathies in the dog: Genetic fundamentals and genetic tests. Pratique Médicale et Chirurgicale de l'Animal de Compagnie 43(2):75-84.

Anfinsen, K.P., Grotmol, T., Bruland, O.S., Jonasdottir, T.J. 2011. Breed-specific incidence rates of canine primary bone tumors--a population based survey of dogs in Norway. Can J Vet Res. 3:209-215.

Anniko, M., Fabiansson, E., Nilsson, O. 1977. Deafness in an old English sheepdog. A case report. Arch Otorhinolaryngol. 218(1-2):1-7.

Appleby, E.C., Longstaffe, J.A., Bell, F.R. 1982. Ceroid-lipofuscinosis in two Saluki dogs. Journal of Comparative Pathology 92(3):375-80.

Asher, L., Diesel, G., Summers, J.F., McGreevy, P.D., Collins, L.M. 2009. Inherited defects in pedigree dogs. Part 1: Disorders related to breed standards. The Veterinary Journal 182, 402-411.

Ashton, N., Barnett, K.C., Sachs, D.D. 1968. Retinal dysplasia in the Sealyham terrier. Journal of Pathology and Bacteriology 96(2):269-72.

Averill, D.R., Jr., Bronson, R.T. 1977. Inherited necrotizing Myelopathy of Afghan hounds. J Neuropathol Exp Neurol. 36:734-47

Awano, T., Johnson, G.S., Wade, C.M., Katz, M.L., Johnson, G.C., Taylor, J.F., Perloski, M., Biagi, T., Baranowska, I., Long, S., March, P.A., Olby, N.J., Shelton, G.D., Khan, S., O'Brien, D.P., Lindblad-Toh, K., Coates, J.R. 2009. Genome-wide association analysis reveals a SOD1 mutation in canine degenerative myelopathy that resembles amyotrophic lateral sclerosis. Proc Natl Acad Sci U S A. 2009 Feb 24;106(8):2794-9

Bagley, R.S., Kornegay, J.N., Wheeler, S.J., Plummer, S.B., Cauzinille, L. 1993. Generalized Tremors in Maltese - Clinical Findings in 7 Cases. Journal of the American Animal Hospital Association 29: 141-145.

Bagley, R.S. 2005. Clinical features of important and common diseases involving the intracranial nervous syetem of dogs and cats. In: Fundamentals of Veterinary Neurology, 1^st^ edition (ed Bagley, R.S.), Blackwell Publishing, Iowa, pp. 119-149.

Bagshaw, R.J., Cox, R.H., Knight, D.H., Detweiler, D.K. 1978. Malignant hyperthermia in a Greyhound. J Am Vet Med Assoc. 172(1):61-2.

Baker‐Gabb, M., Hunt, G. B., France, M. P. 2003. Soft tissue sarcomas and mast cell tumours in dogs; clinical behaviour and response to surgery. Australian veterinary journal, 81(12), 732-738.

Bannasch, D., Young, A., Myers, J., Truvé, K., Dickinson, P., Gregg, J., Davis, R., Bongcam-Rudloff, E., Webster, M.T., Lindblad-Toh, K., Pedersen, N. 2010. Localization of canine brachycephaly using an across breed mapping approach. PLoS One. Mar 10;5(3):e9632. PubMed PMID: 20224736

Barbet, J.L., Snook, T., Gay, J.M., Mealey, K.L. 2009. ABCB1-1 Delta (MDR1-1 Delta) genotype is associated with adverse reactions in dogs treated with milbemycin oxime for generalized demodicosis. Vet Dermatol. 2009 Apr;20(2):111-4.

Barnett, K.C., Knight, G.C. 1969. Persistent pupillary membrane and associated defects in the Basenji. Veterinary Record 85:242-245.

Barnett, K.C. 1980. Hereditary cataract in the Welsh springer spaniel. Journal of Small Animal Practice 21: 621-625.

Barnett, K.C. 1985. The diagnosis and differential diagnosis of cataract in the dog. Journal of Small Animal Practice 26:305-316.

Barnhart, K.F., Credille, K.M., Ambrus, A., Dunstan, R.W. 2004. A heritable keratinization defect of the superficial epidermis in norfolk terriers. J Comp Pathol. 130(4):246-54.

Batchelor, D.J., Noble, P.J., Cripps, P.J., Taylor, R.H., McLean, L., Leibl, M.A., German, A.J. 2007. Breed associations for canine exocrine pancreatic insufficiency. J Vet Intern Med. 2007 Mar-Apr;21(2):207-14.

Bedford, P.G.C. 1977. A gonioscopic study of the iridocorneal angle in the English and American breeds of Cocker Spaniel and the Bassett Hound. Journal of Small Animal Practice, 18: 631– 642.

Bedford, P. G. C. 1988. Conditions of the eyelids in the dog. Journal of small animal practice, 29(7), 416-428.

Bedford, P.G.C. 1998. Collie eye anomaly in the Lancashire Heeler. Veterinary Record 143: 354-356.

Bech-Nielsen, S., Haskins, M. E., Reif, J. S., Brodey, R. S., Patterson, D. F., & Spielman, R. (1978). Frequency of osteosarcoma among first-degree relatives of St. Bernard dogs. Journal of the National Cancer Institute, 60(2), 349-353.

Bellenger, C. R. 1980. Perineal hernia in dogs. Australian veterinary journal, 56(9), 434-438.

Bellenger, C R, J E Maddison, G C MacPherson, and J E Ilkiw. 1990. Chronic hypertrophic pyloric gastropathy in 14 dogs. Australian Veterinary Journal 67, no. 9: 317-320.

Bende, B., Nemeth, T. 2004. High prevalence of urate urolithiosis in the Russian black terrier. Vet Rec. 155(8):239-40.

Bennett, D. 1974. Canine dystocia—a review of the literature. Journal of small animal practice, 15(2), 101-117.

Bergstrom, A., Nodtvedt, A., Lagerstedt, A.S., Egenvall, A. 2006. Incidence and breed predilection for dystocia and risk factors for cesarean section in a Swedish population of insured dogs. Vet Surg. 35(8):786-91.

Beuing, R., Janssen, N., Wurster, H., Schmied, O., Fluckiger, M. 2005. The significance of elbow dysplasia (ED) for breeding in Bernese Mountain Dogs in Germany. Schweiz Arch Tierheilkd. 147(11):491-7.

Binder, H., Arnold, S., Schelling, C., Suter, M., Wild, P. 2000. Palmoplantar hyperkeratosis in Irish terriers: evidence of autosomal recessive inheritance. Journal of Small Animal Practice 41(2):52-5.

Bingel, S.A., Sande, R.D. 1994. Chondrodysplasia in five Great Pyrenees Journal of the American Veterinary Medical Association. 205(6):845-8.

Bjerkas, E., Narfstrom, K. 1994. Progressive retinal atrophy in the Tibetan spaniel in Norway and Sweden. Veterinary Record 134(15):377-9.

Bjerkas, E., Haaland, M.B. 1995. Pulverulent nuclear cataract in the Norwegian buhund. Journal of Small Animal Practice 36:471-4.

Bjorck, G., Mair, W., Olsson, S.G., Sourander, P. 1962. Hereditary ataxia in Fox Terriers. Acta Neuropathologia 1 supplement: 45-48

Boari, A., Williams, D.A., Famiglibergamini, P. 1994. Observations on Exocrine Pancreatic Insufficiency in a Family of English Setter Dogs. Journal of Small Animal Practice 35: 247-250.

Booth, M.J. 1998. Atypical dermoid sinus in a chow chow dog. Journal of the South African Veterinary Medical Association 69: 102-104.

Borgarelli, M., Santilli, R. A., Chiavegato, D., D'Agnolo, G., Zanatta, R., Mannelli, A., Tarducci, A. 2006. Prognostic indicators for dogs with dilated cardiomyopathy. Journal of veterinary internal medicine, 20(1), 104-110.

Boudreaux, M. K., Crager, C., Dillon, A. R., Stanz, K., Toivio‐Kinnucan, M. 1994. Identification of an intrinsic platelet function defect in Spitz dogs. Journal of Veterinary Internal Medicine, 8(2), 93-98.

Boudreaux, M.K., Dvam, K., Dillon, A.R., Bourne, C., Scott, M., Schwartz, K.A., Tiovio-Kinnucan, M. 1996. Type 1 Glanzmann's thrombasthenia in a Great Pyrenees dog. Veterinary Pathology 33: 503-511.

Boudreaux, M.K., Catalfamo, J.L. 2001. Molecular and genetic basis for thrombasthenic thrombopathia in otterhounds. American Journal of Veterinary Research. 62(11):1797-804.

Boudreaux, M.K., Martin, M. 2011. P2Y12 receptor gene mutation associated with postoperative hemorrhage in a Greater Swiss Mountain dog. Vet Clin Pathol. 2:202-6.

Bovee, K.C., Joyce, T., Reynolds, R., Segal, S. 1978. The fanconi syndrome in Basenji dogs: a new model for renal transport defects. Science 201:1129-31.

Breitschwerdt, E.B., Waltman, C., Hagstad, H.V., Ochoa, R., McClure, J., Barta, O. 1982. Clinical and epidemiologic characterization of a diarrheal syndrome in Basenji dogs. J Am Vet Med Assoc. 180(8):914-20.

Breitschwerdt, E.B., MacLachlan, N.J., Argenzio, R.A., Hurlbert, S.A., Babineau, C., De Buysscher, E.V. 1991. Gastric acid secretion in Basenji dogs with immunoproliferative enteropathy. J Vet Intern Med. 5(1):34-9.

Breitschwerdt, E.B., Kornegay, J.N., Wheeler, S.J., Stevens, J.B., Baty, C.J. 1992. Episodic weakness associated with exertional lactic acidosis and myopathy in Old English Sheepdog littermates. Journal of the American Veteterinary Medical Association 201: 731-736.

Brenner, O., deLahunta, A., Summers, B.A., Cummings, J,F., Cooper, B.J., Valentine, B.A., Bell, J.S. 1997. Hereditary polioencephalomyelopathy of the Australian Cattle dog. Acta Neuropathologica (Berl) 94: 54-66.

Brenner, K., Harkin, K.R., Andrews, G.A., Kennedy, G. 2009. Juvenile pancreatic atrophy in Greyhounds: 12 cases (1995-2000). J Vet Intern Med 23(1):67-71

Breur, G.J., Zerbe, C.A., Slocombe, R.F., Padgett, G.A., Braden, T.D. 1989. Clinical, radiographic, pathologic, and genetic features of osteochondrodysplasia in Scottish deerhounds. Journal of the American Veterinary Medical Association. 195(5):606-12.

Breur, G.J., Lust, G., Todhunter, R.J. 2001. Genetics of canine hip dysplasia and other orthopaedic traits, in: A. Ruvinskyand, J. Sampson (Eds.), The Genetic of the Dog, CABI, Oxon, UK, pp. 267– 298.

Brewer, G.J., Venta, P.J., Schall, W.D., Yuzbaziyan-Gurkan, V., Li, J. 1998. DNA tests for von Willebrands disease in dobermans, scotties, shelties, and Manchester terriers. Canine Practice 23: 45.

Brix, A.E., Howerth, E.W., McConkie-Rosell, A., Peterson, D., Egnor, D., Wells, M.R., Chen, Y.T. 1995. Glycogen storage disease type Ia in two littermate Maltese puppies. Veterinary Pathology 32: 460-5.

Brons, A.K., Henthorn, P.S., Raj, K., Fitzgerald, C.A., Liu, J., Sewell, A.C., Giger, U. 2013. SLC3A1 and SLC7A9 mutations in autosomal recessive or dominant canine cystinuria: a new classification system. J Vet Intern Med. 27(6):1400-8. doi: 10.1111/jvim.12176.

Brooks, M.B. 1999. A review of canine inherited bleeding disorders: biochemical and molecular strategies for disease characterization and carrier detection. Journal of Heredity 90: 112-118.

Brooks, M.B., MacNguyen, R., Hall, R., Gupta, R., Booth, J.G. 2008. Indirect carrier detection of canine haemophilia A using factor VIII microsatellite markers. Anim Genet. 39(3):278-83.

Brown, N. O., Patnaik, A. K., MacEwen, E. G. 1985. Canine hemangiosarcoma: retrospective analysis of 104 cases. Journal of the American Veterinary Medical Association, 186(1), 56-58.

Bryan, J. N., Henry, C. J., Turnquist, S. E., Tyler, J. W., Liptak, J. M., Rizzo, S. A., ... & Jackson, T. 2006. Primary renal neoplasia of dogs. Journal of veterinary internal medicine, 20(5), 1155-1160.

Buback, J.L., Boothe, H.W., Hobson, H.P. 1996. Surgical treatment of tracheal collapse in dogs: 90 cases (1983-1993). Journal of the American Veterinary Association 208(3):380-4.

Buchanan, J.W. 1992. Causes and prevalence of cardiovascular diseases. In: Kirk RW, Bonagura JD (eds.) Current Veterinary Therapy XI. Philadelphia, PA, pp. 647-655.

Buchanan, J.W., Beardow, A.W., Sammarco, C.D. 1997. Femoral artery occlusion in Cavalier King Charles Spaniels. Journal of the American Veterinary Medical Association 211: 872-874.

Buchanan, J. W. 1999. Prevalence of cardiovascular disorders. Textbook of canine and feline cardiology. 2nd ed. WB Saunders. Philadelphia, 457-470.

Buchanan, J. W., & Patterson, D. F. 2003. Etiology of patent ductus arteriosus in dogs. Journal of veterinary internal medicine, 17(2), 167-171

Burbidge, H.M. 1995. A Review of Laryngeal Paralysis in Dogs. British Veterinary Journal 151: 71-82.

Bussadori, C., Demadron, E., Santilli, R. A., Borgarelli, M. 2001. Balloon valvuloplasty in 30 dogs with pulmonic stenosis: effect of valve morphology and annular size on initial and 1-year outcome. Journal of Veterinary Internal Medicine 15, 553-558.

Callan, M.B., Bennett, J.S., Phillips, D.K., Haskins, M.E., Hayden, J.E., Anderson, J.G., Giger, U. 1995. Inherited platelet delta-storage pool disease in dogs causing severe bleeding: an animal model for a specific ADP deficiency. Thrombosis and Haemostasis 74: 949-953.

Cameron, J.M., Maj, M.C., Levandovskiy, V., MacKay, N., Shelton, G.D., Robinson, B.H. 2007. Identification of a canine model of pyruvate dehydrogenase phosphatase 1 deficiency. Mol Genet Metab. 90(1):15-23.

Campbell, G.A., Crow, D. 2010. Severe zinc responsive dermatosis in a litter of Pharaoh Hounds. J Vet Diagn Invest. 22(4):663-6.

Capen, C. C., Martin, S. L. 1975. Animal model: hyperadrenocorticism (Cushing's-like syndrome and disease in dogs). The American journal of pathology, 81(2), 459.

Carr, J. G., Tobias, K. M., & Smith, L. 2014. Urethral Prolapse in Dogs: A Retrospective Study. Veterinary Surgery.

Carmichael, K.P., Miller, M., Rawlings, C.A., Fischer, A., Oliver, J.E., Miller, B.E. 1996. Clinical, hematologic, and biochemical features of a syndrome in Bernese mountain dogs characterized by hepatocerebellar degeneration. Journal of the American Veterinary Medical Association 208: 1277-1280.

Casal, M.L., Munuve, R.M., Janis, M.A., Werner, P., Henthorn, P.S. 2006. Epilepsy in Irish Wolfhounds. J Vet Intern Med. 20(1):131-5.

Case, L. C., Ling, G. V., Franti, C. E., Ruby, A. L., Stevens, F., Johnson, D. L. 1992. Cystine-containing urinary calculi in dogs: 102 cases (1981-1989). Journal of the American Veterinary Medical Association, 201(1), 129-133.

Caswell, J.L., Nykamp, S.G. 2003. Intradural vasculitis and hemorrhage in full sibling Welsh springer spaniels. Can Vet J. 44(2):137-9.

Catchpole, B., Adams, J.P., Holder, A.L., Short, A.D., Ollier, W.E., Kennedy, L.J. 2013. Genetics of canine diabetes mellitus: Are the diabetes susceptibility genes identified in humans involved in breed susceptibility to diabetes mellitus in dogs? Vet J. 195(2):139-47. doi: 10.1016/j.tvjl.2012.11.013.

Cerundolo, R., Lloyd, D. 1998. 'Alopecia X' in chows, pomeranians and samoyeds. Vet Record 143: 176.

Cerundolo, R. 1999. Symmetrical alopecia in the dog. In Practice (0263841X), 21(7).

Cerundolo, R., Llyod, D.H., MacNeil, P.E. et al. 2000. An analysis of factors underlying hypotrichosis and alopecia in Irish Water Spaniel in the United Kingdom. Veterinary Dermatology 2000; 11: 107-22

Cerundolo, R., Mauldin, E.A., Goldschmidt, M.H., Beyerlein, S.L., Refsal, K.R., Oliver, J.W. 2005. Adult-onset hair loss in Chesapeake Bay retrievers: a clinical and histological study. Vet Dermatol. 16(1):39-46.

Chaudieu, G., Molon-Noblot, S. 2004. Early retinopathy in the Bernese Mountain Dog in France: preliminary observations. Vet Ophthalmol. 2004 May-Jun;7(3):175-84.

Chavkin, M. J., Roberts, S. M., Salman, M. D., Severin, G. A., & Scholten, N. J. 1994. Risk factors for development of chronic superficial keratitis in dogs. Journal of the American Veterinary Medical Association, 204(10), 1630-1634.

Chetboul, V., Tessier, D., Borenstein, N., Delisle, F., Zilberstein, L., Payen, G., Leglaive, E., Franc, B., Derumeaux, G., Pouchelon, J.L. 2003. Familial aortic aneurysm in Leonberg dogs. J Am Vet Med Assoc. 223(8):1159-62, 1129.

Clements, D.N., Fitzpatrick, N., Carter, S.D., Day, P.J. 2007. Cartilage gene expression correlates with radiographic severity of canine elbow osteoarthritis. Vet J. 2007 Oct 13.

Clercx, C., Reichler, I., Peeters, D., McEntee, K., German, A., Dubois, J., Schynts, E., Schaaf-Lafontaine, N., Willems, T., Jorissen, M., Day, M.J. 2003. Rhinitis/Bronchopneumonia syndrome in Irish Wolfhounds.J Vet Intern Med. 17(6):843-9.

Coates, J.R., O'Brien, D.P., Kline, K.L., Storts, R.W., Johnson, G.C., Shelton, G.D., Patterson, E.E., Abbott, L.C. 2002. Neonatal cerebellar ataxia in Coton de Tulear dogs. J Vet Intern Med. 16(6):680-9.

Coates, J.R., March, P.A., Oglesbee, M., Ruaux, C.G., Olby, N.J., Berghaus, R.D., O'Brien, D.P., Keating, J.H., Johnson, G.S., Williams, D.A. 2007. Clinical characterization of a familial degenerative myelopathy in Pembroke Welsh Corgi dogs. J Vet Intern Med. 21(6):1323-31

Collins, B.K., Collier, L.L., Johnson, G.S., Shibuya, H., Moore, C.P., da Silva, Curiel. J.M. 1992. Familial cataracts and concurrent ocular anomalies in chow chows. Journal of the American Veterinary Medical Association, 10:1485-91.

Collier, L., McCalla, T., Moor, C.P. 1989. Anterior lens luxation in the Queensland Heeler (Australian Cattle Dog).Transactions of the American College Veterinary Ophtalmologists 20:185.

Comhaire, F.H., Snaps, F. 2008. Comparison of two canine registry databases on the prevalence of hip dysplasia by breed and the relationship of dysplasia with body weight and height. Am J Vet Res. 2008 Mar;69(3):330-3.

Conaway, D.H., Padgett, G.A., Nachreiner, R.F. 1985. The familial occurrence of lymphocytic thyroiditis in borzoi dogs. American Journal of Medical Genetics. 22(2):409-14.

Cooley, P.L., Dice, P.F. 1990. Corneal dystrophy in the dog and cat. Veterinary Clinics of North America, Small Animal Practice. 20(3):681-92.

Coppens, A.G., Resibois, A., Poncelet, L. 2000. Bilateral deafness in a maltese terrier and a great pyrenean puppy: inner ear morphology. J Comp Pathol. 122(2-3):223-8.

Coppieters, E., Samoy, Y., Pey, P., Waelbers, T., & Van Ryssen, B. 2012. Medial compartment disease in a young Large Munsterlander. Vlaams Diergeneeskundig Tijdschrift, 81(2), 88-92.

Corcoran, K. A., Koch, S. A. 1993. Uveal cysts in dogs: 28 cases (1989-1991). Journal of the American Veterinary Medical Association, 203(4), 545-546.

Corcoran, K.A., Koch, S.A., Peiffer, R.L. Jr. 1994. Primary Glaucoma in the Chow Chow. Progress in Veterinary and Comparative Ophthalmology 4:193

Cordy, D.R., Snelbaker, H.A. 1952. Cerebellar hypoplasia and degeneration in a family of airedale dogs. J Neuropathol Exp Neurol. 11(3):324-8.

Cork, L.C., Morris, J.M., Olson, J.L., Krakowka, S., Swift, A.J., Winkelstein, J.A. 1991. Membranoproliferative Glomerulonephritis in Dogs with a Genetically Determined Deficiency of the Third Component of Complement. Clinical Immunology and Immunopathology 60: 455-470.

Cotrell, B.D., Barnett, K.C. 1988. Primary glaucoma in the Welsh springer spaniel. Journal of Small Animal Practice 29: 185-199.

Court, M.H. 1999. Anaesthesia of the sighthound. Clin Tech Small Anim Pract. 1999 Feb;14(1):38-43.

Cowan, L.A., Hertzke, D.M., Fenwick, B.W., Andreasen, C.B. 1997. Clinical and clinicopathologic abnormalities in greyhounds with cutaneous and renal glomerular vasculopathy: 18 cases (1992-1994) J Am Vet Med Assoc. 210(6):789-93.

Coward, P.S. 1989. A Familial Alopecia. Veterinary Record 125: 609-610.

Coyne, B.E., Fingland, R.B. 1992. Hypoplasia of the trachea in dogs: 103 cases (1974-1990). J Am Vet Med Assoc. 201(5):768-72.

Crispin, S., Warren, C. 2008. Hereditary eye disease and the BVA/KC/ISDS Eye Scheme: an update. In practice 30: 2-14.

Cummings, J.F., deLahunta, A. 1978. Hereditary myelopathy of Afghan hounds, a myelinolytic disease. Acta Neuropathologica 42: 173-181.

Cummings, J.F., Cooper, B.J., deLahunta, A., van Winkle, T.J. 1981. Canine inherited hypertrophic neuropathy. Acta Neuropathologica 53: 137-143.

Cummings, J.F., de Lahunta, A., Winn, S.S. 1981. Acral mutilation and nociceptive loss in English pointer dogs. A canine sensory neuropathy. Acta Neuropathologica (Berlin) 53(2):119-27.

Cummings, J. F., De Lahunta, A., Braund, K. G., Mitchell Jr, W. J. 1983. Hereditary sensory neuropathy. Nociceptive loss and acral mutilation in pointer dogs: canine hereditary sensory neuropathy. The American journal of pathology, 112(1), 136.

Cummings, J.F., Summers, B.A., de Lahunta, A., Lawson, C. 1986. Tremors in Samoyed pups with oligodendrocyte deficiencies and hypomyelination. Acta Neuropathol (Berl). 71(3-4):267-77.

Curtis, R., Barnett, K.C. 1980. Primary lens luxation in the dog. Journal of Small Animal Practice 21:657.

Curtis, R., Barnett, K.C. 1981. Canine adenovirus-induced ocular lesions in the Afghan hound. Cornell Vet. 71(1):85-95.

Curtis, R., Barnett, K.C. 1983. The 'blue eye' phenomenon. Vet Rec. 1112(15):347-53.

Curtis, R., Barnett, K. C., Startup, F. G. 1983. Primary lens luxation in the miniature bull terrier. Veterinary record, 112(14), 328-330.

D’Anna, N., Sapienza, J. S., Guandalini, A., Guerriero, A. 2007. Use of a dermal biopsy punch for removal of ectopic cilia in dogs: 19 cases. Veterinary ophthalmology, 10(1), 65-67.

da Silva, E. O., Green, K. T., Wasques, D. G., Chaves, R. O., Faria dos Reis, A. C., & Bracarense, A. P. F. R. L. 2012. Malignant Pilomatricoma in a Dog. Journal of comparative pathology, 147(2), 214-217.

de Lahunta A. 1983. Veterinary Neuroanatomy and Clinical Neurology, 2nd ed. Philadelphia, PA: WB Saunders.

Davis, D. 1958. Canine Pyloric Stenosis. Iowa State University Veterinarian, 20(2), 14.

Day, M. J. 1994. An immunopathological study of deep pyoderma in the dog. Research in veterinary science, 56(1), 18-23.

De Bosschere, H., Bos, M., Ducatelle, R., Bhatti, S., Van Ham, L. 2002. Spongiform degeneration of the white matter in the central nervous system of Australian cattle dog littermates. Vlaams Diergeneeskundig Tijdschrift 71: 145-148.

Delauche, A.J., Cuddon, P.A., Podell, M., Devoe, K., Powell, H.C., Shelton, G.D. 1998. Nemaline rods in canine myopathies: 4 case reports and literature review. Journal of Veterinary Internal Medicine 12: 434-430.

DeNovo, R. C. 2003. Diseases of the stomach. Handbook of Small Animal Gastroenterology, 160.

Dice, P.F. 1976. Corneal dystrophy in the Airedale. Proc Am Coll Vet Ophthalmol. 7:36.

Dietz, H.H. 1985. Retinal dysplasia in dogs--a review (in Norwegian). Nordisk Veterinaermedicin 37:1-9.

Dodds, W.J., Moynihan, A.C., Fisher, T.M., Trauner, D.B. 1981. The frequencies of inherited blood and eye diseases as determined by genetic screening programs. Journal of the American Animal Hospital Association 17: 697-704.

Dodds, W.J. 1989. Hemostasis. In: Kaneko, J.J. (ed) Clinical biochemistry of domestic animals, 4th edn. Academic Press, San Diego. pp.274-315.

Done, S.H., Drew, R.A., Robins, G.M., Lane, J.G. 1975. Hemivertebra in the dog: clinical and pathological observations. Veterinary Record 96(14):313-7.

Downs, L.M., Bell, J.S., Freeman, J., Hartley, C., Hayward, L.J., Mellersh, C.S.2013. Late-onset progressive retinal atrophy in the Gordon and Irish Setter breeds is associated with a frameshift mutation in C2orf71. Anim Genet. 2:169-77. doi: 10.1111/j.1365-2052.2012.02379.

Drögemüller, C., Becker, D., Kessler, B., Kemter, E., Tetens, J., Jurina, K., Jäderlund, K.H., Flagstad, A., Perloski, M., Lindblad-Toh, K., Matiasek, K.2010. A deletion in the N-myc downstream regulated gene 1 (NDRG1) gene in Greyhounds with polyneuropathy. PLoS One. 6: e11258.

Dukes-McEwan, J., Borgarelli, M., Tidholm, A., Vollmar, A. C.,Häggström, J. 2003. Proposed guidelines for the diagnosis of canine idiopathic dilated cardiomyopathy. Journal of Veterinary Cardiology, 5(2), 7-19.

Duval, J. M., Budsberg, S. C., Flo, G. L., Sammarco, J. L. 1999. Breed, sex, and body weight as risk factors for rupture of the cranial cruciate ligament in young dogs. Journal of the American Veterinary Medical Association, 215(6), 811-814.

Edmonds, L., Stewart, R.W., Selby, L. 1972. Cleft lip and palate in Boston Terrier pups. Veterinary Medicine and Small Animal Clinician 67: 1219-1222.

Edwards, D. F., Kennedy, J. R., Toal, R. L., Maddux, J. M., Barnhill, M. A., Daniel, G. B. 1989. Kartagener's syndrome in a chow chow dog with normal ciliary ultrastructure. Veterinary Pathology Online, 26(4), 338-340.

Edwards, D.F., Patton, C.S., Kennedy, J.R. 1992. Primary ciliary dyskinesia in the dog. Problems in Veterinary Medicine 4:291-319.

Egenvall, A., Hagman, R., Bonnett, B. N., Hedhammar, A., Olson, P., & Lagerstedt, A. S. 2001. Breed risk of pyometra in insured dogs in Sweden. Journal of veterinary internal medicine, 15(6), 530-538.

Ekesten, B., Torrang, I. 1995. Heritability of the depth of the opening of the ciliary cleft in Samoyeds. American Journal of Veterinary Research. 56(9): 1138–43.

Ekenstedt, K.J., Patterson, E.E., Minor, K.M., Mickelson, J.R. 2011. Candidate genes foridiopathic epilepsy in four dog breeds. BMC Genet. 12:38.

Ellinwood, N.M., Wang, P., Skeen, T., Sharp, N.J., Cesta, M., Decker, S., Edwards, N.J., Bublot, I., Thompson, J.N., Bush, W., Hardam, E., Haskins, M.E., Giger, U. 2003. A model of mucopolysaccharidosis IIIB (Sanfilippo syndrome type IIIB): N-acetyl-alpha-D-glucosaminidase deficiency in Schipperke dogs. J Inherit Metab Dis. 26(5):489-504.

Ellison, G., Halling, K.B. 2004. Atypical pectus excavatum in two Welsh terrier littermates. J Small Anim Pract. 45(6):311-4

Eneroth, A., Linde-Forsberg, C., Uhlhorn, M., Hall, M. 1999. Radiographic pelvimetry for assessment of dystocia in bitches: a clinical study in two terrier breeds. J Small Anim Pract 40:257–264.

Engstrom, D. 1966. Tyrosinase deficiency in the Chow Chow. In: Current Veterinary Therapy II (ed Kirk, R.W.), WB Saunders, Phildelphia, pp. 352.

Eriksen, K., Grondalen, J. 1984. Familial renal disease in soft-coated Wheaton terriers. Journal of Small Animal Practice 25:489-500

Evans, K.M., Adams, V.J. 2010a. Mortality and morbidity due to gastric dilatation-volvulus syndrome in pedigree dogs in the UK. J Small Anim Pract. Jul;51(7):376-81.

Evans, K. M., Adams, V. J. 2010b. Proportion of litters of purebred dogs born by caesarean section. Journal of small animal practice, 51(2), 113-118.

Fall, T., Hamlin, H. H., Hedhammar, Å., Kämpe, O., Egenvall, A. 2007. Diabetes mellitus in a population of 180,000 insured dogs: incidence, survival, and breed distribution. Journal of veterinary internal medicine, 21(6), 1209-1216.

Farrow, B.R.H., Malik, R. 1981. Hereditary myotonia in the Chow Chow. Journal of Small Animal Practice 22: 451-465.

Fasanella, F.J., Shivley, J.M., Wardlaw, J.L., Givaruangsawat, S. 2010. Brachycephalic airway obstructive syndrome in dogs: 90 cases (1991-2008). J Am Vet Med Assoc. 237(9):1048-51.

Fascetti, A.J., Reed, J.R., Rogers, Q.R., Backus, R.C. 2003. Taurine deficiency in dogs with dilated cardiomyopathy: 12 cases (1997-2001). J Am Vet Med Assoc. 223(8): 1137-41.

Feldman, E.C., Nelson, R.W. 1966. Canine and Feline Endocrinology and Reproduction, 2^nd^ edition, WB Saunders, Philidelphia.

Ferm, K., Björnerfeldt, S., Karlsson, A., Andersson, G., Nachreiner, R., Hedhammar, A. 2009. Prevalence of diagnostic characteristics indicating canine autoimmune lymphocytic thyroiditis in giant schnauzer and hovawart dogs. J Small Anim Pract. 4: 176-9.

Finnigan, D.F., Hanna, W.J., Poma, R., Bendall, A.J. 2007. A novel mutation of the CLCN1 gene associated with myotonia hereditaria in an Australian cattle dog. J Vet Intern Med. 21(3):458-63.

Flegel, T., Matiasek, K., Henke, D., Grevel, V. 2007. Cerebellar cortical degeneration with selective granule cell loss in Bavarian mountain dogs. J Small Anim Pract.48(8):462-5.

Freeman, B., Evans, V. B., and McEwan, N. R. 2013. Canine hip dysplasia in Irish water spaniels: two decades of gradual improvement. Veterinary Record, 173(3), 72-72.

Fogh, J.M., Fogh, I.T. 1988. Inherited coagulation disorders. Veterinary Clinics of North America 18: 231-243.

Forman, P., De Risio, L., Mellersh, C.S. 2013. Missense mutation in CAPN1 is associated with spinocerebellar ataxia in the Parson Russell Terrier dog breed. PLoS One.8(5):e64627. doi: 10.1371/journal.pone.0064627.

Fossum, T.W., Birchard, S.J., Jacobs, R.M. 1986. Chylothorax in 34 dogs. J Am Vet Med Assoc. 188(11):1315-8.

Fox, P. R., Sisson, D., Moise, N. S. 1999. Textbook of Canine and Feline Cardiology: Principles and Clinical Practice. 2nd edn. Philadelphia, PA, USA: Saunders. p xvi, pp. 955.

Fujise, H., Hishiyama, N., Ochiai, H. 1997. Heredity of red blood cells with high K and low glutathione (HK/LG) and high K and high glutathione (HK/HG) in a family of Japanese Shiba Dogs. Exp Anim. 46(1):41-6.

Fyfe, J.C., Giger, U., Hall, C.A., Jezyk, P.F., Klumpp, S.A.  et al.  1991. Inherited selective intestinal cobalamin malabsorption and cobalamin deficiency in dogs. Pediatr Res 29:24-31.

Fyfe, J.C., Al-Tamimi, R.A., Castellani, R.J., Rosenstein, D., Goldowitz, D., Henthorn, P.S. 2010. Inherited neuroaxonal dystrophy in dogs causing lethal, fetal-onset motor system dysfunction and cerebellar hypoplasia. J Comp Neurol. 18:3771-84.

Gabriel, A., Poncelet, L., Van Ham, L., Clercx, C., Braund, K.G., Bhatti, S., Detilleux, J., Peeters, D. 2006. Laryngeal paralysis-polyneuropathy complex in young related Pyrenean mountain dogs. J Small Anim Pract.47(3):144-9.

Gandolfi, B., Liu, H., Griffioen, L., Pedersen, N.C. 2013. Simple recessive mutation in ENAM is associated with amelogenesis imperfecta in Italian Greyhounds. Anim Genet. 5: 569-578. doi: 10.1111/age.12043.

Gaudet, A.D. 1985. Retrospective study of 128 cases of canine dystocia. J Am Anim Hosp Assoc 21:813–818.

Gaughan, K.R., Bruyette, D.S. 2001. Thyroid function testing in Greyhounds. Am J Vet Res 62(7):1130-3.

Gelain, M. E.; Tutino, G. F.; Pogliani, E.; Bertazzolo, W. 2010. Macrothrombocytopenia in a group of related Norfolk terriers. Veterinary Record 167 (13):493-494

Gelatt, K.N., Whitley, R.D., Lavach, J.D., Barrie, K.P., Williams, L.W. 1979. Cataracts in Chesapeake Bay Retrievers. Journal of the American Veterinary Medical Association. 175(11):1176-8.

Gelatt, K.N., Powell, N.G., Huston, K. 1981. Inheritance of microphthalmia with coloboma in the Australian shepherd dog. American Journal of Veterinary Research. 42(10):1686-90.

Gelatt, K.N., MacKay, E.O. 2004. Prevalence of breed-related glaucomas in pure-bred dogs in North America. Vet Ophthalmol. 7(2):97-111.

Gelatt, K. N., MacKay, E. O. 2005. Prevalence of primary breed‐related cataracts in the dog in North America. Veterinary ophthalmology, 8(2), 101-111.

Gelatt, K.N. 2007. Veterinary Opthalamology, 4^th^ edition, Blackwell Publishing, Oxford, UK.

Gelatt, Kirk N., Dennis E. Brooks, and Maria E. Kallberg. 2008. The canine glaucomas. Essentials of Veterinary Ophthalmology. 2: 155-187.

Gelzer, A.R., Downs, M.O., Newell, S.M., Mahaffey, M.B., Fletcher, J., Latimer, K.S. 1997. Accessory lung lobe torsion and chylothorax in an Afghan hound. J Am Anim Hosp Assoc. 33(2):171-6.

Genevois, J.P., Remy, D., Viguier, E., Carozzo, C., Collard, F., Cachon, T., Maitre, P., Fau, D. 2008. Prevalence of hip dysplasia according to official radiographic screening, among 31 breeds of dogs in France. Vet Comp Orthop Traumatol. 21(1):21-4.

Geyer, J., Doring, B., Godoy, J.R., Leidolf, R., Moritz, A., Petzinger, E. 2005. Frequency of the nt230 (del4) MDR1 mutation in Collies and related dog breeds in Germany. J Vet Pharmacol Ther.28(6):545-51.

Giger, U., Noble, N.A. 1991. Determination of erythrocyte pyruvate kinase deficiency in Basenjis with chronic hemolytic anemia. J Am Vet Med Assoc. 198(10):1755-61.

Giger, U., Smith, B.F., Woods, C.B., Patterson, D.F., Stedman, H. 1992. Inherited phosphofructo-kinase deficiency in an American cocker spaniel. Journal of the American Veterinary Medical Association 201: 1569-71.

Giger, U. 2003. Hereditary blood disorders. In:Proceedings, World Small Animal Veterinary World Congress.

Gillard, M., Cadieu, E., De Brito, C., Abadie, J., Vergier, B., Devauchelle, P., Degorce, F., Dréano, S., Primot, A., Dorso, L., Lagadic, M., Galibert, F., Hédan, B., Galibert, M.D., André, C. 2014. Naturally occurring melanomas in dogs as models for non-UV pathways of human melanomas. Pigment Cell Res. (1):90-102. doi: 10.1111/pcmr.12170.

Glickman, L.T., Glickman, N.W., Schellenberg, D.B., Raghavan, M., Lee, T.L. 2000. Incidence of and breed-related risk factors for gastric dilatation-volvulus in dogs. Journal of the American Veterinary Medical Association 216: 40-45.

Ginja, M. M. D., Silvestre, A. M., Colaço, J., Gonzalo-Orden, J. M., Melo-Pinto, P., Orden, M. A., ... & Ferreira, A. J. 2009. Hip dysplasia in Estrela mountain dogs: Prevalence and genetic trends 1991–2005. The Veterinary Journal, 182(2), 275-282.

Gracis, M., Keith, D., Vite , C.H. 2000. Dental and craniofacial findings in eight miniature schnauzer dogs affected by myotonia congenita: preliminary results. J Vet Dent. 17(3): 119-27.

Grahn, B.H., Philibert, H., Cullen, C.L., Houston, D.M., Semple, H.A., Schmutz, S.M. 1998. Multifocal retinopathy of Great Pyrenees dogs. Veterinary Ophthalmology. 1(4):211-221.

Grahn, B.H., Sandmeyer, L.S., Breaux, C.B. 2006. Multifocal bullous retinopathy of coton de Tulear dogs.Can Vet J. 47(9):929-30.

Gramer, I., Leidolf, R., Döring, B., Klintzsch, S., Krämer, E.M., Yalcin, E., Petzinger, E., Geyer, J. 2010. Breed distribution of the nt230(del4) MDR1 mutation in dogs. Vet J. Jul 21.

Greco, D.S., Feldman, E.C., Petersen, M.E., Turner, J.L., Hodges, C.M., Shipman, L.W. 1991. Congenital hypothyroid dwarfism in a family of giant schnauzers. J Vet Intern Med. 5(2):57-65

Green, R.A., Lantz, G.C. 1978. Factor VIII deficiency in an Old English Sheepdog. Journal of the American Animal Hospital Association 14: 394-398.

Gregory, B.L., Shelton, G.D., Bali, D.S., Chen, Y.T., Fyfe, J.C. 2007. Glycogen storage disease type IIIa in curly-coated retrievers. J Vet Intern Med.21(1):40-6.

Grenn, H. H., & Lindo, D. E. 1969. Hemivertebrae with severe kypho-scoliosis and accompanying deformities in a dog. The Canadian Veterinary Journal, 10(8), 214.

Grondalen, J. 1973. Malformation of the elbow joint in an Afghan hound litter. J Small Anim Pract. 14(2):83-9.

Groondalen, J. 1981. A generalized chondropathy of joint cartilage leading to deformity of the elbow joints in a litter of Newfoundland dogs. Journal of Small Animal Practice. 22(8):523-38.

Goggin, J. E., Li, A.S., Franti, C.E. 1970. Canine intervertebral disc: Characterisation by age, sex, breed and anatomic site of involvement. Am. J. Vet. Res. 31,1687 1692.

Golden, J.G., Banknieder, A.R., Bruestle, M.E. 1980. Hemophilia in a Great Pyrenees. Modern Veterinary Practice 61(8):671-4.

Goldschmidt, M. H., Shofer, F. S. 1992. Skin tumors of the dog and cat. Pergamon Press Ltd., Oxford, UK.

Goldstein, R.E., Atwater, D.Z., Cazolli, D.M., Goldstein, O., Wade, C.M., Lindblad-Toh, K. 2007. Inheritance, mode of inheritance, and candidate genes for primary hyperparathyroidism in Keeshonden. J Vet Intern Med.21(1):199-203.

Gonsalves-Hubers, T. 2005. Pemphigus erythematosus in a chow chow. Can Vet J. 46(10):925-7.

Gould, D., Pettitt, L., McLaughlin, B., Holmes, N., Forman, O., Thomas, A., Ahonen, S., Lohi, H., O'Leary, C., Sargan, D., Mellersh, C. 2011. ADAMTS17 mutation associated with primary lens luxation is widespread among breeds. Vet Ophthalmol. Aug 3.

Gu, W., Brooks, M., Catalfamo, J., Ray, J., Ray, K. 1999. Two distinct mutations cause severe hemophilia B in two unrelated canine pedigrees. Thrombosis and Haemostasis 82:1270-5.

Gu, Y.C., Bauer, T.R. Jr., Ackermann, M.R., Smith, C.W., Kehrli, M.E. Jr., Starost, M.F., Hickstein, D.D. 2004. The genetic immunodeficiency disease, leukocyte adhesion deficiency, in humans, dogs, cattle, and mice. Comp Med. 54(4):363-72.

Guilford, W. G., Center, S. A., Strombeck, D. R., Williams, D. A., Meyer, D. J. 1996. Acute haemorrhagic enteropathy/haemorrhagic gastroenteritis. In: Strombeck's small animal gastroenterology, 3^rd^ edn. (ed Guilford, W.G.), WB Saunders, Phildelphia, pp. 477-480.

Gunby, J.M., Hardie, R.J., Bjorling, D.E. 2004. Investigation of the potential heritability of persistent right aortic arch in Greyhounds. J Am Vet Med Assoc. 224(7):1120-2, 1111.

Gurguis, G.N.M., Klein, E., Mefford, I.N., Uhde, T.W. 1990. Biogenic Amines Distribution in the Brain of Nervous and Normal Pointer Dogs - A Genetic Animal Model of Anxiety. Neuropsychopharmacology 3: 297-303.

Gutierrez‐Quintana, R., Guevar, J., Stalin, C., Faller, K., Yeamans, C., Penderis, J. 2014. A Proposed radiographic classification scheme for congenital thoracic vertebral malformaitons in brachycephalic “screw-tailed” dog breeds. Veterinary Radiology & Ultrasound. doi: 10.1111/vru.12172.

Guziewicz, K.E., Zangerl, B., Lindauer, S.J., Mullins, R.F., Sandmeyer, L.S., Grahn, B.H., Stone, E.M., Acland, G.M., Aguirre, G.D. 2007. Bestrophin gene mutations cause canine multifocal retinopathy: a novel animal model for best disease. : Invest Ophthalmol Vis Sci. 48(5):1959-67.

Hagman, R., Lagerstedt, A.S., Hedhammar, Å., Egenvall, A. 2011. A breed-matched case-control study of potential risk-factors for canine pyometra. Theriogenology. Apr 15;75(7):1251-7.

Hall, S.J., Wallace, M.E. 1996. Canine epilepsy: a genetic counselling programme for keeshonds.Veterinary Record 138: 358-60.

Hamil, S.L. 1990. Eye problems in the bloodhound. Pure Bred Dogs/ American Kennel Gazette. Accessed on web 3rd Jan 2010.

Hansen,H. J. 1952. A pathological-anatomical study on disc degeneration in the dog. Acta Orthop. Scand. Suppl. 11,1 120.

Hargis, A.M., Brignac, M., Al Bagdadi, F. et al. 1991. Black hair follicular dysplasia in black and white Saluki dogs: differentiation from colour mutant alopecia in the Doberman Pinscher by microscopic examination of hairs. Veterinary Dermatology. 2: 69-83.

Hargis, A. M., Ihrke, P. J., Spangler, W. L., Stannard, A. A. 1992. A retrospective clinicopathologic study of 212 dogs with cutaneous hemangiomas and hemangiosarcomas. Veterinary Pathology Online, 29(4), 316-328.

Harlos, C. 2010. Genetic associations between DLA class II and Symmetrical lupoid onychodystrophy in giant schnauzers and bearded collie. Fakulteten för Veterinärmedicin och husdjursvetenskap Institutionen för husdjursgenetik. (PhD Thesis) Uppsula.

Harper, R.C. 1978. Congenital black hair follicular dysplasia in bearded collie puppies.Veterinary Record 102: 87.

Hayashi, K., Manley, P.A., Muir, P. 2004. Cranial cruciate ligament pathophysiology in dogs with cruciate disease: a review. J Am Anim Hosp Assoc. 40(5):385-90.

Hayes, H. M., Fraumeni, J. F. 1974. Chemodectomas in dogs: epidemiologic comparisons with man. Journal of the National Cancer Institute, 52(5), 1455-1458.

Hayes, H.M., Priester, W.A. Jr, Pendergrass, T.W. 1975. Occurrence of nervous-tissue tumors in cattle, horses, cats and dogs. Int J Cancer. 1975 Jan 15;15(1):39-47.

Hayes, H. M. 1984. Breed associations of canine ectopic ureter: a study of 217 female cases. Journal of small animal practice, 25(8), 501-504.

Hayes, H.M. Jr., Wilson, G.P., Pendergrass, T.W., Cox, V.S. 1985. Canine cryptorchidism and subsequent testicular neoplasia: Case control study with epidemiological update. Teratology 32:51-56.

Hayes ,H.M., Wilson, G.P. 1986. Hospital incidence of hypospadias in dogs in North America. Vet Rec. 118(22): 605-7.

Hazlett, M.J., Smith-Maxie, L.L., de Lahunta, A. 2005. A degenerative encephalomyelopathy in 7 Kuvasz puppies. Can Vet J. 46(5):429-32

Heitmann, M., Hamann, H., Brahm, R., Grußendorf, H., Rosenhagen, C. U., Distl, O. 2005. Analysis of prevalence of presumed inherited eye diseases in Entlebucher Mountain Dogs. Veterinary ophthalmology, 8(3), 145-151.

Hendricks, J.C. 1992. Brachycephalic airway syndrome. Vet Clin North Am Small Anim Pract. 22(5):1145-53.

Heinrich, C.L., Lakhani, K.H., Featherstone, H.J., Barnett, K.C. 2006. Cataract in the UK Leonberger population. Vet Ophthalmol. 5:350-356.

Henthorn, P.S., Liu, J., Gidalevich, T., Fang, J., Casal, M.L., Patterson, D.F., Giger, U. 2000. Canine cystinuria: polymorphism in the canine SLC3A1 gene and identification of a nonsense mutation in cystinuric Newfoundland dogs. Human Genet.ics 107(4):295-303.

Henthorn, P.S., Gilbert-Gregory, S., Steinberg, S.A. 2004. Non-syndromic Inherited Deafness in Pointer Dogs. (Abstract). 2nd International Conference “Advances in Canine & Feline Genomics”. pp29.

Herrtage, E., Houlton, J.E. 1979. Collapsing Clumber spaniels. Veterinary Record 105(14):334.

Higgins, R.J., LeCouteur, R.A., Kornegay, J.N., Coates, J.R. 1998. Late onset progressive spinocerebellar degeneration in Brittany spaniels. Acta Neuropathologica 96: 97-1101.

Hill, C. 2006. Primary epilepsy in the Italian spinone.Vet Rec. 159(11):368.

Hiraide, F., Paparella, M.M. 1988. Histopathology of the temporal bones of deaf dogs. Auris Nasus Larynx. 1988;15(2):97-104.

Ho, L. K., Troy, G. C., Waldron, D. R. 2011. Clinical outcomes of surgically managed ectopic ureters in 33 dogs. Journal of the American Animal Hospital Association, 47(3), 196-202.

Hoffmann, I., Guziewicz, K.E., Zangerl, B., Aguirre, G.D., Mardin, C.Y. 2012. Canine multifocal retinopathy in the Australian Shepherd: a case report. Vet Ophthalmol. Mar 20. doi: 10.1111/j.1463-5224.2012.01005.x.

Holt, P. E., Thrusfield, M. V. 1993. Association in bitches between breed, size, neutering and docking, and acquired urinary incontinence due to incompetence of the urethral sphincter mechanism. Veterinary Record, 133(8), 177-180.

Holt, P. E., & Moore, A. H. 1995. Canine ureteral ectopia: an analysis of 175 cases and comparison of surgical treatments. Veterinary record, 136(14), 345-349.

Hoppe, F., Svalastoga, E. 1980. Temporomandibular dysplasia in American Cocker Spaniels. J. Small Anim Pract. 21(12): 675-8.

Hoppe, A., Denneberg, T. 2001. Cystinuria in the dog: Clinical studies during 12 years of medical treatment. J Vet Intern Med, 15:361-367.

Houlton, J.E., Herrtage, M.E. 1980. Mitochondrial myopathy in the Sussex spaniel. Veterinary Record 106(9):206.

Hubert, B., Braun, J.P., de La Farge, F., Magnol, J.P. 1987. Hypertriglyceridemia in 2 related dogs. Companion Animal Practice 1:33-35.

Hughes, A.M., Nelson, R.W., Famula, T.R., Bannasch, D.L. 2007. Clinical features and heritability of hypoadrenocorticism in Nova Scotia Duck Tolling Retrievers: 25 cases (1994-2006). J Am Vet Med Assoc. 231(3):407-12.

Hultin Jäderlund, K., Baranowska Körberg, I., Nødtvedt, A. 2011. Inherited polyneuropathy in leonberger dogs. Vet Intern Med. (5):997-1002.

Hunt, G.B. 2004. Effect of breed on anatomy of portosystemic shunts resulting from congenital diseases in dogs and cats: a review of 242 cases. Aust Vet J. 82(12):746-9.

Ihrke, P.J., Stannard, A.A., Ardans, A.A., Griffin, C.E. 1985. Pemphigus foliaceus in dogs: a review of 37 cases. Journal of the American Veterinary Medical Association 186(1):59-66.

James, R.W. 1991. Persistent pupillary membrane in basenji dogs. Vet Rec. 128(12):287-8.

Jansen, J.H., Arnesen, K. 1990. Oxalate nephropathy in a Tibetan spaniel litter. A probable case of primary hyperoxaluria. Journal of Comparative Pathology 103(1):79-84.

Janutta, V., Distl, O. 2006. Inheritance of canine hip dysplasia: review of estimation methods and of heritability estimates and prospects on further developments. Dtsch Tierarztl Wochenschr. 13(1):6-12.

Jenkins, W.L., van Dyk, E., McDonald, C.B. 1976. Myasthenia gravis in a Fox Terrier litter. Journal of the South African Veterinary Assocociation 47: 59-62.

Jeserevics, J., Viitmaa, R., Cizinauskas, S., Sainio, K., Jokinen, T.S., Snellman, M., Bellino, C., Bergamasco, L. 2007. Electroencephalography findings in healthy and Finnish Spitz dogs with epilepsy: visual and background quantitative analysis. J Vet Intern Med. 21(6):1299-306.

Johnson, G.F., Sternlieb, I., Twedt, D.C., Grushoff, P.S., Scheinberg , I. 1980. Inheritance of copper toxicosis in Bedlington terriers. Am J Vet Res. 41(11):1865-6.

Johnston, G.R., Feeney, D.A., O'Brien, T.D., Klausner, J.S., Polzin, D.J., Lipowitz, A.J., Levine, S.H., Hamilton, H.B., Haynes, J.S. 1984. Recurring lung lobe torsion in three Afghan hounds. J Am Vet Med Assoc., 184(7):842-5.

Johnson, G.S., Turrentine, M.A., Kraus, K.H. 1988. Canine von Willebrand's disease. Veterinary Clinics of North America 18: 195-223.

Johnson & Patterson. 2003. http://www.canine-epilepsy.net/cerc.html;

Jokinen, T. S., Rusbridge, C., Steffen, F., Viitmaa, R., Syrjä, P., De Lahunta, A., ... & Cizinauskas, S. 2007a. Cerebellar cortical abiotrophy in Lagotto Romagnolo dogs. Journal of small animal practice, 48(8), 470-473.

Jokinen, T.S., Metsähonkala, L., Bergamasco, L., Viitmaa, R., Syrjä, P., Lohi, H., Snellman, M., Jeserevics, J., Cizinauskas, S. 2007b. Benign familial juvenile epilepsy in Lagotto Romagnolo dogs. J Vet Intern Med. 21(3):464-71.

Jones, B.R. ,Richards, R.B. 1977. Myelomalacia in Afghan hounds, Aust. Vet. J. 53:452-3

Jones, B.R., Brennan, S., Mooney, C.T., Callanan, J.J., McAllister, H., Guo, L.T., Martin, P.T., Engvall, E.,

Shelton, G.D. 2004. Muscular dystrophy with truncated dystrophin in a family of Japanese Spitz dogs. J Neurol Sci. 217(2):143-9.

Kammermann, B., Gmur, J., Stunzi, H. 1971. Afibrinogenaemia in the dog. Zentralblatt fur Veterinarmedizin 18: 192-205.

Karlsson, E.K., Sigurdsson, S., Ivansson, E., Thomas, R., Elvers, I., Wright, J., Howald, C., Tonomura, N., Perloski, M., Swofford, R., Biagi, T., Fryc, S., Anderson, N., Courtay-Cahen, C., Youell, L., Ricketts, S.L., Mandlebaum, S., Rivera, P., von Euler, H., Kisseberth, W.C., London, C.A., Lander, E.S., Couto, G., Comstock, K., Starkey, M.P., Modiano, J.F., Breen, M., Lindblad-Toh, K. 2013. Genome-wide analyses implicate 33 loci in heritable dog osteosarcoma, including regulatory variants near CDKN2A/B. Genome Biol. 12:R132.

Karmi, N., Brown, E.A., Hughes, S.S., McLaughlin, B., Mellersh, C.S., Biourge, V., Bannasch, D.L. 2010. Estimated frequency of the canine hyperuricosuria mutation in different dog breeds. J Vet Intern Med. 6:1337-42.

Kaswan, R. L., Salisbury, M. A. 1990. A new perspective on canine keratoconjunctivitis sicca. Treatment with ophthalmic cyclosporine. The Veterinary Clinics of North America. Small Animal Practice, 20(3), 583-613.

Kathmann, I., Jaggy, A., Busato, A., Bartschi, M., Gaillard, C. 1999. Clinical and genetic investigations of idiopathic epilepsy in the Bernese mountain dog. Journal of Small Animal Practice 40(7):319-25.

Kato, K., Sasaki, N., Matsunaga, S., Nishimura, R., Ogawa, H. 2006a. Incidence of canine glaucoma with goniodysplasia in Japan: a retrospective study. J Vet Med Sci.68(8):853-8.

Kato, K., Sasaki, N., Matsunaga, S., Mochizuki, M., Nishimura, R., Ogawa, H. 2006b. Possible association of glaucoma with pectinate ligament dysplasia and narrowing of the iridocorneal angle in Shiba Inu dogs in Japan. Veterinary ophthalmology, 9(2):71-75.

Katz, M.L., Khan, S., Awano, T., Shahid, S.A., Siakotos, A.N., Johnson, G.S. 2005. A mutation in the CLN8 gene in English Setter dogs with neuronal ceroid-lipofuscinosis. Biochem Biophys Res Commun. 327(2):541-7.

Katz, M.L., Farias, F.H., Sanders, D.N., Zeng, R., Khan, S., Johnson, G.S., O'Brien, D.P. 2011. A missense mutation in canine CLN6 in an Australian shepherd with neuronal ceroid lipofuscinosis. J Biomed Biotechnol.2011:198042.

Kazmierski, K. J., Ogilvie, G. K., Fettman, M. J., Lana, S. E., Walton, J. A., Hansen, R. A., ... & Chavey, S. 2001. Serum zinc, chromium, and iron concentrations in dogs with lymphoma and osteosarcoma. Journal of Veterinary Internal Medicine, 15(6), 585-588.

Kemp, C., Thiele, H., Dankof, A., Schmidt, G., Lauster, C., Fernahl, G., Lauster, R. 2009. Cleft Lip and/or Palate With Monogenic Autosomal Recessive Transmission in Pyrenees Shepherd Dogs. Cleft Palate Craniofac J. 46(1):81-8.

Kent, M., Knowles, K., Glass, E., deLahunta, A., Braund, K., Alroy, J. 1999. Motor neuron abiotrophy in a saluki. Journal of the American Animal Hospital Association. 35(5):436-9.

Kidd, L.B., Salavaggione, O.E., Szumlanski, C.L., Miller, J.L., Weinshilboum, R.M., Trepanier, L. 2004. Thiopurine methyltransferase activity in red blood cells of dogs. J Vet Intern Med. 18(2):214-8.

Kijas, J.W., Miller, B.J., Pearce-Kelling, S.E., Aguirre, G.D., Acland, G.M. 2003. Canine models of ocular disease: outcross breedings define a dominant disorder present in the english mastiff and bull mastiff dog breeds. J Hered. 94(1):27-30.

Kim, J.H., Kang, K.I., Sohn, H.J., Woo, G.H., Jean, Y.H., Hwang, E.K. 2005. Color-dilution alopecia in dogs. J Vet Sci. 6(3):259-61.

Kimmel, S. E., Ward, C. R., Henthorn, P. S., Hess, R. S. 2002. Familial insulin-dependent diabetes mellitus in Samoyed dogs. Journal of the American Animal Hospital Association, 38(3), 235-238.

Kimura, T., Ohshima, S., Doi, K. 1993. The inheritance and breeding results of hairless descendants of Mexican hairless dogs. Laboratory animals, 27(1), 55-58.

Kirberger, R. M., Fourie, S. L. 1998. Elbow dysplasia in the dog: pathophysiology, diagnosis and control: review article. Journal of the South African Veterinary Association, 69(2), p-43.

Kirberger, R.M., Stander , N. 2007. Incidence of canine elbow dysplasia in South Africa. J S Afr Vet Assoc. 78(2):59-62.

Kittleson, M.D., Keene, B., Pion, P.D., Loyer, C.G. 1997. Results of the multicenter Spaniel trial (MUST): taurine and carnitine responsive dilated cardiomyopathy in American Cocker spaniels with decreased plasma taurine concentration. Journal of Veterinary Internal Medicine 11: 204-211.

Kittleson, M.D., Kienle, R.D. 1998. Small Animal Cardiovascular Medicine. Mosby, St. Louis, MO, pp 603.

Kramer, J.W., Klaassen, J.K., Baskin, D.G., Prieur, D.J., Rantanen, N.W., Robinette, J.D., Graber, W.R., Rashti, L. 1988. Inheritance of diabetes mellitus in Keeshond dogs. American Journal of Veterinary Research 49: 428-31.

Krohne, S. 2001. Inherited cataracts in dogs. CERF news http://www.vmdb.org/aug01.html#dxspot.

Krontveit, R. I., Trangerud, C., Sævik, B. K., Skogmo, H. K., & Nødtvedt, A. 2012. Risk factors for hip-related clinical signs in a prospective cohort study of four large dog breeds in Norway. Preventive veterinary medicine, 103(2), 219-227.

Krotscheck, U., Adin, C.A., Hunt, G.B., Kyles, A.E., Erb, H.N. 2007. Epidemiologic factors associated with the anatomic location of intrahepatic portosystemic shunts in dogs. Vet Surg. 36(1):31-6.

Klarenbeek, S., Gerritzen-Bruning, M.J., Rozemuller, A.J., van der Lugt, J.J. 2007. Canine X-linked muscular dystrophy in a family of Grand Basset Griffon Vendéen dogs. J Comp Pathol. 137(4):249-52.

Klein, E., Steinberg, S.A., Weiss, S.R.B., Matthews, D.M., Uhde, T.W. 1988. The relationship between genetic deafness and fear- related behaviors in nervous pointer dogs. Physiology and Behavior 43: 307-312.

Kleiter, M., Högler, S., Kneissl, S., Url, A., Leschnik, M. 2011. Spongy degeneration with cerebellar ataxia in Malinois puppies: a hereditary autosomal recessive disorder? J Vet Intern Med. May-Jun;25(3):490-6.

Kniazev, S.P., Kulikova, A.V., Aksenovich ,T.I., Aul'chenko, IuS. 2003. Oligodontia and its inheritance in Kerry blue terrier. Genetika.39(6):805-12.

Knowler, C., Giger, U., Dodds, W.J., Brooks, M. 1994. Factor XI deficiency in Kerry blue terriers. Journal of the American Veterinary Medical Association 205: 1557-1561.

Knowler, S.P., McFadyen, A.K., Freeman, C., Kent, M., Platt, S.R., Kibar, Z., Rusbridge, C. 2014. Quantitative analysis of Chiari-like malformation and syringomyelia in the Griffon Bruxellois dog. PLoS One 9(2):e88120. doi: 10.1371/journal.pone.0088120.

Knowles ,K,. Alroy, J., Castagnaro, M., Raghavan ,S,S., Jakowski, R.M., Freden, G.O. 1993. Adult -Onset Lysosomal Storage Disease in a Schipperke Dog - Clinical, Morphological and Biochemical Studies. Acta Neuropathologica 86: 306-312.

Koch, S.A. 1972. Cataracts in interrelated Old English Sheepdogs. Journal of the American Veterinary Medical Association 160: 299.

Kropatsch, R., Petrasch-Parwez, E., Seelow, D., Schlichting, A., Gerding ,W.M., Akkad, D.A., Epplen, J.T., Dekomien, G. 2010. Generalized progressive retinal atrophy in the Irish Glen of Imaal Terrier is associated with a deletion in the ADAM9 gene. Mol Cell Probes. 6:357-63.

Kwochka, K.W., Rademakers, A.M. 1989. Cell proliferation kinetics of epidermis, hair follicles, and sebaceous glands of cocker spaniels with idiopathic seborrhea. Am J Vet Res. 50(11):1918-22.

Kyöstilä, K., Lappalainen, A.K., Lohi, H. 2013. Canine chondrodysplasia caused by a truncating mutation in collagen-binding integrin alpha subunit 10.PLoS One. 9:e75621. doi: 10.1371/journal.pone.0075621.

LaFond, E., Breur, G.J., Austin, C.C. 2002. Breed susceptibility for developmental orthopedic diseases in dogs. J Am Anim Hosp Assoc. 38(5):467-77.

Laratta, L.J., Sims, M.H., Brooks, D.E. 1988. Progressive retinal degeneration in the Australian Cattle Dog. Transactions of the American College Veterinary Ophtalmologists 19: 9.

Lau, R.E. 1977. Inherited premature closure of the distal ulna physis. Journal of the American Animal Hospital Association 13: 609-12.

Lawson, D. D. 1973. Canine distichiasis. Journal of Small Animal Practice, 14(8), 469-478.

Leppanen, M., Martenson, J., Maki, K. 2001. Results of ophthalmologic screening examinations of German Pinschers in Finland--a retrospective study. Vet Ophthalmol. 4(3):165-9.

Li, F.Y., Cuddon, P.A., Song, J., Wood, S.L., Patterson, J.S., Shelton, G.D., Duncan, I.D. 2006. Canine spongiform leukoencephalomyelopathy is associated with a missense mutation in cytochrome b. Neurobiol Dis. 21(1): 33-42.

Lightfoot, R.M., Cabral, L., Gooch, L., Bedford, P.G., Boulton, M.E. 1996. Retinal pigment epithelial dystrophy in Briard dogs. Res Vet Sci.60(1):17-23.

Lit, L., Belanger, J.M., Boehm, D., Lybarger, N., Oberbauer, A.M. 2013. Differences in behavior and activity associated with a poly(a) expansion in the dopamine transporter in Belgian malinois. PLoS One. 23;8(12):e82948. doi:10.1371/journal.pone.0082948.

Littman, M.P., Dambach, D.M., Vaden, S.L., Giger, U. 2000. Familial protein-losing enteropathy and protein-losing nephropathy in Soft Coated Wheaten Terriers: 222 cases (1983-1997). Journal of Veterinary Internal Medicine 14: 68-80.

Ling, G.V., Stabenfeldt, G.H., Comer, K.M., Gribble, D.H., Schechter, R.D. 1979. Canine hyperadrenocorticism: pretreatment clinical and laboratory evaluation of 117 cases. J Am Vet Med Assoc. 174(11):1211-5.

Lipsitz D, Berry JL, Shelton GD. 1999. Inherited predisposition to myasthenia gravis in Newfoundlands. J Am Vet Med Assoc.215(7): 956-8.

Lobo, L., Carvalheira, J., Canada, N., Bussadori, C., Gomes, J.L., Faustino, A.M.R. 2010. Histologic Characterization of Dilated Cardiomyopathy in Estrela Mountain Dogs. 2010. Vet Pathol 47:637 originally published online 27 April 2010. DOI: 10.1177/0300985810364511.

Lohmann, B., Klesen, S. 1997. Cataract and microphthalmia in a litter of Russian Terriers [German]. Praktische Tierarzt 78: 981ff.

Lothrop, Jr., C. D. 1988. Pathophysiology of canine growth hormone-responsive alopecia. The Compendium on continuing education for the practicing veterinarian (USA).

Lowe, J.K., Kukekova, A.V., Kirkness, E.F., Langlois, M.C., Aguirre, G.D., Acland, G.M., Ostrander, E.A. 2003. Linkage mapping of the primary disease locus for collie eye anomaly. Genomics. 82(1):86-95.

Lowe, R.C., King, M.C.A. 2004. Electroretinogram findings and retinal appearance in a confirmed case of ivermectin toxicity in a Lakeland Terrier. British Association of Veterinary Ophthalmologists, winter meeting.

Lubbes, D., Mandigers, P.J., Heuven, H.C., Teske, E. 2009. Incidence of gastric carcinoma in Dutch Tervueren shepherd dogs born between 1991 and 2002. Tijdschr Diergeneeskd. 134(14-15):606-10.

Lynch, G.L. 2007. Ophthalmic examination findings in a group of retired racing Greyhounds. Vet Ophthalmol. 10(6):363-7.

Mahaffey, M.B., Yarbrough, K.M., Munnell, J.F. 1978. Focal loss of pigment in the Belgian Tervuren dog. J Am Vet Med Assoc. 173(4):390-6.

Mandigers, P.F., Van Nes, J.J., Knol, B.W., Ubbink, G.J., Gruys, E. 1993. Hereditary necrotizing myelopathy in Kooiker dogs. Research in Veterinary Science 54: 118-123.

March, P. A., J. R. Coates, R. J. Abyad, D. A. Williams, D. P. O'Brien, N. J. Olby, J. H. 2009. Keating, and M. Oglesbee. "Degenerative myelopathy in 18 Pembroke Welsh corgi dogs." Veterinary Pathology Online 46, (2):241-250.

Marfe, G., De Martino, L., Tafani, M., Irno-Consalvo, M., Pasolini, M.P., Navas, L., Papparella, S., Gambacurta, A., Paciello, O. 2012. A multicancer-like syndrome in a dog characterized by p53 and cell cycle-checkpoint kinase 2 (CHK2) mutations and sirtuin gene (SIRT1) down-regulation. Res Vet Sci. 1:240-5.

Marmor, M., Willeberg, P., Glickman, L. T., Priester, W. A., Cypess, R. H., Hurvitz, A. I. 1982. Epizootiologic patterns of diabetes mellitus in dogs. American journal of veterinary research, 43(3), 465-470.

Martin, C.L., Leipold, H.W., 1974. Aphakia and multiple ocular defects in Saint Bernard puppies. Veterinary Medicine Small Animal Clinics 69:448.

Martin, M.W., Stafford Johnson, M.J., Celona, B. 2008. Canine dilated cardiomyopathy: a retrospective study of signalment, presentation and clinical findings in 369 cases. Martin MW, Stafford Johnson MJ, Celona B. J Small Anim Pract. 2008 Nov 13.

Mason, T.A. 1976. Persistent pupillary membrane in the Basenji. Aust Vet J. 52(8):343-4.

Mason, N.J., Day, M.J. 1996. Renal amyloidosis in related english foxhounds. Journal of Small Animal Practice 37: 255-260.

Matic, S. E. 1988. Congenital heart disease in the dog. Journal of Small Animal Practice, 29(12), 743-759.

Mausberg, E.M., Drogemuller, C., Rufenacht, S., Welle, M., Roosje, P., Suter, M., Leeb, T. 2007. Inherited alopecia X in Pomeranians. Dtsch Tierarztl Wochenschr. (4):129-34.

Mausberg, E.M., Drögemüller, C., Dolf, G., Rüfenacht, S., Welle, M., Leeb, T. 2008. Exclusion of patched homolog 2 (PTCH2) as a candidate gene for alopecia X in Pomeranians and Keeshonden. Vet Rec. 163(4):121-3.

Mazzucchelli, S., Vaillant, M.D., Wéverberg, F., Arnold-Tavernier, H., Honegger, N., Payen, G., Vanore, M., Liscoet, L., Thomas, O., Clerc, B., Chahory, S. 2012. Retrospective study of 155 cases of prolapse of the nictitating membrane gland in dogs. Vet Rec. 170:443. Doi: 10.1136/vr.100587.

McCaw, D., Aronson, E. 1984. Congenital cardiac disease in dogs. Mod Vet Pract. 65(7):509-12

McGrotty, Y.L., Ramsey, I.K., Knottenbelt, C.M. 2003. Diagnosis and management of hepatic copper accumulation in a Skye terrier. J Small Anim Pract. 44(2):85-9.

Mellersh, C.S., Graves, K.T., McLaughlin, B., Ennis, R.B., Pettitt ,L., Vaudin, M., Barnett, K.C. 2007. Mutation in HSF4 associated with early but not late-onset hereditary cataract in the Boston Terrier. J Hered. 98(5):531-3. Epub 2007 Jul 4.

Mellersh, C.S., McLaughlin, B., Ahonen, S., Pettitt, L., Lohi, H., Barnett, K.C. 2009. Mutation in HSF4 is associated with hereditary cataract in the Australian Shepherd. Vet Ophthalmol. 12(6):372-8.

Melniczek, J.R., Dambach, D., Prociuk, U., Jezyk, P.F., Henthorn, P.S., Patterson, D.F., Giger, U. 1999. Sry-negative XX sex reversal in a family of Norwegian Elkhounds. J Vet Intern Med. 13(6):564-9.

Merveille, A.C., Battaille, G., Billen, F., Deleuze, S., Fredholm, M., Thomas, A., Clercx, C., Lequarré, A.S. 2014. Clinical findings and prevalence of the mutation associated with primary ciliary dyskinesia in old english sheepdogs.J Vet Intern Med. (3):771-8. doi: 10.1111/jvim.12336.

Meyers-Wallen, V.N., Palmer, V.L., Acland, G.M., Hershfield, B. 1995. Sry-negative XX sex reversal in the American cocker spaniel dog. Molecular Reproduction and Development 41(3):300-5.

Miller, L.M., Hegreberg, G.A., Prieur, D.J., et al., 1984. Inheritance of congenital myasthenia gravis in smooth fox terrier dogs. Journal of Heredity, 75, 163-166.

Miller, M.A., Dunstan, R.W. 1993. Seasonal flank alopecia in boxers and airedale terriers: 24 cases (1985-1992).Journal of the American Veterinary Medical Association 203(11):1567-72.

Miller, M.S., Tilley, L.P., Smith, F.W.K. 2000. Disorders of cardiac rhthym. In: Birchard SJ, Sherding RG (eds). Saunders Manual of Small Animal Practice 2nd ed., W.B. Saunders, Philadelphia, PA. pp. 470-485.

Miller, S. A., Hohenhaus, A. E., Hale, A. S. 2004. Case-control study of blood type, breed, sex, and bacteremia in dogs with immune-mediated hemolytic anemia. Journal of the American Veterinary Medical Association, 224(2), 232-235.

Miller, P. E. 2008. Breed predisposition to eye disorders. Slatter's Fundamentals of Veterinary Ophthalmology, 442.

Mills, J. N., Day, M. J., Shaw, S. E., Penhale, W. J. 1985. Autoimmune haemolytic anaemia in dogs. Australian veterinary journal, 62(4), 121-123.

Minkus, G., Breuer, W., Wanke, R., Reusch, C., Leuterer, G., Brem, G., Hermanns, W. 1994. Familial nephropathy in Bernese mountain dogs. Veterinary Pathology 31: 421-8.

Minor, K.M., Patterson, E.E., Keating, M.K., Gross, S.D., Ekenstedt, K.J., Taylor, S.M., Mickelson, J.R. 2011. Presence and impact of the exercise-induced collapse associated DNM1 mutation in Labrador retrievers and other breeds. Vet J. 189(2):214-9.

Moore, F.M., Thornton, G.W. 1983. Telangiectasia of Pembroke Welsh Corgi dogs. Veterinary Pathology. 20(2):203-8.

Morgan, R.V. 1989. Vogt-Koyanagi-Harada Syndrome in Humans and Dogs. Compendium on Continuing Education for the Practicing Veterinarian, 11(10): 1211-1218.

Morgan, R.V., Duddy, J.M., McClurg, K. 1993. Prolapse of the gland of the third eyelid in dogs: a retrospective study of 89 cases (1980 to 1990). J Am Anim Hosp Assoc 29:56–60.

Morrison, W. B., Wilsman, N. J., Fox, L. E., Farnum, C. E. 1987. Primary ciliary dyskinesia in the dog. Journal of Veterinary Internal Medicine, 1(2), 67-74.

Mortari, A. C., Rahal, S. C., Vulcano, L. C., da Silva, V. C., & Volpi, R. S. 2009. Use of radiographic measurements in the evaluation of dogs with medial patellar luxation. The Canadian Veterinary Journal, 50(10), 1064.

Mulvihull, J.J., Priester, W.A. 1973. Congenital heart disease in dogs: Epidemiologic similarities to man. Teratology 7:73-78

Munyard, K.A., Sherry, C.R., Sherry, L. 2007. A retrospective evaluation of congenital ocular defects in Australian Shepherd dogs in Australia.Vet Ophthalmol. 10(1):19-22.

Murphy, C.J., Marfurt, C.F., McDermott, A., Bentley, E., Abrams, G.A., Reid, T.W., Campbell, S. 2001. Spontaneous chronic corneal epithelial defects (SCCED) in dogs: clinical features, innervation, and effect of topical SP, with or without IGF-1. Invest Ophthalmol Vis Sci. 42(10):2252-61.

Nachreiner, R.F., Refsal, K.R., Graham, P.A., Bowman, M.M. 2002. Prevalence of serum thyroid hormone autoantibodies in dogs with clinical signs of hypothyroidism. J Am Vet Med Assoc. 220(4):466-71.

Narfström, K., Wrigstad, A., Ekesten, B., et al., 2007. Neuronal ceroid lipofuscinosis: clinical and morphogenic findings in nine affected Polish Owczarek Nizinny (PON) dogs. Veterinary Ophthalmalology, 10(2):11-120.

Neath, P.J., Brockman, D.J., King, L.G. 2000. Lung lobe torsion in dogs: 22 cases (1981-1999). J Am Vet Med Assoc. 217(7):1041-4

Neer, T.M., Dial, S.M., Pechman, R., Wang, P., Oliver, J.L., Giger, U. 1995. Clinical Vignette. Mucopolysaccharidosis VI in a miniature pinscher. Journal of Veterinary Internal Medicine 9: 429-43.

Nibe, K., Kita, C., Morozumi, M., Awamura, Y., Tamura, S., Okuno, S., Kobayashi, T., Uchida, K. 2007. Clinicopathological features of canine neuroaxonal dystrophy and cerebellar cortical abiotrophy in Papillon and Papillon-related dogs. J Vet Med Sci. 69(10):1047-52.

Niskanen, M., Thrusfield, M. V. 1998. Associations between age, parity, hormonal therapy and breed, and pyometra in Finnish dogs. Veterinary Record, 143(18), 493-498.

Noureddine, C., Harder, R., Olby, N. J., Spaulding, K., & Brown, T. 2004. Ultrasonographic Appearance of Dandy Walker‐Like Syndrome in a Boston Terrier. Veterinary Radiology & Ultrasound, 45(4), 336-339.

Nowacka, J., Nizanski, W., Klimowicz, M., Dzimira, S., & Switonski, M. 2005. Lack of the SOX9 gene polymorphism in sex reversal dogs (78, XX; SRY negative). Journal of heredity, 96(7), 797-802.

Oberbauer, A.M., Benemann, K.S., Belanger, J.M., Wagner, D.R., Ward, J.H., Famula, T.R. 2002. Inheritance of hypoadrenocorticism in bearded collies. Am J Vet Res. 63(5):643-7.

Oberbauer, A., Bell, J., Belanger, J., Famula, T. 2006. Genetic evaluation of Addison's disease in the Portuguese Water Dog. BMC Vet Res. 2006 May 2;2:15.

O'Brien, J. 1975. Spontaneous laryngeal disease in the canine. Laryngoscope. 85(12 pt 1):2023-5.

O'Brien, D.P., Barshop, B.A., Faunt, K.K., Johnson, G.C., Gibson, K.M., Shelton, G.D. 1999. Malonic aciduria in Maltese dogs: normal methylmalonic acid concentrations and malonyl-CoA decarboxylase activity in fibroblasts. Journal of Inherited Metabolic Diseases. 22(8):883-90.

O’Brien, D.P., Taylor, J.F., Johnson, G.S., Johnson, G.C. 2004. Mapping of Multi System Atrophy and Hairlessness in Chinese Crested and Kerry Blue Terriers. (Abstract). 2nd International Conference “Advances in Canine & Feline Genomics”. pp56.

O'Brien, D. P., Johnson, G. S., Schnabel, R. D., Khan, S., Coates, J. R., Johnson, G. C., Taylor, J. F. 2005. Genetic mapping of canine multiple system degeneration and ectodermal dysplasia loci. Journal of heredity, 96(7), 727-734.

O’Brien, D.P., Katz, M.L. 2008. Neuronal ceroid lipofuscinosis in 3 Australian shepherd littermates. J Vet Intern Med. 22(2):472-5.

Ogburn, P.N., Peterson, M., Jeraj, K. 1981. Multiple cardiac anomalies in a family of Saluki dogs. Journal of the American Veterinary Medical Association. 179(1):57-63.

Oliveira, P., Domenech, O., Silva, J., Vannini, S., Bussadori, R., & Bussadori, C. 2011. Retrospective review of congenital heart disease in 976 dogs. Journal of veterinary internal medicine, 25(3), 477-483.

Olivero, D., Turba, M.E., Gentilini, F. 2011. Reduced diversity of immunoglobulin and T-cell receptor gene rearrangements in chronic inflammatory gastrointestinal diseases in dogs. Vet Immunol Immunopathol. 2011 Aug 19.

Onions, D. E. 1984. A prospective study of familial canine lymphosarcoma. Journal of the National Cancer Institute 72, 909-912

Ordeix, L., Bardagí, M., Scarampella, F., Ferrer, L., Fondati, A. 2009. Demodex injai infestation and dorsal greasy skin and hair in eight wirehaired fox terrier dogs. Vet Dermatol. 20(4):267-72.

Orthopedic Foundation for animals statistics, (2003) web pages - patellar luxation http://www.offa.org/ patluxstatbreed.html

Osborne, C.A., Sanderson, S.L., Lulich, J.P., Bartges, J.W., Ulrich, L.K., Koehler, L.A., Bird, K.A., Swanson, L.L. 1999. Canine cystine urolithiasis. Cause, detection, treatment, and prevention. Veterinary Clinics of North America, Small Animal Practice 29: 193-211.

Oshima, Y., Bjerkas, E., Peiffer, R.L. Jr. 2004. Ocular histopathologic observations in Norwegian Elkhounds with primary open-angle, closed-cleft glaucoma. Vet Ophthalmol. 7(3):185-8.

Oswald, G.P., Orton, E.C. 1993. Patent ductus arteriosus and pulmonary hypertension in related Pembroke Welsh corgis. Journal of the American Veterinary Medical Association. 202(5):761-4.

Ovrebo, B. J., Hanssen, I., Moen, T. 2001. Antinuclear antibodies (ANA) in Gordon setters with symmetrical lupoid onychodystrophy and black hair follicular dysplasia. Acta Veterinaria Scandinavia 42(3):323-9.

Padgett, G. A., Madewell, B. R., Keller, E. T., Jodar, L., & Packard, M. 1995. Inheritance of histiocytosis in Bernese mountain dogs. Journal of Small Animal Practice, 36(3), 93-98.

Palmer, A. C., Medd, R. K., Wilkinson, G. T. 1984. Spinal cord degeneration in hound ataxia. Journal of small animal practice, 25(3), 139-148.

Palmer, A.C., Blakemore, W.F., Wallace, M.E., Wilkes, M.K., Herrtage, M.E., Matic, S.E. 1987. Recognition of trembler, a hypomyelinating condition in the Bernese mountain dog Veterinary Record 120: 609-612.

Paltrinieri, S., Comazzi, S., Ceciliani, F., Prohaska, R., & Bonfanti, U. 2007. Stomatocytosis of Standard Schnauzers is not associated with stomatin deficiency. The Veterinary Journal, 173(1), 200-203.

Panciera, D. L. 1994. Hypothyroidism in dogs: 66 cases (1987-1992). Journal of the American Veterinary Medical Association, 204(5), 761-767.

Paradis, M., Scott, D. W. Breton, L. 1989. Squamous cell carcinoma of the nail bed in three related giant schnauzers. Veterinary Record, 125(12), 322-324.

Parker, H.G., Kukekova, A.V., Akey, D.T., Goldstein, O., Kirkness, E.F., Baysac ,KC.,. Mosher, D.S., Aguirre, G.D., Acland, G.M., Ostrander, E.A. 2007. Breed relationships facilitate fine-mapping studies: a 7.8-kb deletion cosegregates with Collie eye anomaly across multiple dog breeds. Genome Res. 17(11):1562-71.

Parker, H.G., VonHoldt, B.M., Quignon, P., Margulies, E.H., Shao, S., Mosher, D.S., Spady, T.C., Elkahloun, A., Cargill, M., Jones, P.G., Maslen, C.L., Acland, G.M., Sutter, N.B., Kuroki, K., Bustamante, C.D., Wayne, R.K., Ostrander, E.A. 2009. An expressed fgf4 retrogene is associated with breed-defining chondrodysplasia in domestic dogs. Science. 325(5943):995-8.

Parker, H.G., kanene, K., Cadieu, E., Lark, K.G., Ostrander, E.A. 2010. An insertion in the RSPO2 gene correlates with improper coat in the Portuguese water dog. J Hered. 101(5):612-7.

Parker, H.G., Kilroy-Glynn, P. 2012. Myxomatous mitral valve disease in dogs: does size matter? J Vet Cardiol. 1:19-29.

Parry, B.W., Holloway, S.A., Birch, D.J., Mackin, A.J. 1988. Haemophilia A in a cocker spaniel dog. Australian Veterinary Journal 65(10):326-7.

Patterson, D. F. 1968. Epidemiologic and genetic studies of congenital heart disease in the dog. Circulation Research, 23(2), 171-202.

Patterson, D.F. 1989. Hereditary congenital heart defects in dogs. Journal of Small Animal Practice 30: 153-165.

Pedersen, N.C., Liu, H., Greenfield, D.L., Echols, L.G. 2011 Multiple autoimmune diseases syndrome in Italian Greyhounds: Preliminary studies of genome-wide diversity and possible associations within the dog leukocyte antigen (DLA) complex. Vet Immunol Immunopathol. Nov 29.

Peeters, M.E., Ubbink, G.J. 1994. Dysphagia-associated muscular dystrophy: a familial trait in the bouvier des Flandres. Veterinary Record 134(17):444-6.

Peterson, M.E., Kintzer, P.P., Kass, P.H. 1996. Pretreatment clinical and laboratory findings in dogs with hypoadrenocorticism: 225 cases (1979-1993). Journal of the American Veterinary Medical Association 208(1):85-91.

Petersen-Jones, S.M., Entz, D.D., Sargan, D.R. 1999. cGMP phosphodiesterase-alpha mutation causes progressive retinal atrophy in the Cardigan Welsh corgi dog. Investigative Ophthalmology and Visual Science 40: 1637-44.

Petrick, S.W. 1996. The incidence of eye disease in dogs in a veterinary academic hospital:1772 cases. J S Afr. Vet Assoc. 67(3):108-110.

Pfahler, S., Menzel, J., Brahm, R., Rosenhagen, C.U., Hafemeister, B., Schmidt, U., Sinzinger, W., Distl, O. 2014. Prevalence and formation of primary cataracts and persistent hyperplastic tunica vasculosa lentis in the German Pinscher population in Germany. Vet Ophthalmol. doi: 10.1111/vop.12167.

Philipp, U., Menzel, J., Distl, O. 2011. A rare form of persistent right aorta arch in linkage disequilibrium with the DiGeorge critical region on CFA26 in German Pinschers. J Hered. 102 Suppl 1:S68-73.

Phillips, J.C., Stephenson, B., Hauck, M., Dillberger, J. 2007. Heritability and segregation analysis of osteosarcoma in the Scottish deerhound. Genomics 90(3):354-63.

Piek ,C.J., Hazewinkel, H.A., Wolvekamp, W.T., Nap, R.C., Mey, B.P. 1996. Long-term follow-up of avascular necrosis of the femoral head in the dog. Journal of Small Animal Practice 37 (1):12-8.

Poncet, C.M., Dupre, G.P., Freiche, V.G., Estrada, M.M., Poubanne, Y.A., Bouvy, B.M. 2005. Prevalence of gastrointestinal tract lesions in 73 brachycephalic dogs with upper respiratory syndrome. J Small Anim Pract. 46(6):273-9.

Poncet, C. M., Dupre, G. P., Freiche, V. G., & Bouvy, B. M. 2006. Long‐term results of upper respiratory syndrome surgery and gastrointestinal tract medical treatment in 51 brachycephalic dogs. Journal of small animal practice, 47(3), 137-142.

Post, K., Van Haaften, B., Okkens, A. C. 1991. Vaginal hyperplasia in the bitch: Literature review and commentary. The Canadian Veterinary Journal, 32(1), 35.

Presthus, J., Nordstoga, K. 1993. Congenital myopathy in a litter of Samoyed dogs. Progress in Veterinary Neurology 4: 37-40.

Priester, W.A. 1974. Canine progressive retinal atrophy: Occurrence by age, breed and sex. American Journal of Veterinary Research 35:571.

Ramos-Vara, J.A., Beissenherz, M.E., Miller, M.A., Johnson, G.C., Pace, L.W., Fard, A., Kottler, S.J. 2000. Retrospective study of 338 canine oral melanomas with clinical, histologic, and immunohistochemical review of 129 cases. Veterinary Pathology 37(6):597-608.

Ramos-Vara, J.A., Miller, M.A., Ojeda, J.L., Reid, R., Craft, D., Watson, G.L. 2004. Glomerulocystic Kidney Disease in a Belgian Malinois Dog: An Ultrastructural, Immunohistochemical, and Lectin-binding Study. Ultrastruct Pathol. 28(1):33-42.

Reetz, I., Stecker, M., Wegner, W. 1977. Audiometrische befunde in einer Merlezuchte. (Audiometric findings in dachshund (merle gene carrier) Deutche Tierarztliche Wochenschrift 84:273-7.

Reist-Marti, S. B., Dolf, G., Leeb, T., Kottmann, S., Kietzmann, S., Butenhoff, K., & Rieder, S. (2012). Genetic evidence of subaortic stenosis in the Newfoundland dog. Veterinary Record, 170(23), 597-597.

Rohdin, C., Lüdtke, L., Wohlsein, P., Jäderlund, K.H. 2010. New aspects of hereditary ataxia in smooth-haired fox terriers. Vet Rec. 18:557-60.

Richtsmeier, J.T., Sack, G.H. Jr, Grausz, H.M., Cork, L.C. 1994. Cleft palate with autosomal recessive transmission in Brittany spaniels. Cleft Palate Craniofacial Journal 31(5):364-71.

Roberts, S.R., Helper, L.C. 1972. Cataracts in Afghan Hounds. Journal of the American Veterinary Medical Association. 160: 427-32.

Robins, G., Innes, J. 2006. The elbow. In: BSAVA Manual of Canine and Feline Musculoskeletal Disorders (eds Houlton J.E.F., Cook, J.L., Innes, J.F., et al.), British Small Animal Veterinary Association, Gloucester, pp. 249-280.

Romagnoli, S. E. 1991. Canine cryptorchidism. The Veterinary clinics of North America. Small animal practice, 21(3), 533-544.

Roux, P., Stich, H., Schawalder, P. 2011. Multiple tooth resorption in an Italian greyhound. Schweiz Arch Tierheilkd. 6:281-286.

Rubin, L.F. 1989. Inherited eye diseases in purebred dogs. Williams & Wilkins, Balitmore, U.S.A.

Rubin, L.F., Nelson, E.J., Sharp, C.A. 1991. Collie eye anomaly in Australian Shepherd dogs. Prog in Veterinary and Comparative Ophthalmology 1:105.

Rudd, R. G., Whitehair, J. G., Margolis, J. H. 1990. Results of management of osteochondritis dissecans of the humeral head in dogs: 44 cases (1982 to 1987). Journal of the American Animal Hospital Association, 26(2), 173-178.

Rugbjerg, H., Proschowsky, H.F., Ersboll, A.K., Lund, J.D. 2003. Risk factors associated with interdog aggression and shooting phobias among purebred dogs in Denmark. Prev Vet Med. 58(1-2):85-100.

Rühli, M. B., Spiess, B. M. 1995. Goniodysplasia in the Bouvier des Flandres. Schweizer Archiv fur Tierheilkunde, 138(6):307-311.

Russell, N. J., Bond, K. A., Robertson, I. D., Parry, B. W., Irwin, P. J. 2006. Primary hypoparathyroidism in dogs: a retrospective study of 17 cases. Australian veterinary journal, 84(8), 285-290.

Ruth, D. 2012. Interpretation and use of BVA/KC hip scores in dogs. In Practice 34.4: 178-194.

Sack, G.H. Jr, Cork, L.C., Morris, J.M., Griffin, J.W., Price, D.L. 1984. Autosomal dominant inheritance of hereditary canine spinal muscular atrophy. Ann Neurol. 15(4):369-73.

Sams, R.A., Muir, W.W. 1988. Effects of phenobarbital on thiopental pharmacokinetics in greyhounds. Am J Vet Res. 2:245-9.

Sanchez, R. F., Innocent, G., Mould, J., Billson, F. M. 2007. Canine keratoconjunctivitis sicca: disease trends in a review of 229 cases. Journal of small animal practice, 48(4), 211-217.

Sargan, D.R., Withers, D., Pettitt, L., Squire, M., Gould, D.J., Mellersh, C.S. 2007. Mapping the mutation causing lens luxation in several terrier breeds. J Hered. 98(5):534-8.

Schmutz, S.M., Moker, J.S., Clark, E.G., Shewfelt, R. 1998. Black hair follicular dysplasia, an autosomal recessive condition in dogs. Canadian Veterinary Journal 39: 644-6.

Scholten-Sloof, B.E., Knol, B.W., Rijnberk, A., Mol, J.A., Middleton, D.J., Ubbink, G.J. 1992. Pituitary-dependent hyperadrenocorticism in a family of Dandie Dinmont terriers. Journal of Endocrinology 135(3):535-42.

Schultheiss, P. C. 2006. Histologic features and clinical outcomes of melanomas of lip, haired skin, and nail bed locations of dogs. Journal of veterinary diagnostic investigation, 18(4), 422-425.

Schulze, C., Meyer, H.P., Blok, A.L., Schipper, K., van den Ingh, T.S. 1998. Renal dysplasia in three young adult Dutch kooiker dogs. Veterinary Quarterly 20(4):146-8.

Scott DW, Anderson WI. 1991. Canine hair follicle neoplasms: a retrospective analysis of 80 cases (1986–1987). Veterinary Dermatology, 2: 143–50.

Scott, D.W., Miller, W.H., Griffin, C.E. 1995. Small Animal Dermatology, 5th Ed., WB Saunders, p756-757.

Scott, D. W., Miller, W. H., Griffin, C. E. 2001a. Congenital and hereditary defects. In: Muller & Kirk’s Small Animal Dermatology, 6^th^ edn (eds Scott, D.W., Miller, W.H., Griffin, C.E.), Saunders Elsevier, St Louis, Missouri, pp. 913-1003.

Scott, D. W., Miller, W. H., Griffin, C. E. 2001b. Skin immune system and allergic skin diseases. In: Muller & Kirk’s Small Animal Dermatology, 6^th^ edn (eds Scott, D.W., Miller, W.H., Griffin, C.E.), Saunders Elsevier, St Louis, Missouri, pp. 543-666.

Scott-Moncrieff, J.C., Treadwell, N.G., McCullough, S.M., Brooks, M.B. 2001. Hemostatic abnormalities in dogs with primary immune-mediated hemolytic anemia. J Am Anim Hosp Assoc. 37(3):220-7.

Searcy, G.P., Miller, D.R., Tasker, J.B. 1971. Congenital hemolytic anemia in the Basenji dog due to erythrocyte pyruvate kinase deficiency. Journal of Comparative Medicine 35: 67-70.

Selby, L. A., Becker, S. V., Hayes, H. W. 1981. Epidemiologic risk factors associated with canine systemic mycoses. American journal of epidemiology, 113(2), 133-139.

Senders, C.W., Eisele, P., Freeman, L.E., Sponenberg, D.P. 1986. Observations about the normal and abnormal embryogenesis of the canine lip and palate. J Craniofac Genet Dev Biol Suppl 2:241-8

Seppälä, E.H., Koskinen, L.L., Gulløv, C.H., Jokinen, P., Karlskov-Mortensen, P., Bergamasco, L., Baranowska Körberg, I., Cizinauskas, S., Oberbauer, A.M., Berendt, M., Fredholm, M., Lohi, H. 2012. Identification of a novel idiopathic epilepsy locus in Belgian Shepherd dogs. PLoS One. 2012;7(3):e33549. Epub Mar 23. PubMed PMID: 22457775.

Seppälä, E.H., Reuser, A.J., Lohi, H. 2013. A Nonsense Mutation in the Acid α-Glucosidase Gene Causes Pompe Disease in Finnish and Swedish Lapphunds. PLoS One. 8(2):e56825. doi: 10.1371/journal.pone.0056825.

Sevelius, E., Andersson, M., Jönsson, L. 1994. Hepatic accumulation of alpha-1-antitrypsin in chronic liver disease in the dog. Journal of comparative pathology, 111(4), 401-412.

Sewell, A.C., Moritz, A., Duran, M. 1997. D-(+)-glyceric aciduria in an Afghan hound. Journal of Inherited Metabolic Diseases. 20(3):395-6.

Shelton, G.D. 1999. Neuromuscular disorders affecting young dogs and cats. Veterinary Neurology and Neurosurgery Journal, 1,1.

Shiel, R.E., Mooney, C.T., Brennan, S.F., Nolan, C.M., Callanan, J.J. 2010. Clinical and clinicopathological features of non-suppurative meningoencephalitis in young greyhounds in Ireland. Vet Rec. 9: 333-337.

Silverman, B.S., Kuttel, J. 1982. Lung torsion in an Afghan Hound, 3. Mod Vet Pract 63 (3): 228-229.

Sisk, D.B., Levesque, D.C., Wood, P.A., Styer, E.L.1990. Clinical and pathologic features of ceroid lipofuscinosis in two Australian cattle dogs. J Am Vet Med Assoc. 1197(3): 361-4.

Skelly, C.M., McAllister, H., Donnelly, W.J. 1997. Avulsion of the tibial tuberosity in a litter of greyhound puppies. Journal of Small Animal Practice 38(10):445-9.

Slappendel, R.J. 1975. Hemophilia A and hemophilia B in a family of French bulldogs.Tijdschrift voor Diergeneeskunde. 100(20):1075-88.

Slatter, D.H., Blogg, J.R., Constable, I.J. 1980. Retinal degeneration in Greyhounds. Australian Veterinary Journal 56:106.

Slater, M.R., Scarlett, J.M., Kaderly, R.E., Bonnett, B.N. 1991. Breed, gender and age as risk factors for canine osteochondritis dissecans. Veterinary and Comparative Orthopaedics and Traumatology 4:100-106.

Smallwood, L.J., Barsanti, J.A. 1995. Hypoadrenocorticism in a family of leonbergers. Journal of the American Animal Hospital Association 31(4):301-5.

Smith, D. A., Knottenbelt, M. K. 1988. Spontaneous regression of intracutaneous cornifying epitheliomata in a dog. Journal of small animal practice, 29(3), 201-206.

Smith, F. O. 2006. Canine pyometra. Theriogenology, 66(3), 610-612.

Smith, B. F., Yue, Y., Woods, P. R., Kornegay, J. N., Shin, J. H., Williams, R. R., & Duan, D. 2011. An intronic LINE-1 element insertion in the dystrophin gene aborts dystrophin expression and results in Duchenne-like muscular dystrophy in the corgi breed. Laboratory Investigation, 91(2), 216-231.

Snaps, F.R., Heimann, M., Saunders, J., Beths, T., Balligand, M., Breton, L. 1998. Osteochondrosis of the sacral bone in a mastiff dog. Vet Rec. 143(17):476-7.

Somberg, R.L., Pullen, R.P., Casal, M.L., Patterson, D.F., Felsburg, P.J., Henthorn, P.J. 1995. A single nucleotide insertion in the canine interleukin-2 receptor gamma chain results in X-linked severe combined immunodeficiency disease. Veterinary Immunology and Immunopathology 47: 203-213.

Sommerlad, S., McRae, A.F., McDonald, B., Johnstone, I., Cuttell, L., Seddon, J.M., O'Leary, C.A. 2010. Congenital sensorineural deafness in Australian Stumpy-tail Cattle Dogs is an autosomal recessive trait that maps to CFA10. PLoS One, Oct 12;5(10):e13364.

Spiess, B. M. 1993. Inherited eye diseases in the Entlebucher mountain dog. Schweizer Archiv fur Tierheilkunde, 136(3), 105-110.

Sponenberg, D.P., Bowling, A.T. 1985. Heritable syndrome of skeletal defects in a family of Australian shepherd dogs. Journal of Heredity 76:393-394.

Stalis, I.H., Chadwick, B., Dayrell-Hart, B., Summers, B.A., Van Winkle, T.J. 1995. Necrotizing meningoencephalitis of Maltese dogs. Vet Pathol. 3:230-5.

Stannard, A.A., Pulley, L.T. 1975. Intracutaneous cornifying epithelioma (keratoacanthoma) in the dog: a retrospective study of 25 cases. Journal of the American Veterinary Medical Association. 167(5):385-8.

Strain, G.M. 1996. Aetiology, prevalence and diagnosis of deafness in dogs and cats [Review]. British Veterinary Journal 152: 17-36.

Strain, G.M. 2004. Deafness prevalence and pigmentation and gender associations in dog breeds at risk. Vet J. 167(1):23-32.

Strain, G.M., Clark, L.A., Wahl, J.M., Turner, A.E., Murphy, K.E. 2009. Prevalence of deafness in dogs heterozygous or homozygous for the merle allele. J Vet Intern Med. 23(2):282-6.

Starr, A.N., Famula, T.R., Markward, N.J., Baldwin, J.V., Fowler, K.D., Klumb, D.E., Simpson, N.L., Murphy, K.E. 2007. Hereditary evaluation of multiple developmental abnormalities in the Havanese dog breed. J Hered. 98(5):510-7.

Steinberg, K.S., Van Winkle, T., Bell, J.S., de Lahunta, A. 2000. Cerebellar degeneration in Old English Sheepdogs Journal of the American Veterinary Medical Association. 217:1162-1165.

Storey, E.S., Grahn, B.H., Alcorn, J. 2005. Multifocal chorioretinal lesions in Borzoi dogs. Vet Ophthalmol. ;8(5):337-47.

Studdert, V.P., Mitten, R.W. 1991. Clinical Features of Ceroid Lipofuscinosis in Border Collie Dogs. Australian Veterinary Journal 68: 137-140

Sueki, H., Shanley, K., Goldschmidt, M. H., Lazarus, G. S., Murphy, G. F. 1997. Dominantly inherited epidermal acantholysis in dogs, simulating human benign familial chronic pemphigus (Hailey–Hailey disease). British Journal of Dermatology, 136(2), 190-196.

Summers, B.A., Cummings, J.F., deLahunta, A. 1995. Veterinary Neuropathology. Mosby, St. Louis, MO 527

Swenson, L., Haggstrom, J., Kvart, C., Juneja, R.K. 1996. Relationship between parental cardiac status in Cavalier King Charles spaniels and prevalence and severity of chronic valvular disease in offspring. Journal of the American Veterinary Medical Association 208(12):2009-12.

Szczulowska, M. 1967. Dermoid cyst in the eye in relation to heredity and overfeeding. Medical Veterinarian 23:567.

Takada, K., Kitamura, H., Takiguchi, M., Saito, M., Hashimoto, A. 2002. Cloning of canine 21-hydroxylase gene and its polymorphic analysis as a candidate gene for congenital adrenal hyperplasia-like syndrome in Pomeranians. Res Vet Sci. 73(2):159-63.

Takeuchi, Y., Kaneko, F., Hashizume, C., Masuda, K., Ogata, N., Maki, T., ... & Mori, Y. 2009. Association analysis between canine behavioural traits and genetic polymorphisms in the Shiba Inu breed. Animal genetics, 40(5), 616-622.

Tanaka, R., Hoshi, K., Nagashima, Y., Fujii, Y., Yamane, Y. 2001. Detachable coils for occlusion of patent ductus arteriosus in 2 dogs. Veterinary Surgery 30(6):580-4.

Tanaka, H., Nakayama, M., Takase, K. 2003. Histiocytic ulcerative colitis in a French bulldog. J Vet Med Sci. 65(3):431-3.

Targett, M., McInnes, E. 1998. Afghan hound myelopathy. The Veterinary Record 142(25):704.

Temwichitr, J., Leegwater, P.A., Auriemma, E., Van't, Veld, E.M., Zijlstra, C., Voorhout, G., Hazewinkel, H.A. 2010. Evaluation of radiographic and genetic aspects of hereditary subluxation of the radial head in Bouviers des Flandres. Am J Vet Res. 71(8):884-90.

Temizsoylu, M.D., Avki, S. 2003. Complete ventral ankyloglossia in three related dogs. J Am Vet Med Assoc. 223(10):1443-5.

Teske, E., Naan, E. C., Van Dijk, E. M., Van Garderen, E., & Schalken, J. A. 2002. Canine prostate carcinoma: epidemiological evidence of an increased risk in castrated dogs. Molecular and cellular endocrinology, 197(1), 251-255.

Tidholm, A. 1997. Retrospective study of congenital heart defects in 151 dogs. Journal of Small Animal Practice 38: 94-98.

Tiemeyer, M.J., Singer, H.S., Troncoso, J.C., Cork, L.C., Coyle, J.T., Price, D.L.1984. Synaptic neurochemical alterations associated with neuronal degeneration in an inherited cerebellar ataxia of Gordon Setters.J Neuropathol Exp Neurol. 43(6):580-91.

Tipold, A., Fatzer, R., Jaggy, A., Moore, P., Vandevelde, M. 2000. Presumed immune-mediated cerebellar granuloprival degeneration in the Coton de Tulear breed.Journal of Neuroimmunology 110(1-2):130-3.

Tisdall, P.L., Hunt, G.B., Bellenger, C.R., Malik, R. 1994 Congenital portosystemic shunts in Maltese and Australian cattle dogs. Australian Veterinary Journal. 71(6):174-8.

Tobias KM, Rohrbach BW. (2003) Association of breed with the diagnosis of congenital portosystemic shunts in dogs: 2,400 cases (1980-2002). J Am Vet Med Assoc. 223(11):1636-9.

Torrez, C.V., Hunt, G.B. 2006. Results of surgical correction of abnormalities associated with brachycephalic airway obstruction syndrome in dogs in Australia. J Small Anim Pract. Mar;47(3):150-4.

Van der Linde-Sipman, J.S. 1987. Dysplasia of the pectinate ligament and primary glaucoma in the Bouvier de Flandres dog. Veterinary Pathology 24: 201.

Van der Woerdt, A., Stades, F. C., Van der Linde-Sipman, J. S., Boeve, M. H. 1995. Multiple ocular anomalies in two related litters of Soft Coated Wheaten Terriers. Veterinary and comparative ophthalmology, 5.

van Dongen, A.M., van Leeuwen, M., Slappendel, R.J. 2001. Canine von Willebrand's disease type 2 in German wirehair pointers in the Netherlands.Vet Rec. 148(3):80-2.

van Oost, B.A., Versteeg, S.A., Slappendel, R.J. 2004. DNA testing for type III von Willebrand disease in Dutch Kooiker dogs. J Vet Intern Med. 18(3):282-8.

van Rensberg, I.B.J., Petrick, S., Van der Lagt, J., Smit, M. 1992. Multiple inherited eye anomalies including persistent hyperplastic tunica vasculosa lentis in the Bouvier des Flandres. Progress in Veterinary and Comparative Ophthalmology 2:143.

van Steenbeek F.G., van den Bossche, L., Leegwater, P.A., Rothuizen, J.2011. Inherited liver shunts in dogs elucidate pathways regulating embryonic development and clinical disorders of the portal vein. Mamm Genome. Nov 4.

van Tongern, S.E., van Vonderen, I.K., van Nes, J.J., van den Ingh, T.S. 2000. Cerebellar cortical abiotrophy in two Portuguese Podenco littermates. Vet Quarterly 22(3):172-4.

Vandevelde, M., Braund ,K.G., Walder, T.L., Kornegay, J. 1978. Dysmyelination of the central nervous system in the Chow-Chow dog. Acta Neuropathologica 42: 211-215.

Vandevelde, M., Fatzer, R. 1980. Neuronal ceroid-lipofuscinosis in older dachshunds. Veterinary Pathology 17: 686-92

Varshney, J. P. 2007. Clinical management of idiopathic epilepsy in dogs with homeopathic< i> Belladonna</i> 200C: a case series. Homeopathy, 96(1), 46-48.

Vasseur, P.B., Foley, P., Stevenson, S., Heitter, D. 1989. Mode of inheritance of Perthes' disease in Manchester terriers. Clinical Orthopoedics (244):281-92

Venter, I. J., Van Der Lugt, J. J., vanRensburg, I. B. J., & Petrick, S. W. 1996. Multiple congenital eye anomalies in Bloodhound puppies. Vet Comp Ophthalmol, 6, 9-13.

Vianna, M. L., Tobias, K. M. 2005. Atresia ani in the dog: a retrospective study. Journal of the American Animal Hospital Association, 41(5), 317-322.

Vidgren, G., Vainio-Siukola, K., Honkasalo, S., Dillard, K., Anttila, M., Vauhkonen, H. 2012. Primary hyperoxaluria in Coton de Tulear. Anim Genet. 3:356-61. doi: 10.1111/j.1365-2052.2011.02260.

Villagomez, D.A., Alonso, R.A. 1998. A distinct Mendelian autosomal recessive syndrome involving the association of anotia, palate agenesis, bifid tongue, and polydactyly in the dog. Can Vet J. 39(10):642 3.

Villamil, J.A., Henry, C.J., Bryan, J.N., Ellersieck, M., Schultz, L., Tyler, J.W., Hahn, A.W. 2011. Identification of the most common cutaneous neoplasms in dogs and evaluation of breed and age distributions for selected neoplasms. J Am Vet Med Assoc. Oct 1;239(7):960-5.

von Bomhard, W., Mauldin, E.A., Schmutz, S.M., Leeb, T., Casal, M.L. 2006. Black hair follicular dysplasia in Large Munsterlander dogs: clinical, histological and ultrastructural features. Vet Dermatol. 17(3):182-8.

von Sandersleben, J., Hanichen, T., Fiebiger, I., Brem, G. 1986. Lipid storage disease similar to Wolman's disease in humans in the fox terrier. Tierarztliche Prax. 14: 253-63.

Waldman, L. 1995. Seasonal flank alopecia in affenpinschers. Journal of Small Animal Practice 36: 271-273.

Wang, X., Miller, A. B., Lepine, A. J., Scott, D. J., & Murphy, K. E. 1999. Analysis of randomly amplified polymorphic DNA (RAPD) for identifying genetic markers associated with canine hip dysplasia. Journal of heredity, 90(1), 99-103.

Wang, Z.H., Zeng, B., Shibuya, H., Johnson, G.S., Alroy, J., Pastores, G.M., Raghavan, S., Kolodny, E.H. 2000. Isolation and characterization of the normal canine beta-galactosidase gene and its mutation in a dog model of GM1-gangliosidosis. Journal of Inherited Metabolic Diseases. 23(6):593-606.

Watson, A.D.J., Dixon, R.T. 1977. Cystic bone lesions in related Old English Sheepdogs. Journal of Small Animal Practice 18: 561-571.

Watson, P.J., Herrtage, M.E., Peacock, M.A., Sargan, D.R. 1999. Primary ciliary dyskinesia in Newfoundland dogs. Veterinary Record 144: 718-25.

Watson, P., Narfström, K., Bedford, P. G. C. 1993. Retinal Pigment Epithelial Dystrophy (RPED) in Polish Lowland Sheepdogs. In British Small Animal Veterinary Association Congress, Birmingham.

Weaver, A. D. 1983. Survey with follow-up of 67 dogs with testicular sertoli cell tumours. Veterinary Record, 113(5), 105-107.

Weissenbock, H., Obermaier, G., Dahme, E. 1996. Alexander's disease in a Bernese mountain dog. Acta Neuropathologica (Berl). 91(2):200-4.

Weissl, J., Hülsmeyer, V., Brauer, C., Tipold, A., Koskinen, L.L., Kyöstilä, K., Lohi, H., Sauter-Louis, C., Wolf, M., Fischer, A. 2011. Disease Progression and Treatment Response of Idiopathic Epilepsy in Australian Shepherd Dogs. J Vet Intern Med. Dec 19. doi: 10.1111/j.1939-1676.2011.00853.x.

Wentink, G. H., Van der Linde-Sipman, J. S., Meijer, A. E. F. H., Kamphuisen, H. A. C., Van Vorstenbosch, C. J. A. H. V., Hartman, W., & Hendriks, H. J. 1972. Myopathy with a possible recessive X-linked inheritance in a litter of Irish Terriers. Veterinary Pathology Online, 9(5), 328-349.

Werner, P., Raducha, M.G., Prociuk, U., Ostrander, E.A., Spielman, R.S., Kirkness, E.F., Patterson, D.F., Henthorn, P.S. 2005. The keeshond defect in cardiac conotruncal development is oligogenic(1). Hum Genet. 2005 Feb 12.

Wheeler, S., Rusbridge, C. 1996. Neurological syndrome in Italian spinones. Vet Rec. 138(9):216.

Whitbread, T. J., Gill, J. J. B., Lewis, D. G. 1983. An inherited enchondrodystrophy in the English Pointer dog. A new disease. Journal of Small Animal Practice, 24(7), 399-411.

Whitney, K.M., Lothrop, C.D. 1995. Genetic test for pyruvate kinase deficiency of Basenjis. Journal of the American Veterinary Medical Association 207: 918-921.

Wiersma, A.C., Millon, L.V., van Dongen, A.M., van Oost, B.A., Bannasch, D.L. 2005. Evaluation of Canine COL4A3 and COL4A4 as Candidates for Familial Renal Disease in the Norwegian Elkhound. J Hered.

Wieczorek, L.A., Garosi, L.S., Shelton, G.D. 2006. Dystrophin-deficient muscular dystrophy in an old English sheepdog. Vet Rec. 158(8):270-3.

Wilbe, M., Sundberg, K., Hansen, I.R., Strandberg, E., Nachreiner, R.F., Hedhammar, A., Kennedy, L.J., Andersson, G., Björnerfeldt, S. 2010. Increased genetic risk or protection for canine autoimmune lymphocytic thyroiditis in Giant Schnauzers depends on DLA class II genotype. Tissue Antigens. 6:712-719.

Wilke, V.L., Conzemius, M.G., Kinghorn, B.P., Macrossan, P.E., Cai, W., Rothschild. M.F. 2006. Inheritance of rupture of the cranial cruciate ligament in Newfoundlands. J Am Vet Med Assoc. 228(1):61-4.

Williams, D.A., Maggio-Price, L. 1984. Canine idiopathic thrombocytopenic purpura: Clinical observations and long-term follow up in 54 cases. Journal of the American Veterinary Medical Association 185:660-663.

Williams, J.H., Duncan, N.M. 1986. Chylothorax with concurrent right cardiac lung lobe torsion in an Afghan hound. J S Afr Vet Assoc. 57(1):35-7.

Willis, M.B. 2000. Dew claw removal in Bernese mountain dogs. Vet Rec. 2000 Jul 15;147(3):84.

Wininger, F.A., Zeng, R., Johnson, G.S., Katz, M.L., Johnson, G.C., Bush, W.W., Jarboe, J.M. and Coates, J.R. 2011. Degenerative Myelopathy in a Bernese Mountain Dog with a Novel SOD1 Missense Mutation. Journal of Veterinary Internal Medicine, 25: 1166–1170. doi: 10.1111/j.1939-1676.2011.0760.x;

Witsberger, T. H., Villamil, J. A., Schultz, L. G., Hahn, A. W., Cook, J. L. 2008. Prevalence of and risk factors for hip dysplasia and cranial cruciate ligament deficiency in dogs. Journal of the American Veterinary Medical Association, 232(12), 1818-1824.

Wood, P.A., Sisk, D.B., Styer, E., Baker, H. 1987. Animal model: ceroidosis (ceroid-lipofuscinosis) in Australian cattle dogs. American Journal of Medical Genetics 26: 891-898.

Wood, S.L., Patterson, J.S. 2001. Shetland Sheepdog leukodystrophy. Journal of Veterinary Internal Medicine.15(5):486-93.

Wood, F. D., Pollard, R. E., Uerling, M. R., Feldman, E. C. 2007. Diagnostic imaging findings and endocrine test results in dogs with pituitary-dependent hyperadrenocorticism that did or did not have neurologic abnormalities: 157 cases (1989–2005). Journal of the American Veterinary Medical Association, 231(7), 1081-1085.

Wright, J.A., Brownlie, S. 1985. Progressive ataxia in a Pyrenean mountain dog. Vet Rec. 116(15):410-1.

Wright, J.A., Smyth, J.B., Brownlie, S.E., Robins, M. 1987. A myopathy associated with muscle hypertonicity in Cavalier King Charles Spaniels. Journal of Comparative Pathology 97: 559-565.

Yaeger, M.J., Majercik, K., Carter, M., Rothschild, M. 2000. An autosomal recessive, lethal, neurologic disease of Gordon Setter puppies. Journal Veterinary Diagnostic Investigation 12(6):570-3.

Yakely, W.L. 1971. Familial cataracts in the American cocker spaniel. Journal of the American Animal Hospital 7:127-129.

Yamato, O., Lee, K.W., Chang, H.S., Tajima, M., Maede, Y. 1999. Relation between erythrocyte reduced glutathione and glutamate concentrations in Korean Jindo dogs with erythrocytes possessing hereditary high activity of Na-K-ATPase and a high concentration of potassium. Journal of Veterinary Medical Science 61: 1179- 182.

Yamato, O., Ochiai, K., Masuoka, Y., Hayashida, E., Tajima, M., Omae, S., Iijima, M., Umemura, T., Maede, Y. 2000. GM1 gangliosidosis in shiba dogs. Vet Rec. 146(17):493-6.

Yearley, J.H., Hancock, D.D., Mealey, K.L. 2004. Survival time, lifespan, and quality of life in dogs with idiopathic Fanconi syndrome. J Am Vet Med Assoc. 225(3):377-83.

Yuzbasiyan-Gurkan, V., Blanton, S.H., Cao, Y., Ferguson, P., Li, J., Venta, P.J., Brewer, G.J. 1997. Linkage of a microsatellite marker to the canine copper toxicosis locus in Bedlington terriers. American Journal of Veterinary Research 58:23-7.

Zangerl, B., Goldstein, O., Philp, A.R., Lindauer, S.J., Pearce-Kelling, S.E., Mullins, R.F., Graphodatsky, A.S., Ripoll, D., Felix, J.S., Stone, E.M., Acland, G.M., Aguirre, G.D. 2006. Identical mutation in a novel retinal gene causes progressive rod-cone degeneration in dogs and retinitis pigmentosa in humans. Genomics, 88(5):551-63.

Zheng, K., Thorner, P.S., Marrano, P., Baumal, R., McInnes, R.R. 1994. Canine X chromosome-linked hereditary nephritis: a genetic model for human X-linked hereditary nephritis resulting from a single base mutation in the gene encoding the alpha 5 chain of collagen type IV. Proceedings of the National Academy of Science USA 91(9):3989-93.

Zhou, Z., Sheng, X., Zhang, Z., Zhao, K., Zhu, L., Guo, G., Friedenberg, S.G., Hunter, L.S., Vandenberg-Foels, W.S., Hornbuckle, W.E., Krotscheck, U., Corey, E., Moise, N.S., Dykes, N.L., Li, J., Xu, S., Du, L., Wang, Y., Sandler, J., Acland, G.M., Lust, G., Todhunter, R.J. 2010. Differential genetic regulation of canine hip dysplasia and osteoarthritis. PLoS One. 11;5(10):e13219.
